# Green Synthesis and Antifungal Activities of Novel *N*-Aryl Carbamate Derivatives

**DOI:** 10.3390/molecules29153479

**Published:** 2024-07-25

**Authors:** Xiyao Liu, Yuyao Sun, Lifang Liu, Xufei Duan, Shujun You, Baojia Yu, Xiaohong Pan, Xiong Guan, Ran Lin, Liyan Song

**Affiliations:** 1 Key Laboratory of Biopesticide and Chemical Biology, Ministry of Education, College of Plant Protection, Fujian Agriculture and Forestry University, Fuzhou 350002, China; t1930@ndnu.edu.cn (X.L.); cgdltdz31415@126.com (Y.S.); liulifangduty@163.com (L.L.); 15297340771@163.com (X.D.); 13385093571@163.com (S.Y.); 18350057238@163.com (B.Y.); panxiaohong@163.com (X.P.); guanxfafu@126.com (X.G.); 2 Fujian Provincial University Key Laboratory of Green Energy and Environment Catalysts, College of New Energy and Materials, Ningde Normal University, Ningde 352100, China; 3 College of Bee Science and Biomedicine, Fujian Agriculture and Forestry University, Fuzhou 350002, China

**Keywords:** carbamate derivatives, green synthesis, phytopathogenic fungi, antifungal activity, structure–activity relationship

## Abstract

Carbamate is a key structural motif in the development of fungicidal compounds, which is still promising and robust in the discovery of green pesticides. Herein, we report the synthesis and evaluation of the fungicidal activity of 35 carbamate derivatives, among which 19 compounds were synthesized in our previous report. These derivatives were synthesized from aromatic amides in a single step, which was a green oxidation process for Hofmann rearrangement using oxone, KCl and NaOH. Their chemical structures were characterized by ^1^H NMR, ^13^C NMR and high-resolution mass spectrometry. Their antifungal activity was tested against seven plant fungal pathogens. Many of the compounds exhibited good antifungal activity in vitro (inhibitory rate > 60% at 50 μg/mL). Compound **1ag** exhibited excellent broad-spectrum antifungal activities with inhibition rates close to or higher than 70% at 50 μg/mL. Notably, compound **1af** demonstrated the most potent inhibition against *F. graminearum*, with an EC_50_ value of 12.50 μg/mL, while compound **1z** was the most promising candidate fungicide against *F. oxysporum* (EC_50_ = 16.65 μg/mL). The structure–activity relationships are also discussed in this paper. These results suggest that the *N*-aryl carbamate derivatives secured by our green protocol warrant further investigation as potential lead compounds for novel antifungal agents.

## 1. Introduction

Plant pathologists have contributed to the germ theory of plant disease [1]. Phytopathogenic fungi can directly or indirectly cause a remarkable decline in the quality and quantity of plant production in both pre- and post-harvest situations [2]. James G. Horsfall specialized in fungicides and made a great contribution to that field [3]. So far, the use of fungicides is the most efficient, convenient, cost-effective and widely applicable method for the management of plant disease, playing a vital role in safeguarding our crops and improving the yield [4,5]. These are considered as the second and last line of defense after host resistance in the fight against plant diseases. However, long-term usage of traditional fungicides in agriculture has resulted in the development of severe drug resistance [6]. As a result, the discovery of new and innovative fungicides is driven by the fact that novel fungicides are an essential element to provide sustained control of major crop diseases [7].

**Figure 1:**
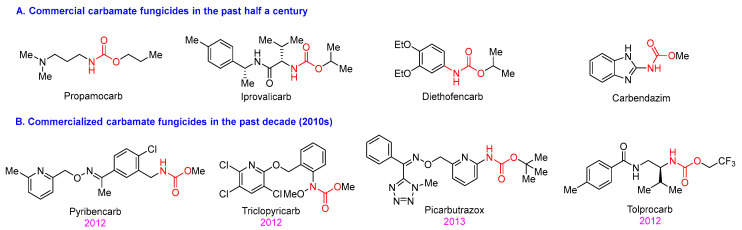
Commercialized fungicides containing carbamate motif.

Carbamate is a key structural motif, representing a series of broad-spectrum bioactive compounds that have been widely used as pesticides worldwide. In addition, the carbamate group is valuable in drug discovery and medicinal chemistry for its good chemical stability and easy delivery into cells [8]. Carbamates could be hydrolyzed smoothly to afford amine, carbon dioxide and alcohol, which are more easily excreted from mammals. In addition, the degradation of carbamates in the environment is rapid and causes little residue problem. [9]. It highly preserves the potential for developing carbamate fungicides as green and ecofriendly pesticides. A number of commercially available carbamate fungicides have been successfully developed and widely applied in the market during the past half a century (Figure 1A). These carbamate compounds exhibit broad-spectrum antifungal activities by targeting the thiol groups of the enzymes of plant pathogenic fungi [10].

Noteworthy is that the carbamate motif is still a promising and robust pharmacophore in the discovery of green pesticides and has attracted significant attention from both the industry and academia. A number of novel fungicides containing the carbamate motifs (Pyribencarb [11], Triclopyricarb [12], Picarbutrazox [13] and Tolprocarb [14]) were commercialized in the past decade (2010s) as a new generation of carbamate fungicides (Figure 1B). As a result, the synthetic community paid continuous attention to the synthesis and antifungal activities of carbamates for the R&D of green fungicides [15,16,17,18,19,20,21,22].

**Figure 2:**
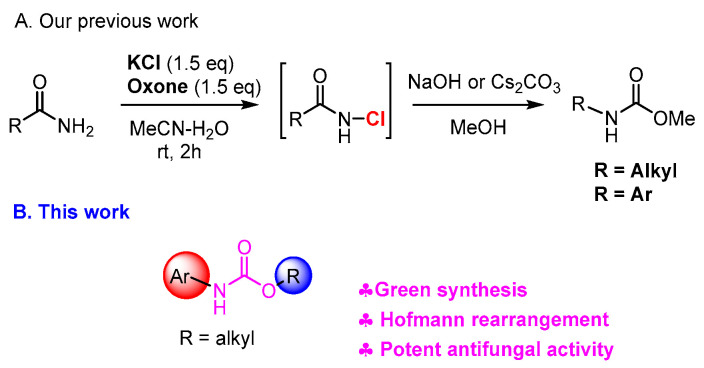
Design of novel carbamate derivatives.

To date, the main synthetic approaches to carbamates include phosgene and its derivatives (i.e., isocyanate, chloroformate, dimethyl carbonate), the carbonylation method, the carbon dioxide method and the alcoholysis of urea. These methods suffer from toxic, expensive or inconvenient reagents or procedures [23]. In previous work, we developed the first green oxidation of amides with oxone and chloride to generate *N*-chloro amides, which rearranged to isocyanate intermediates and subsequently produced stable carbamates (Hofmann rearrangement) when trapped with alcohols (Figure 2A) [24].

With the powerful synthetic method in hand, we would like to report novel carbamate derivatives and antifungal screening (Figure 2B). This work culminated in the employment of green Hofmann rearrangement with oxone, chlorides and inorganic bases. Motivated by these, 35 carbamate derivatives were designed and synthesized. The antifungal activities of the target compounds were evaluated against seven fungal plant pathogens, namely, *Botrytis cinerea* (*B. cinerea*), *Magnaporthe grisea* (*M. grisea*), *Pythium aphanidermatum* (*P. aphanidermatum*), *Fusarium graminearum* (*F. graminearum*), *Valsa mali* (*V. mali*), *Colletotrichum siamense* (*C. siamense*) and *Fusarium oxysporum* (*F. oxysporum*). And the structure–activity relationships (SARs) were also summarized by analyzing the experimental results of the antifungal activities.

## 2. Results

### 2.1. Chemistry

**Scheme 1:**
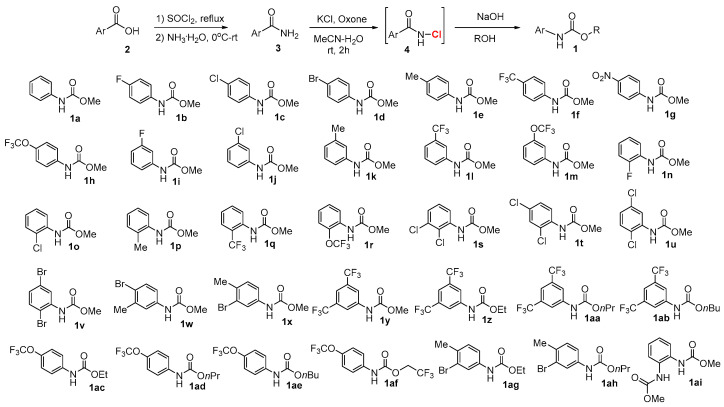
Synthesis of carbamate analogues.

A total of thirty-five synthesized carbamate derivatives were prepared. The synthetic pathway for the carbamate derivatives is outlined in Scheme 1. Commercially available aromatic acids **2** were converted to acyl chloride intermediates in refluxing thionyl chloride (SOCl_2_), which were further elaborated to aromatic amides **3** by ammonolysis. With sufficient aromatic amides in hand, we carried out green Hofmann rearrangement in a one pot, two steps manner. According to the known procedure [24], aromatic amides **3** were transformed into *N*-chloride intermediates (**4**) with oxone and potassium chloride, which were treated with sodium hydroxide to rearrange to isocyanate to give carbamates when trapped with different alcohols. Noteworthy is that various *O*-alkyl carbamates were prepared in addition to *N*-aryl carbamates. The chemical structure of the title compounds was characterized and confirmed by ^1^H NMR, ^13^C NMR, HRMS and ^19^F NMR (when necessary).

### 2.2. In Vitro Antifungal Activity

The antifungal activities of compounds **1a**–**1ai** against seven phytopathogenic fungi were screened in vitro by the mycelial growth inhibition method [25]. The fungicidal effects of the target compounds were investigated against various fungal species, including *B. cinerea*, *M. grisea*, *P. aphanidermatum*, *F. graminearum*, *V. mali*, *C. siamense* and *F. oxysporum*, with the commercialized fungicide azoxystrobin serving as a positive control. The preliminary antifungal activity screening was carried out at a concentration of 50 μg/mL and the corresponding results are summarized in Table 1. Almost half of the compounds exhibited significant antifungal activities against the seven phytopathogenic fungi (inhibition rate > 50%). According to the data presented in Table 1, six target compounds showed favorable antifungal activities against *B. cinerea* with inhibition rates exceeding 60%, which was superior to that of azoxystrobin (54.39%). Among them, compounds **1t**, **1x** and **1ag** gave outstanding inhibition rates higher than 70%. For *M. grisea*, up to six target compounds demonstrated good antifungal activities (inhibition rate > 60%), which were better than that of azoxystrobin (59.94%). The same three compounds (**1t**, **1x**, **1ag**) exhibited inhibition rates of over 70%. Eight compounds exhibited high efficacies against *P. aphanidermatum* with an inhibition rate of over 60%. Among them, compounds **1x** and **1ag** revealed good inhibitory activities of 72.48% and 75.4%, respectively, which were significantly higher than that of the control azoxystrobin (56.40%). As far as *F. graminearum* was concerned, compounds **1t**, **1y**, **1z**, **1ac**, **1ad**, **1af** and **1ag** displayed remarkable inhibitory activities above 70%. In particular, compounds **1t** and **1af** were found to be the most potent ones, with an inhibition rate of 93.61% and 85.83%, achieving a 30% higher inhibition rate in comparison to that of the positive control (azoxystrobin). Furthermore, eight of the title compounds had similar or higher antifungal activities against *V. mali* compared with the control azoxystrobin. Regarding *C. siamense*, only four compounds, namely **1t**, **1w**, **1x** and **1ag**, exhibited notable fungicidal activity with inhibition rates of 69.97%, 65.22%, 68.08%, 72.12%, respectively, which were superior to that of azoxystrobin (54.79%). When the antifungal activities of title compounds against *F. oxysporum* were studied, only three compounds **1t**, **1z** and **1ag** exerted better activities than azoxystrobin (62.50%). In general, compounds **1s**, **1t**, **1z** and **1ag** exhibited noteworthy broad-spectrum antifungal activities against the seven fungi. Particularly, compound **1ag** demonstrated excellent antifungal effects against all the fungi, with inhibition rates close to or higher than 70%, which were better than the positive control using commercial agent azoxystrobin.

To investigate the inhibitory performances of the target compounds against phytopathogenic fungi in more detail, the EC_50_ values of compounds with favorable inhibition rates at 50 μg/mL were tested. As shown in Table 2, the title compounds showed medium to good antifungal activities against *M. grisea* and *V. mali*. As far as *B. cinerea* and *P. aphanidermatum* were concerned, compound **1ag** showed the highest activity (EC_50_ = 29.44 μg/mL and 24.64 μg/mL), whose activity was inferior but close to that of commercial fungicide azoxystrobin (18.06 μg/mL and 13.99 μg/mL). Notably, the EC_50_ value of compound **1af** against *F. graminearum* (12.50 μg/mL) was very close to that of azoxystrobin (10.36 μg/mL), while the EC_50_ value of compound **1z** against *F. oxysporum* (16.65 μg/mL) was very close to that of azoxystrobin (14.14 μg/mL). Considering the simple structures and feasibility for further functionalization, these compounds can be good lead compounds for the further optimization of broad-spectrum fungicides. Remarkably, compound **1ag** exhibited superior and more extensive antifungal activity compared to that of other compounds, positioning it as a prime candidate for further investigation.

## 3. Discussion

The chemical structures of compounds **1s**–**1y** were interpreted by carefully analyzing the aromatic peaks of the ^1^H NMR spectra in combination with the chemical structure of the starting materials (commercially available aromatic acids or amides), respectively, while the methyl carbamate moiety was easily characterized by the OMe peak and broad single peak of the NH. The chemical structure could be further substantiated by the ^13^C NMR spectra. Moreover, we confirmed the chemical structures of compounds **1aa**–**1ae** and **1ag**–**1ah** by analyzing the aliphatic peaks of the ^1^H NMR and ^13^C NMR spectra in comparison with **1z**, **1h** and **1x**, for the alkyl chain length of carbamate changed, while the aromatic part remained intact in these cases.

By analyzing the results in Table 1, we found that the fungi (*B. cinerea*, *M. grisea*, *P. aphanidermatum*, *F. graminearum* and *V. mali*) were more sensitive to our title compounds than *C. siamense* and *F. oxysporum*. The structure–activity relationship of the target compounds in vitro was analyzed based on the results listed in Table 1 and Table 2. Firstly, we came to single-substituted *N*-aryl carbamates. Among the *para*-substituted compounds, we observed that neither the electron-donating substituents nor the electron-withdrawing ones enhanced the antifungal activity of the compounds. The introduction of trifluoromethyl (CF_3_) or trifluoromethoxy (CF_3_O) improved the antifungal activity (**1f**, **1h**), which might be attributed to the fact that the incorporation of CF_3_ or OCF_3_ into the proper positions of agrochemicals can markedly enhance their lipophilicity, chemical and metabolic stability, bioavailability and other properties due to the robust electrophilic properties [26,27]. As the *para*-substituent changed to the *meta*position of the phenyl ring, the antifungal activities decreased slightly. To our surprise, the single substituents on the *ortho* position of the *N*-phenyl ring almost eliminated all the antifungal activities despite the presence of CF_3_ or OCF_3_.

Then we came to the di-substituted *N*-aryl carbamates. The presence of halogen was beneficial to the antifungal activities and led to fruitful results. The 2,3-dichloro (**1s**) and 2,4-dichloro (**1t**) *N*-aryl carbamates exhibited promising antifungal activities and were superior to the 2,5-dichloro (**1u**) or 2,5-dibromo (**1v**) *N*-aryl carbamates. Both the 4-bromo-3-methyl (**1w**) and 3-bromo-4-methyl (**1x**) *N*-aryl carbamates displayed excellent antifungal activities, while 3-bromo-4-methyl (**1x**) was slightly better. The 3,5-ditrifluoromethyl *N*-aryl carbamate (**1z**) also exhibited good antifungal activities. Moreover, adding one more carbamate functional group did not significantly improve the antifungal activity (**1ai**). On the other hand, it showed almost the lowest antifungal activity. The findings indicated that the groups and positions of substituents on the *N*-phenyl ring had a significant impact on the antifungal activities.

Lastly, we investigated the structure–activity relationship concerning the alkyl chain length. By observing the antifungal activities of compounds **1y**–**1ab** (possessing identical aromatic moiety), we found that compound **1z** (R = Et) out-performed the other compounds. This result suggested that ethyl group might be the proper length of the alkyl R group and further extension of the carbon chain to *n*-propyl or *n*-butyl group would dramatically decrease the antifungal activity. Similar trends could be observed concerning the corresponding compounds [(**1h**, **1ac**–**1ae**) and (**1x**, **1ag**–**1ah**)]. Noteworthy is that the introduction of trifluoromethyl (CF_3_) to the end of ethyl group (**1af**) would increase the EC_50_ against *F. graminearum*, while it did not show higher antifungal activity against the other phytopathogenic fungi.

## 4. Materials and Methods

### 4.1. General Information

All the commercially available reagents and solvents were purchased from commercial sources and used directly. ^1^H and ^13^C nuclear magnetic resonance (NMR) spectra were measured using Bruker AVIII 400 MHz spectrometers (Bruker, Rheinstetten, Germany). ^1^H NMR and ^13^C NMR chemical shifts are reported in ppm (*δ*) with the solvent (CDCl_3_) peaks employed as the internal standard (7.26 ppm for ^1^H and 77.16 ppm for ^13^C). Data are reported as follows: chemical shift, multiplicity (s = singlet, brs = broad singlet, d = doublet, t = triplet, q = quartet, m = multiplet), coupling constants (Hz) and integration. High resolution mass spectra (HRMS) were produced by electron spray ionization (ESI) on a Thermo Scientific LTQ Orbitrap XL instrument (ThermoFisher, MA, USA) or a Bruker solariX instrument.

### 4.2. Synthetic Procedures

#### 4.2.1. Synthesis of Amides **3**

The corresponding amides **3** were prepared by ammonolysis of aromatic acyl chlorides, which could be easily accessed by commercially available aromatic acids and thionyl chloride. The experimental details can be found in the Supplementary Materials.

#### 4.2.2. Synthesis of Carbamates **1**

Carbamate **1** was synthesized following our previous report [24]. The desired compounds **1a**–**1r** and **1ai** were known compounds [24] and already available in our laboratory. Moreover, compounds **1s**–**1ah** were prepared accordingly, among which five compounds (**1s**, **1t**, **1u**, **1z**, **1af**) were known compounds and the corresponding references were added, while the rest of the compounds are newly synthesized. The characterization data of compounds **1s**–**1ah** are listed as follows:

**1s [28]**. 50 mg, 75% yield; white solid. m.p. = 103–105 °C. **^1^H NMR** (400 MHz, CDCl_3_) δ: 8.11 (dd, *J* = 7.7, 1.7 Hz, 1H), 7.24 (br, 1H), 7.18 (d, *J* = 8.1 Hz, 1H), 7.15 (dd, *J* = 8.1, 1.8 Hz, 1H), 3.80 (s, 3H). **^13^C NMR** (100 MHz, CDCl_3_) δ: 153.6, 136.5, 132.8, 127.9, 124.4, 120.7, 117.8, 52.8. **HRMS** (ESI) *m*/*z* calculated for C_8_H_8_O_2_NCl_2_^+^ [M + H]^+^ 219.9927, found 219.9924.

**1t [28]**. 53 mg, 80% yield; white solid. m.p. = 68–69 °C. **^1^H NMR** (400 MHz, CDCl_3_) δ: 8.14 (d, *J* = 8.9 Hz, 1H), 7.36 (d, *J* = 2.4 Hz, 1H), 7.25 (dd, *J* = 8.9, 2.4 Hz, 1H), 7.12 (br, 1H), 3.82 (s, 3H). **^13^C NMR** (100 MHz, CDCl_3_) δ: 153.6, 133.6, 128.8, 128.3, 128.0, 122.6, 120.7, 52.8. **HRMS** (ESI) *m*/*z* calculated for C_8_H_8_O_2_NCl_2_^+^ [M + H]^+^ 219.9927, found 219.9930.

**1u [28]**. 51 mg, 77% yield; white solid. m.p. = 72–74 °C. **^1^H NMR** (400 MHz, CDCl_3_) δ: 8.26 (d, *J* = 2.3 Hz, 1H), 7.24 (d, *J* = 8.6 Hz, 1H), 7.15 (br, 1H), 6.96 (dd, *J* = 8.6, 2.5 Hz, 1H), 3.81 (s, 3H). **^13^C NMR** (100 MHz, CDCl_3_) δ: 153.4, 135.6, 133.7, 129.8, 123.7, 112.0, 119.7, 52.9. **HRMS** (ESI) *m*/*z* calculated for C_8_H_8_O_2_NCl_2_^+^ [M + H]^+^ 219.9927, found 219.9925.

**1v**. *Methyl (2,5-dibromophenyl)carbamate*. Purified by flash chromatography on silica gel using eluents (hexane/ethyl acetate = 10/1 to 5/1). 72 mg, 78% yield; white solid. m.p. = 98–100 °C. **^1^H NMR** (400 MHz, CDCl_3_) δ: 8.44–8.33 (m, 1H), 7.36 (d, *J* = 8.5 Hz, 1H), 7.13 (br, 1H), 7.06 (dd, *J* = 8.6, 2.3 Hz, 1H), 3.81 (s, 3H). **^13^C NMR** (100 MHz, CDCl_3_) δ: 153.5, 137.0, 133.3, 127.3, 122.9, 122.2, 110.9, 52.9. **HRMS** (ESI) *m*/*z* calculated for C_8_H_8_O_2_NBr_2_^+^ [M + H]^+^ 307.8916, found 307.8918.

**1w**. *Methyl (4-bromo-3-methylphenyl)carbamate*. Purified by flash chromatography on silica gel using eluents (hexane/ethyl acetate = 10/1 to 5/1). 59 mg, 81% yield; white solid. m.p. = 70–72 °C. **^1^H NMR** (400 MHz, CDCl_3_) δ: 7.40 (d, *J* = 8.6 Hz, 1H), 7.28 (s, 1H), 7.13–7.05 (m, 1H), 6.90 (br, 1H), 3.76 (s, 3H), 2.33 (s, 3H). **^13^C NMR** (100 MHz, CDCl_3_) δ: 154.1, 138.6, 137.2, 132.6, 121.0, 118.6, 117.8, 52.5, 23.1. **HRMS** (ESI) *m*/*z* calculated for C_9_H_11_O_2_NBr^+^ [M + H]^+^ 243.9968, found 243.9963.

**1x**. *Methyl (3-bromo-4-methylphenyl)carbamate*. Purified by flash chromatography on silica gel using eluents (hexane/ethyl acetate = 10/1 to 5/1). 60 mg, 82% yield; white solid. m.p. = 100–102 °C. **^1^H NMR** (400 MHz, CDCl_3_) δ:7.63 (s, 1H), 7.21 (d, *J* = 8.3 Hz, 1H), 7.13 (d, *J* = 8.2 Hz, 1H), 6.74 (br, 1H), 3.77 (s, 3H), 2.33 (s, 3H). **^13^C NMR** (100 MHz, CDCl_3_) δ: 154.1, 136.7, 132.8, 130.9, 124.9, 122.5, 117.8, 52.6, 22.3. **HRMS** (ESI) *m*/*z* calculated for C_9_H_11_O_2_NBr^+^ [M + H]^+^ 243.9968, found 243.9963.

**1y**. *Methyl (3,5-bis(trifluoromethyl)phenyl)carbamate*. Purified by flash chromatography on silica gel using eluents (hexane/ethyl acetate = 10/1 to 5/1). 68 mg, 79% yield; white solid. m.p. = 116–118 °C. **^1^H NMR** (400 MHz, CDCl_3_) δ: 7.90 (s, 2H), 7.56 (s, 1H), 7.02 (br, 1H), 3.82 (s, 3H). **^13^C NMR** (100 MHz, CDCl_3_) δ: 153.7, 139.5, 132.6 (q, *J* = 33.3 Hz, 2 × C), 123.2 (q, *J* = 271.1 Hz, 2 × C), 118.3 (2 × C), 116.9 (q, *J* = 4.0 Hz), 53.1. ^**19**^**F NMR** (376 MHz, CDCl_3_) δ: −63.07. **HRMS** (ESI) *m*/*z* calculated for C_10_H_6_O_2_NF_6_^−^ [M-H]^−^ 286.0308, found 286.0304.

**1z [29]**. 71 mg, 78% yield; white solid. m.p. = 93–95 °C. **^1^H NMR** (400 MHz, CDCl_3_) δ: 7.89 (s, 2H), 7.55 (s, 1H), 6.97 (br, 1H), 4.27 (q, *J* = 7.1 Hz, 2H), 1.33 (t, *J* = 7.1 Hz, 3H). **^13^C NMR** (100 MHz, CDCl_3_) δ: 153.3, 139.7, 132.6 (q, *J* = 33.3 Hz, 2 × C), 123.2 (q, *J* = 271 Hz, 2 × C), 118.3 (2 × C), 116.8 (q, *J* = 4.0 Hz), 62.2, 14.5. ^**19**^**F NMR** (376 MHz, CDCl_3_) δ: −63.1 (6 × F). **HRMS** (ESI) *m*/*z* calculated for C_11_H_10_O_2_NF_6_^+^ [M + H]^+^ 302.0610, found 302.0609.

**1aa**. *Propyl (3,5-bis(trifluoromethyl)phenyl)carbamate*. Purified by flash chromatography on silica gel using eluents (hexane/ethyl acetate = 10/1 to 5/1). 72 mg, 76% yield; white solid. m.p. = 73–75 °C. **^1^H NMR** (400 MHz, CDCl_3_) δ: 7.89 (s, 2H), 7.54 (s, 1H), 7.05 (br, 1H), 4.16 (td, *J* = 6.7, 1.9 Hz, 2H), 1.71 (qd, *J* = 7.1, 1.9 Hz, 2H), 0.98 (td, *J* = 7.4, 1.8 Hz, 3H). **^13^C NMR** (100 MHz, CDCl_3_) δ: 153.5, 139.8, 132.5 (q, *J* = 33.3 Hz, 2 × C), 123.3 (q, *J* = 287 Hz, 2 × C), 118.4 (2 × C), 116.8 (q, *J* = 3.1 Hz), 67.9, 22.4, 10.5. **^19^F NMR** (376 MHz, CDCl_3_) δ: −63.1 (6 × F). **HRMS** (ESI) *m*/*z* calculated for C_12_H_12_O_2_NF_6_^+^ [M + H]^+^ 316.0767, found 316.0759.

**1ab**. *Butyl (3,5-bis(trifluoromethyl)phenyl)carbamate*. Purified by flash chromatography on silica gel using eluents (hexane/ethyl acetate = 10/1 to 5/1). 73 mg, 74% yield; white solid. m.p. = 59–61 °C. **^1^H NMR** (400 MHz, CDCl_3_) δ: 7.92 (s, 2H), 7.56 (s, 1H), 7.12 (br, 1H), 4.23 (t, *J* = 6.7 Hz, 2H), 1.69 (dq, *J* = 8.6, 6.7 Hz, 2H), 1.51–1.38 (m, 2H), 0.97 (t, *J* = 7.4 Hz, 3H). ^**13**^**C NMR** (100 MHz, CDCl_3_) δ: 153.5, 139.7, 132.6 (q, *J* = 33.3 Hz, 2 × C), 123.4 (q, *J* = 287 Hz, 2 × C), 118.3 (2 × C), 116.7 (q, *J* = 3.1 Hz), 66.1, 30.9, 19.2, 13.8. **^19^F NMR** (376 MHz, CDCl_3_) δ: −63.1 (6 × F). **HRMS** (ESI) *m*/*z* calculated for C_13_H_14_O_2_NF_6_^+^ [M + H]^+^ 330.0923, found 330.0924.

**1ac**. *Ethyl (4-(trifluoromethoxy)phenyl)carbamate*. Purified by flash chromatography on silica gel using eluents (hexane/ethyl acetate = 10/1 to 5/1). 63 mg, 84% yield; white solid. m.p. = 73–74 °C. **^1^H NMR** (400 MHz, CDCl_3_) δ: 7.40 (d, *J* = 8.5 Hz, 2H), 7.21–7.12 (m, 2H), 6.65 (br, 1H), 4.23 (q, *J* = 7.1 Hz, 2H), 1.31 (t, *J* = 7.1 Hz, 3H). **^13^C NMR** (100 MHz, CDCl_3_) δ: 153.6, 144.8, 136.8, 122.1 (2 × C), 120.5 (q, *J* = 255.1 Hz),119.7 (2 × C), 61.6, 14.7. **^19^F NMR** (376 MHz, CDCl_3_) δ: −58.2 (3×F). **HRMS** (ESI) *m*/*z* calculated for C_10_H_11_O_3_NF_3_^+^ [M + H]^+^ 250.0686, found 250.0686.

**1ad**. *Propyl (4-(trifluoromethoxy)phenyl)carbamate*. Purified by flash chromatography on silica gel using eluents (hexane/ethyl acetate = 10/1 to 5/1). 64 mg, 81% yield; white solid. m.p. = 84–85 °C. **^1^H NMR** (400 MHz, CDCl_3_) δ: 7.42 (d, *J* = 8.5 Hz, 2H), 7.15 (d, *J* = 8.6 Hz, 2H), 6.80 (br, 1H), 4.13 (t, *J* = 6.7 Hz, 2H), 1.70 (p, *J* = 7.1 Hz, 2H), 0.97 (dd, *J* = 7.9, 7.0 Hz, 3H). **^13^C NMR** (100 MHz, CDCl_3_) δ: 153.8, 144.7 (d, *J* = 2.0 Hz), 136.9, 122.0 (2 × C),120.5 (q, *J* = 255.0 Hz), 119.8 (2 × C), 67.2, 22.3, 10.4. **^19^F NMR** (376 MHz, CDCl_3_) δ: −58.2 (3 × F). **HRMS** (ESI) *m*/*z* calculated for C_11_H_13_O_3_NF_3_^+^ [M + H]^+^ 264.0842, found 264.0844.

**1ae**. *Butyl (4-(trifluoromethoxy)phenyl)carbamate*. Purified by flash chromatography on silica gel using eluents (hexane/ethyl acetate = 10/1 to 5/1). 65 mg, 78% yield; white solid. m.p. = 74–76 °C. **^1^H NMR** (400 MHz, CDCl_3_) δ: 7.43 (d, *J* = 8.6 Hz, 2H), 7.17 (d, *J* = 8.6 Hz, 2H), 6.83 (br, 1H), 4.19 (t, *J* = 6.7 Hz, 2H), 1.73–1.59 (m, 2H), 1.49–1.37 (m, 2H), 0.97 (t, *J* = 7.4 Hz, 3H). **^13^C NMR** (100 MHz, CDCl_3_) δ: 153.8, 144.7 (d, *J* = 2.0 Hz), 136.9, 122.0 (2 × C), 120.6 (q, *J* = 255.0 Hz), 119.8 (2 × C), 65.5, 31.0, 19.2, 13.8. **^19^F NMR** (376 MHz, CDCl_3_) δ: −58.2 (3 × F). **HRMS** (ESI) *m*/*z* calculated for C_12_H_15_O_3_NF_3_^+^ [M + H]^+^ 278.0999, found 278.0998.

**1af** [30]. 75 mg, 82% yield; yellowish solid. m.p. = 71–72 °C. **^1^H NMR** (400 MHz, CDCl_3_) δ: 7.43 (d, *J* = 8.5 Hz, 2H), 7.23–7.16 (m, 2H), 6.95 (br, 1H), 4.57 (q, *J* = 8.4 Hz, 2H). **^13^C NMR** (100 MHz, CDCl_3_) δ: 151.6, 145.5, 135.7, 122.2 (2 × C), 123.0 (q, *J* = 275 Hz), 120.6 (q, *J* = 255 Hz), 120.2 (2 × C), 61.2 (q, *J* = 27.2 Hz). **^19^F NMR** (376 MHz, CDCl_3_) δ: −74.2 (3 × F), −58.3 (3 × F). **HRMS** (ESI) *m*/*z* calculated for C_10_H_8_O_3_NF_6_^+^ [M + H]^+^ 304.0403, found: 304.0398.

**1ag**. *Ethyl (3-bromo-4-methylphenyl)carbamate*. Purified by flash chromatography on silica gel using eluents (hexane/ethyl acetate = 10/1 to 5/1). 64 mg, 83% yield; white solid. m.p. = 68–70 °C. **^1^H NMR** (400 MHz, CDCl_3_) δ: 7.64 (s, 1H), 7.24–7.16 (m, 1H), 7.13 (d, *J* = 8.2 Hz, 1H), 6.61 (br, 1H), 4.22 (q, *J* = 7.1 Hz, 2H), 2.33 (s, 3H), 1.30 (t, *J* = 7.1 Hz, 3H). **^13^C NMR** (100 MHz, CDCl_3_) δ: 153.6, 136.9, 132.8, 130.9, 125.0, 122.5, 117.7, 61.5, 22.3, 14.7. **HRMS** (ESI) *m*/*z* calculated for C_10_H_13_O_2_NBr^+^ [M + H]^+^ 258.0124, found 258.0119.

**1ah**. *Propyl (3-bromo-4-methylphenyl)carbamate*. Purified by flash chromatography on silica gel using eluents (hexane/ethyl acetate = 10/1 to 5/1). 65 mg, 80% yield; white solid. m.p. = 41–43 °C. **^1^H NMR** (400 MHz, CDCl_3_) δ: 7.64 (s, 1H), 7.20 (d, *J* = 8.4 Hz, 1H), 7.13 (d, *J* = 8.2 Hz, 1H), 6.64 (br, 1H), 4.12 (t, *J* = 6.7 Hz, 2H), 2.33 (s, 3H), 1.69 (q, *J* = 7.2 Hz, 2H), 0.97 (t, *J* = 7.4 Hz, 3H). ^**13**^**C NMR** (100 MHz, CDCl_3_) δ: 153.7, 136.9, 132.7, 130.9, 125.0, 122.5, 117.7, 67.1, 22.4, 22.3, 10.5. **HRMS** (ESI) *m*/*z* calculated for C_11_H_15_O_2_NBr^+^ [M + H]^+^ 272.0281, found 272.0275.

### 4.3. Bioassays

The in vitro fungicidal activity of the synthesized title compounds were evaluated by the mycelial growth inhibition method according to the literature [25]. These selected strains included plant pathogenic fungi (*Botrytis cinerea*, *Magnaporthe oryzae*, *Pythium aphanidermatum*, *Fusarium graminearum*, *Valsa mali*, *Colletotrichum siamense* and *Fusarium oxysporum*). The specific experimental procedure is displayed in the Supplementary Materials.

## 5. Conclusions

In summary, in the search for novel, highly effective fungicide surrogates, we have synthesized 35 carbamate derivatives efficiently through our newly developed green synthetic methodology, among which 19 compounds were synthesized in our previous report [24], and systematically evaluated their inhibitory effects against seven important phytopathogenic fungi. The carbamate derivatives were fully characterized by ^1^H NMR, ^13^C NMR, HRMS and ^19^F NMR (when necessary). The results showed that many of the compounds exhibited good antifungal activity in vitro (inhibitory rate > 60% at 50 μg/mL, shown in Table 1). Compounds **1s**, **1t**, **1z** and **1ag** exhibited noteworthy broad-spectrum antifungal activities and compound **1ag** demonstrated excellent antifungal effects against all the fungi, with inhibition rates close to or higher than 70% at 50 μg/mL, which were better than the positive control using the commercial agent azoxystrobin. The in vitro bioassays identified that compound **1af** was a promising inhibitor of *F. graminearum*, with an EC_50_ value of 12.50 μg/mL, which was close to that of the control azoxystrobin (10.36 μg/mL). And compound **1z** exhibited excellent antifungal efficiency against *F. oxysporum* (16.65 μg/mL), comparable to that of the commercial fungicide azoxystrobin (14.14 μg/mL). From the systematic structure–activity relationship investigation, we found the halogen (including CF_3_) as well as the proper substitution pattern were critical to the di-substituted N-Aryl carbamates, while the ethyl group (Et) was the best O-alkyl substituent. In a word, those novel and promising carbamate derivatives could be regarded as a candidate for developing more potent fungicides to control pathogen fungi.

## Figures and Tables

**Table 1:** Preliminary in vitro antifungal activity of compounds against seven fungi at 50 μg/mL.

**Compound**	**Average Inhibition Rate ± SD (%) (n = 3) ^a^**						
**No.**	*B. c.*	*M. o.*	*P. a.*	***F. g.***	***V. m.***	***C. s.***	***F. o.***
**1a**	0	0.44 ± 1.38	2.96 ± 3.35	16.86 ± 1.76	2.36 ± 2.9	11.92 ± 9.83	25.2 ± 0.92
**1b**	1.33 ± 1.52	3.31 ± 7.28	3.27 ± 2.15	4.98 ± 0.64	9.36 ± 0.83	9.09 ± 0.71	25.32 ± 1.56
**1c**	18.42 ± 2.46	12.67 ± 1.38	27.86 ± 1.4	37.16 ± 0.81	31.24 ± 0.93	20.05 ± 0.53	31.24 ± 8.07
**1d**	42.58 ± 1.72	43.46 ± 0.28	43.14 ± 0.67	51.58 ± 0.72	52.94 ± 1.19	29.1 ± 1.62	42.03 ± 0.49
**1e**	13.32 ± 3.08	11.22 ± 0.29	16.68 ± 2.73	22.4 ± 3.46	13.47 ± 5.24	15.71 ± 0.73	27.66 ± 1.64
**1f**	55.98 ± 2.19	53.03 ± 0.85	55.77 ± 1.24	64.1 ± 2.2	68.91 ± 1.56	41.1 ± 1.29	43.26 ± 1.09
**1g**	0	0	0	0.88 ± 0.94	3.15 ± 2.95	11.88 ± 8.81	27.03 ± 1.79
**1h**	64.44 ± 0.95	64.82 ± 1.15	64.33 ± 0.81	54.27 ± 0.66	61.69 ± 0.77	24.79 ± 1.35	20.46 ± 0.28
**1i**	10.52 ± 1.79	7.79 ± 1.57	15.9 ± 1.69	39.17 ± 2.39	24.73 ± 1.29	18.65 ± 1.62	18.69 ± 7.94
**1j**	32.70 ± 1.37	28.77 ± 0.87	29.93 ± 1.34	45.89 ± 1.58	37.38 ± 1.57	37.07 ± 0.99	42.09 ± 0.71
**1k**	4.09 ± 0.68	0	4.33 ± 1.33	4.6 ± 0.83	6.82 ± 1	23.22 ± 1.65	20.74 ± 1.01
**1l**	43.64 ± 1.59	40.56 ± 0.75	49.18 ± 2	56.44 ± 1.63	42.43 ± 1.14	47.72 ± 0.93	36.78 ± 1.04
**1m**	57.94 ± 0.73	52.05 ± 1.16	49.64 ± 4.23	52.52 ± 0.82	34.78 ± 1.35	30.01 ± 0.99	26.55 ± 0.51
**1n**	0	0	3.27 ± 2.7	13.25 ± 1.75	0	0	25.84 ± 1.02
**1o**	7.12 ± 0.86	1.33 ± 1.33	0.11 ± 0.65	0	0	0	0
**1p**	5.28 ± 3.11	0	0	16.73 ± 22.9	7.53 ± 7.73	0.89 ± 4.54	1.25 ± 0.48
**1q**	0	0	14.6 ± 2.24	22.25 ± 4.3	5.01 ± 4.77	2.6 ± 6.49	0
**1r**	0.6 ± 2.45	0	0.22 ± 0.8	0	0	0	3.31 ± 1.26
**1s**	55.76 ± 1.26	55.16 ± 1.32	60.37 ± 3.15	53.41 ± 3.88	52.08 ± 0.56	52.67 ± 1.35	59.07 ± 3.2
**1t**	71.93 ± 1.74	74.18 ± 0.58	69.96 ± 3.93	93.61 ± 0.21	70.72 ± 0.43	69.97 ± 1.86	67.89 ± 0.28
**1u**	13.05 ± 2.42	13.12 ± 1.06	14.05 ± 0.34	36.79 ± 0.35	16.09 ± 0.56	6.98 ± 2.58	18.22 ± 1.25
**1v**	25.57 ± 1.42	29.71 ± 3.35	36.6 ± 0.51	28.15 ± 0.93	23.03 ± 1.9	20.38 ± 1.52	33.14 ± 0.48
**1w**	67.49 ± 1.58	67.49 ± 0.67	69.78 ± 1.62	49.82 ± 2.84	71.99 ± 0.42	65.22 ± 1.69	56.92 ± 0.96
**1x**	70.16 ± 1.52	70.25 ± 0.65	72.48 ± 0.12	48.89 ± 2.01	72.06 ± 0.52	68.08 ± 1.62	61.92 ± 0.42
**1y**	47.88 ± 0.81	54.82 ± 1.34	57.02 ± 0.6	74.55 ± 1.18	53.1 ± 0.23	43.13 ± 2.77	52.73 ± 0.21
**1z**	61.42 ± 0.49	63.38 ± 0.63	64.12 ± 0.46	77.6 ± 2.6	73.77 ± 1.21	58.76 ± 0.6	66.35 ± 0.55
**1aa**	27.31 ± 0.76	32.29 ± 1.29	32.28 ± 0.35	46.44 ± 0.49	38.05 ± 0.79	22.23 ± 0.82	32.12 ± 0.95
**1ab**	6.77 ± 0.41	3.03 ± 0.45	7.35 ± 0.54	19.8 ± 4.98	5.48 ± 0.47	0.26 ± 0.44	6.92 ± 1.77
**1ac**	54.48 ± 0.57	54.67 ± 0.6	55.51 ± 1.14	77.85 ± 4.53	55.12 ± 0.3	51.3 ± 0.61	40.86 ± 0.73
**1ad**	44.31 ± 0.24	46.56 ± 1.14	47.3 ± 0.56	70.07 ± 0.57	46.4 ± 0.13	40.42 ± 0.25	24.15 ± 4.07
**1ae**	31.89 ± 0.75	31.73 ± 1.15	30.76 ± 1.02	55.45 ± 1.42	36.96 ± 1.34	27.13 ± 0.81	11.8 ± 0.55
**1af**	49.05 ± 0.45	52.80 ± 0.58	50.00 ± 0.69	85.83 ± 1.55	52.73 ± 1.75	45.51 ± 0.51	34.03 ± 0.82
**1ag**	73.94 ± 0.61	72.72 ± 0.96	75.4 ± 0.45	73.37 ± 2.76	76.78 ± 0.47	72.12 ± 0.66	68.42 ± 0.48
**1ah**	62.07 ± 0.54	55.04 ± 0.14	61.56 ± 1.01	69.09 ± 0.88	63.91 ± 1.05	56.09 ± 4.16	50.58 ± 0.95
**1ai**	10.05 ± 0.67	5.67 ± 1.1	19.67 ± 6.49	16.77 ± 7.16	6.24 ± 1.73	16.49 ± 1.17	11.1 ± 0.89
**AZO**	54.39 ± 0.24	59.94 ± 0.24	56.40 ± 0.87	58.17 ± 0.22	63.14 ± 0.66	54.79 ± 0.67	62.50 ± 2.14

^a^*B. c.*, *Botrytis cinerea*; *M. g.*, *Magnaporthe grisea*; *P. a.*, *Pythium aphanidermatum*; *F. g.*, *Fusarium graminearum*; *V. m.*, *Valsa mali*; *C. s.*, *Colletotrichum siamense*; *F. o.*, *Fusarium oxysporum*; AZO, azoxystrobin. The different colors in the table reflected the different inhibition rates, which could be interpreted by the following explanation: 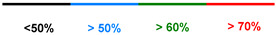.

**Table 2:** EC_50_ values (μg/mL) of selected compounds against *B. cinerea*, *M. grisea*, *P. aphanidermatum*, *F. graminearum*, *V. mali*, *F. oxysporum* in vitro.

Compound	Regression Equation	R^2^	EC_50_ **(μg/mL, 95% CI) ^a^**	EC_50_ **(μM, 95% CI) ^a^**
*B. cinerea*				
**1t**	Y = 2.04x + 1.86	0.972	34.05 (32.25–42.39)	0.1548 (0.1466–0.1927)
**1x**	Y = 2.89x + 0.48	0.994	36.55 (31.91–40.25)	0.1498 (0.1308–0.1650)
**1ag**	Y = 2.41x + 1.15	0.974	29.44 (25.66–34.33)	0.1141 (0.0995–0.1331)
**azoxystrobin**	Y = 0.38x + 4.52	0.917	18.06 (4.97–37.44)	0.0448 (0.0123–0.0928)
*M. grisea*				
**1t**	Y = 1.65x + 2.62	0.998	27.34 (24.54–34.22)	0.1243 (0.1115–0.1555)
**1x**	Y = 2.60x + 0.98	0.995	34.53 (32.70–40.98)	0.1415 (0.1340–0.1680)
**1ag**	Y = 2.03x + 1.99	0.997	29.98 (27.23–36.05)	0.1162 (0.1055–0.1397)
**azoxystrobin**	Y = 0.32x + 4.70	0.917	7.90 (0.08–17.84)	0.0196 (0.0002–0.0443)
*P. aphanidermatum*				
**1x**	Y = 2.22x + 1.73	0.974	29.22 (27.04–35.04)	0.1198 (0.1108–0.1436)
**1ag**	Y = 1.95x + 2.27	0.993	24.64 (20.27–27.40)	0.0955 (0.0503–0.1062)
**azoxystrobin**	Y = 0.33x + 4.61	0.968	13.99 (1.08–30.50)	0.0347 (0.0027–0.0757)
*F. graminearum*				
**1t**	Y = 2.42x + 1.83	0.955	20.22 (13.71–30.14)	0.0919 (0.0623–0.1370)
**1ac**	Y = 2.23x + 2.03	0.977	21.17 (19.31–25.15)	0.0850 (0.0776–0.1010)
**1af**	Y = 1.78x + 3.04	0.993	12.50 (10.38–14.93)	0.0413 (0.0343–0.0493)
**azoxystrobin**	Y = 0.34x + 4.65	0.971	10.36 (0.48–21.80)	0.0257 (0.0012–0.0541)
*V. mali*				
**1t**	Y = 1.80x + 2.35	0.989	28.96 (25.63–34.98)	0.1316 (0.1165–0.1590)
**1w**	Y = 2.45x + 1.52	0.902	26.23 (13.61–53.10)	0.1075 (0.0558–0.2176)
**1x**	Y = 2.24x + 1.75	0.993	27.87 (25.61–33.17)	0.1142 (0.1050–0.1359)
**1ag**	Y = 2.06x + 2.05	0.991	26.69 (23.65–31.17)	0.1034 (0.0917–0.1208)
**azoxystrobin**	Y = 0.5x + 4.42	0.958	4.71 (0.06–11.32)	0.0117 (0.0002–0.0281)
*F. oxysporum*				
**1t**	Y = 1.74x + 2.42	0.975	30.03 (28.35–38.60)	0.1365 (0.1289–0.1755)
**1z**	Y = 0.94x + 3.84	0.885	16.65 (5.71–38.79)	0.0553 (0.0188–0.1288)
**1ag**	Y = 1.74x + 2.63	0.996	22.71 (21.29–29.07)	0.0880 (0.0825–0.1127)
**azoxystrobin**	Y = 0.50x + 4.42	0.977	14.14 (5.66–23.54)	0.0351 (0.0140–0.0584)

^a^ Average of three replicates.

## Data Availability

Data are contained within the article and Supplementary Materials.

## Supplementary Materials

The following supporting information can be downloaded at: https://www.mdpi.com/article/10.3390/molecules29153479/s1, Copies of ^1^H, ^13^C spectra of new compounds.
