# Supplementary material for: Green Synthesis and Antifungal Activities of Novel N-Aryl Carbamate Derivatives
Source: Molecules. 2024 Jul 25;29(15):3479. doi: 10.3390/molecules29153479 (PMC11313961; doi:10.3390/molecules29153479)
Supplement: Supplementary file 1 [file molecules-29-03479-s001.zip › molecules-3130274-supplementary.pdf]

Supporting Information

Green synthesis and antifungal activities of novel *N*-aryl carbamate derivatives

Xiyao Liu [2†], Yuyao Sun [1†], Lifang Liu [1], Xufei Duan [1], Shujun You [1], Baojia Yu [1], Xiaohong Pan [1], Xiong Guan [1], Ran Lin [3,*] and Liyan Song [1,*]

[1] Key Laboratory of Biopesticide and Chemical Biology, Ministry of Education, College of Plant Protection, Fujian Agriculture and Forestry University, Fuzhou 350002, China
[2] Fujian Provincial University Key Laboratory of Green Energy and Environment Catalysts, College of New Energy and Materials, Ningde Normal University, Ningde 352100, China
[3] College of Bee Science and Biomedicine, Fujian Agriculture and Forestry University, Fuzhou 350002, China
† These authors contributed equally to this work.

linran@fafu.edu.cn, songliyan@fafu.edu.cn

Table of Content

The details of synthesis of carbamates **1**	**S-2**
In vitro antifungal activity evaluation	**S-3**
^{1}H NMR, ^{13}C NMR and HRMS Spectra of Synthesized Compounds	**S-4**

The details of synthesis of carbamates 1

1. Synthesis of amides **3**

The substituted aromatic acid (10 mmol) was dissolved in thionyl chloride ($SOCl_2$, 10 mL) and heated to reflux for 30 min and cooled to room temperature. The volatiles (mainly $SOCl_2$) were removed under reduced pressure and the resulting residue was dissolved in dry toluene (10 mL) and added dropwise to the pre-cooled ammonia aqueous solution (20 mL, ice bath). The resulting mixture was allowed to warm to room temperature and stirred overnight. The organic phase was collected and the aqueous phase was extracted with ethyl acetate (3 ×10 mL). The combined organic fractions were washed with water and brine, dried over anhydrous Na_2SO_4 and evaporated under reduced pressure. The residue was purified by flash column chromatography on silica gel using eluents (petroleum ether/acetone = 2/1) to afford the desired amides **3**.

2. Synthesis of carbamates **1**

To a stirred solution of aromatic amide substrate (**3**, 0.3 mmol) in the MeCN/H_2O (1/10, 1.1 mL) at 0 ℃ were added KCl (34 mg, 0.45 mmol) and Oxone (138 mg, 0.45 mmol). After completion of the addition, the resulting mixture was stirred for 10 min before warmed to room temperature and stirred for an additional 2 h. When the aromatic amide substrate was fully consumed as determined by TLC analysis, corresponding alkyl alcohol (0.6 mL) and NaOH (18 mg, 0.45 mmol) was added sequentially. The mixture was allowed to be stirred for 5 hrs and quenched by addition of sat. aq. Na_2SO_3 (5 mL). The volatiles (mainly alkyl alcohol and MeCN) was removed under reduced pressure and the aqueous mixture was extracted with ethyl acetate (3 × 10 mL). The combined organic fractions were washed with brine, dried over Na_2SO_4, and concentrated under reduced pressure. The residue was purified by flash column chromatography on silica gel using eluents (petroleum ether/ethyl acetate = 10/1) to provide the desired carbamates **1**.

In vitro antifungal activity evaluation

Compounds were dissolved in DMSO (0.5 ml) before mixing with Potato Dextrose Agar (PDA 99.5 ml) medium. The final concentration of compounds **1–2** in the medium were fixed at 50 μg/ml. Three kinds of fungi were incubated in PDA at 25 °C for five days to get a new mycelium for the antifungal assay, then a mycelia disk of an approximately 0.45 cm diameter cut from the culture medium was picked up with a sterilized inoculation needle and inoculated in the center of the PDA plate. The inoculated plates were incubated at 25 °C for five days. DMSO in sterilized distilled water served as the control, while azoxystrobin was used as a positive control for each treatment, three replicates were carried out. The radial growth of the fungal colonies was measured on the sixth day and the data were statistically analyzed. The relative control efficacy of compounds compared to the blank assay was calculated via the following equation: I (%) = [(CK−PT/CK)] × 100%, where I is the relative control efficacy, CK is the average disease index during the blank assay, and PT is the average disease index after treatment during testing. The *in vitro* inhibiting effects of the test compounds on the fungi were calculated by the formula CV = (A−B)/A, where A represents the diameter of fungi growth on untreated PDA, B represents the diameter of fungi on treated PDA, and CV represents the rate of inhibition. All of the strains were conserved in the Key Laboratory of Biopesticide and Chemical Biology, Ministry of Education, Fujian Agriculture and Forestry University (Fuzhou, China).

Based on the results of *in vitro* antifungal activity, the more active compounds were selected to determine their median effective concentration (EC_{50}) according to the same method described above. The stock solution was mixed with the autoclaved PDA medium to prepare a set of mediums containing 100, 50, 25, 12.5, 6.25, 3.125 μg/mL of the tested compound. Similarly, 0.5% DMSO in culture medium was used as a blank control. Each test was performed in triplicate. EC_{50} values and their confidence intervals at 95% probability (95% CI) were calculated by using the basic EC_{50} program version SPSS 22.0.

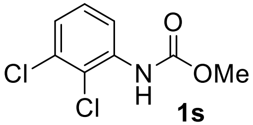

1s

^1H NMR (400 MHz, $CDCl_3$)

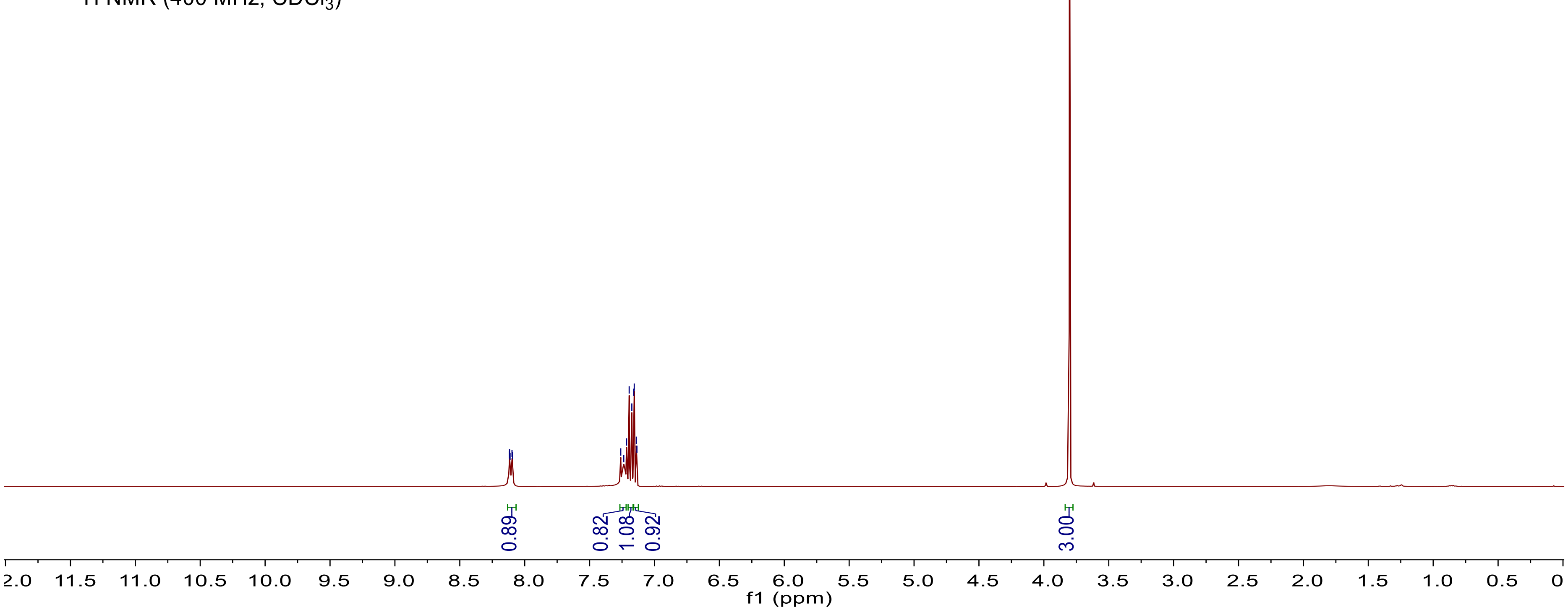

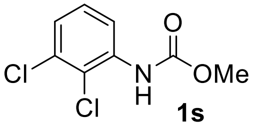

1s

^{13}C NMR (100 MHz, $CDCl_3$)

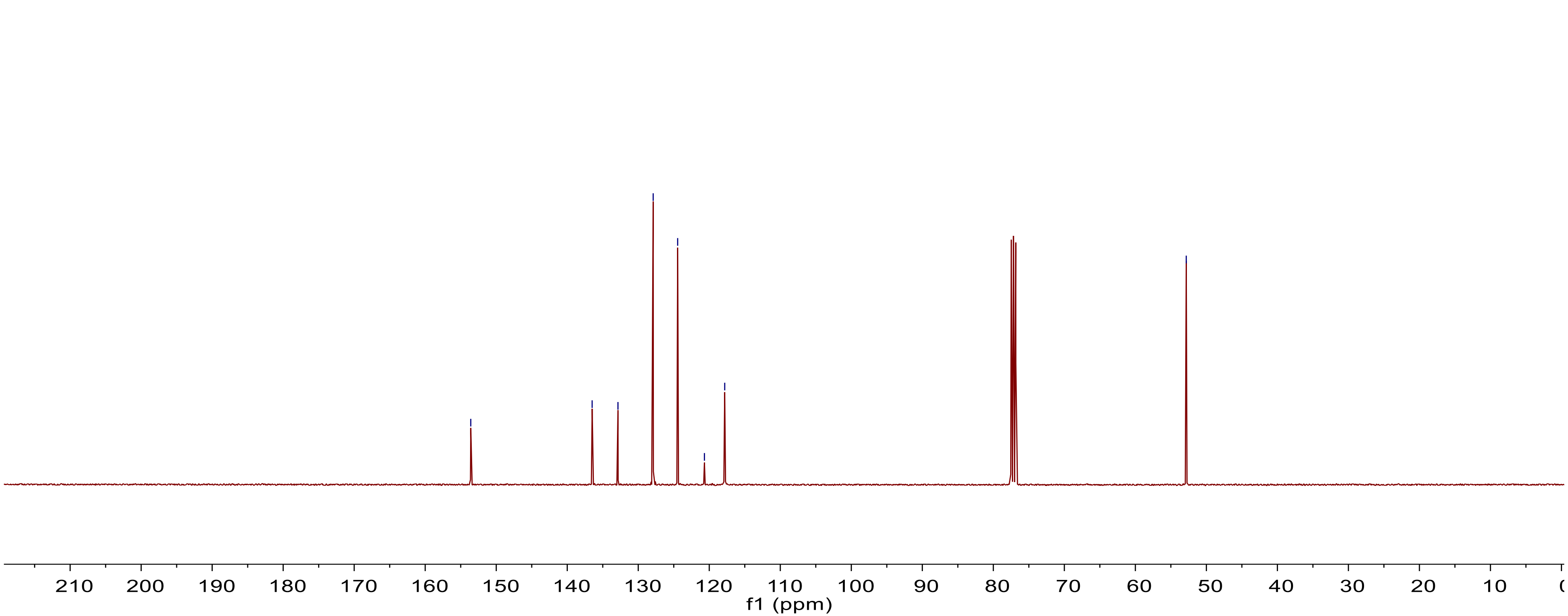

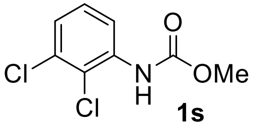

1s

HRMS (ESI)

SCIEX OS version: 3.0.0.3339 **Printed by:** DESKTOP-SI1BPI6/CZHG
Workstation ID: DESKTOP-SI1BPI6 **Printed on:** 7/13/2024 3:32:49 PM

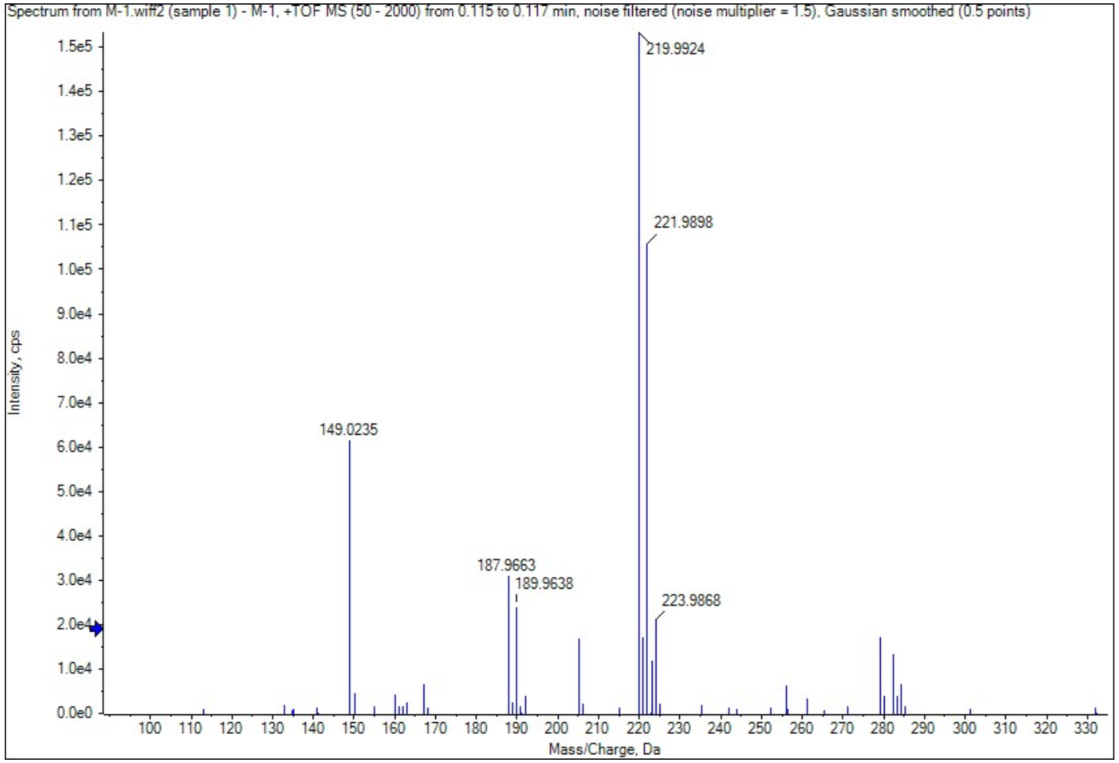

Page 1 of 1

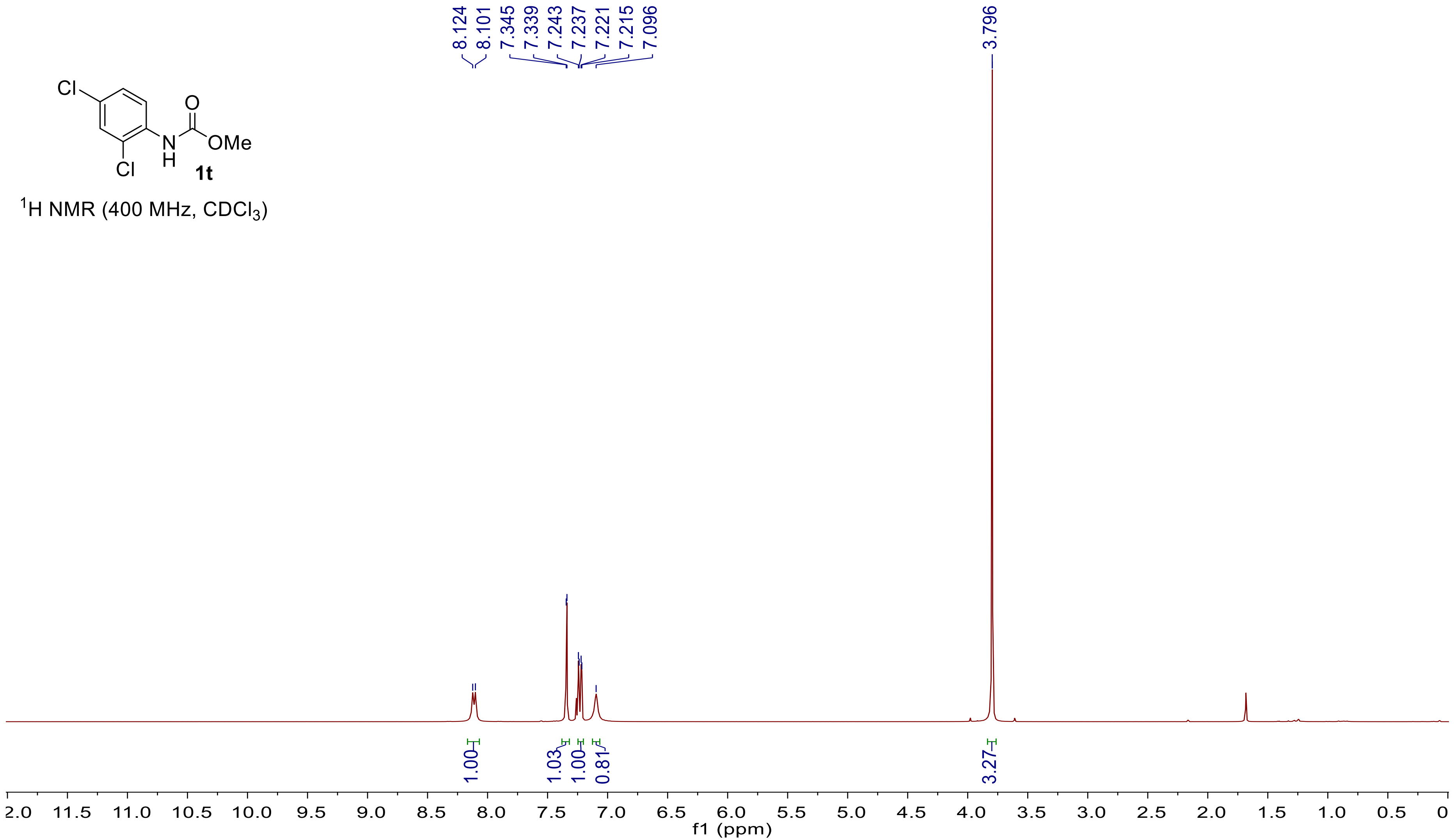

^1H NMR (400 MHz, $CDCl_3$)

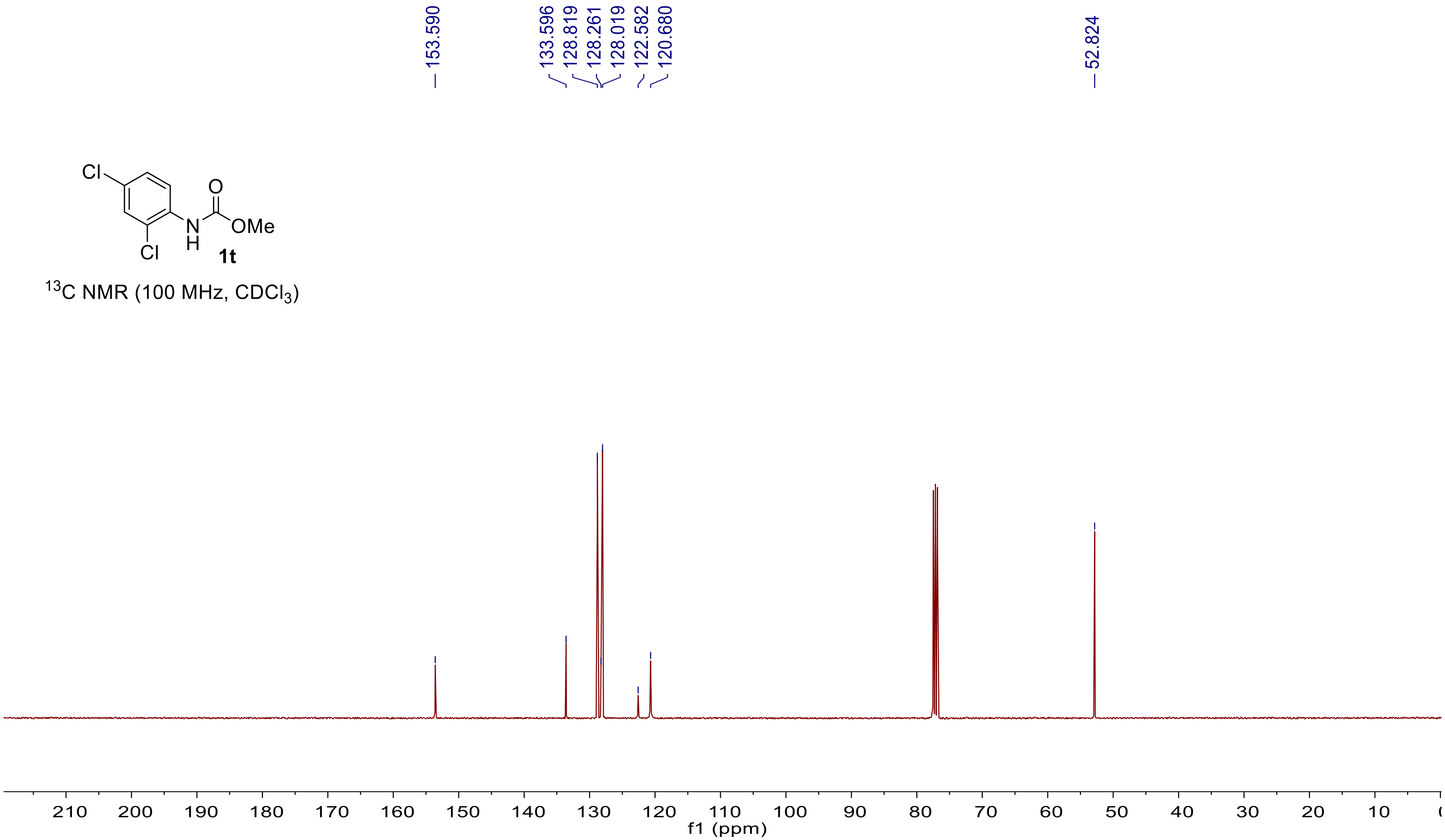

1t

^{13}C NMR (100 MHz, $CDCl_3$)

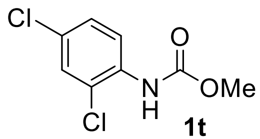

1t

HRMS (ESI)

SCIEX

SCIEX OS version: 3.0.0.3339 **Printed by:** DESKTOP-SI1BPI6/CZHG

Workstation ID: DESKTOP-SI1BPI6 **Printed on:** 7/13/2024 3:33:28 PM

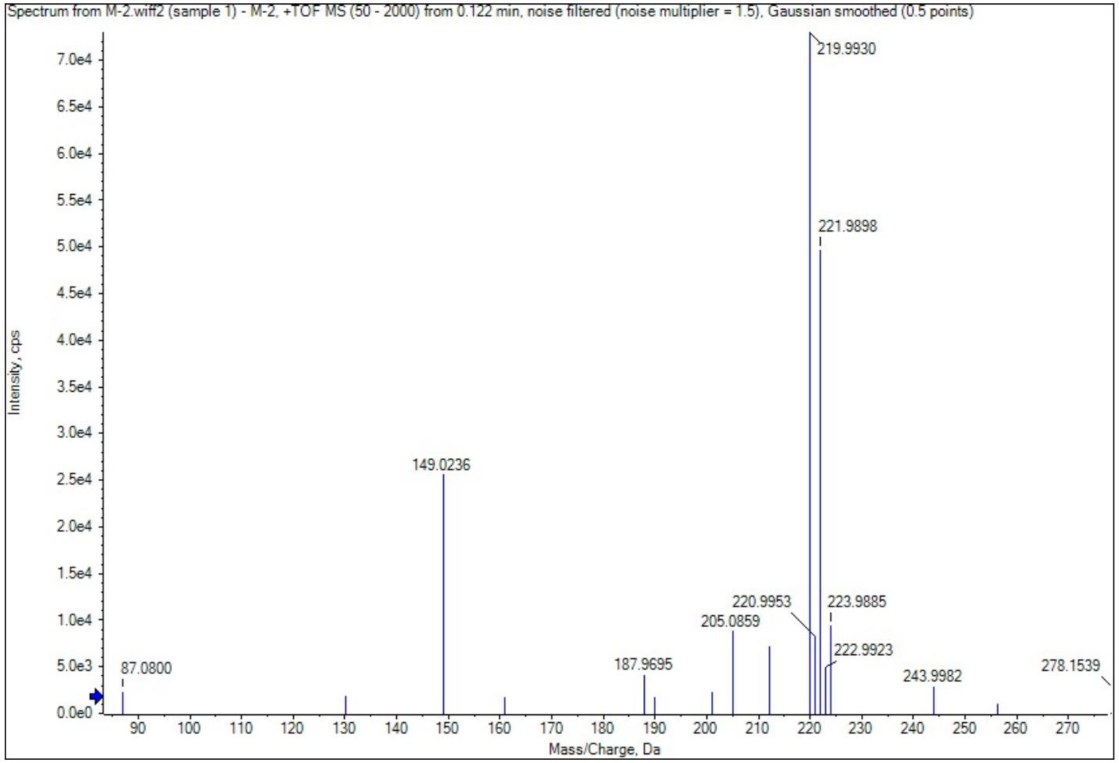

Page 1 of 1

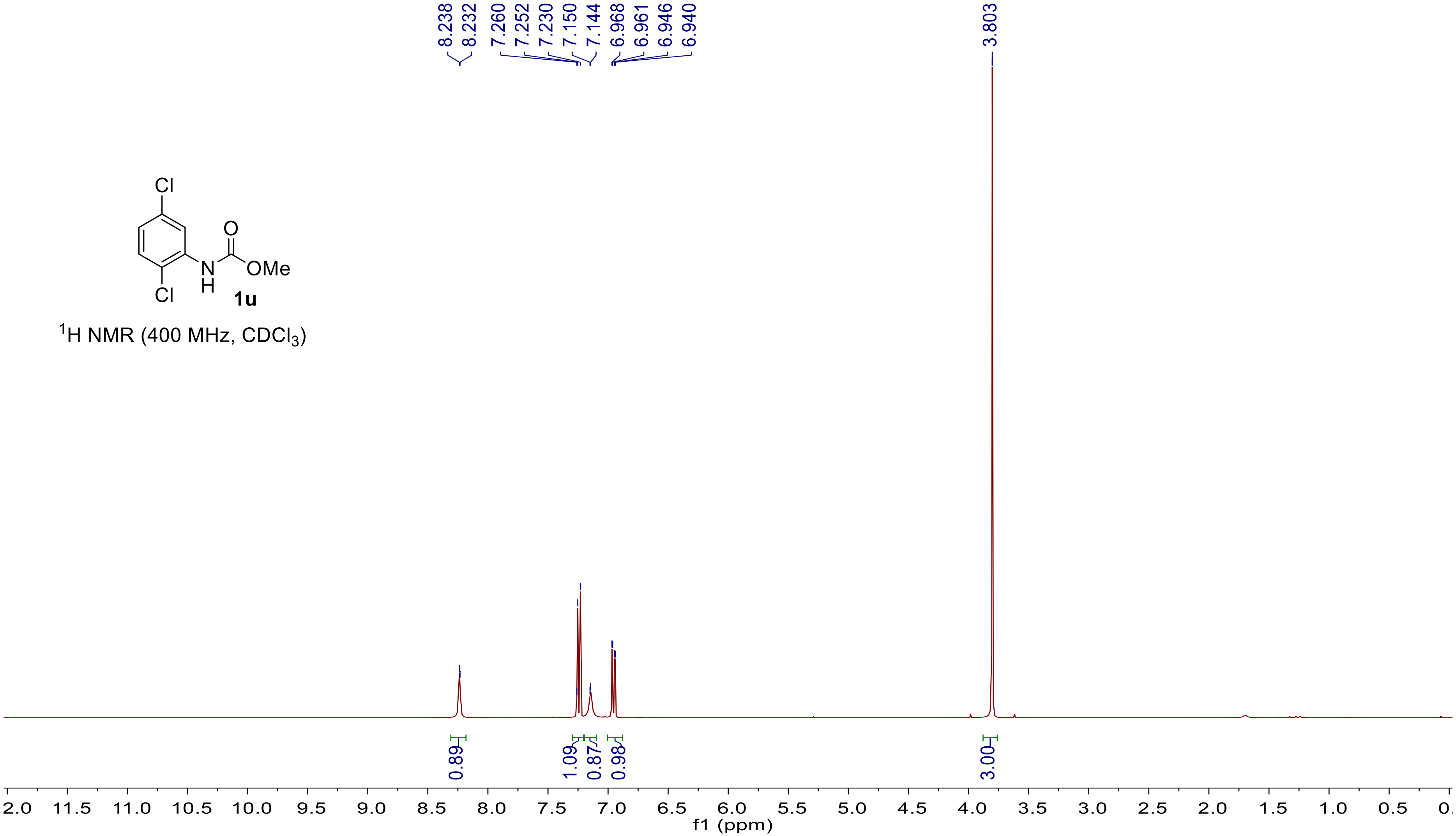
Cl
O
N
H
OMe
Cl
1u
1H NMR (400 MHz, CDCl3)
8.238
8.232
7.260
7.252
7.230
7.150
7.144
6.968
6.961
6.946
6.940
3.803
0.89
1.09
0.87
0.98
3.00
2.0 11.5 11.0 10.5 10.0 9.5 9.0 8.5 8.0 7.5 7.0 6.5 6.0 5.5 5.0 4.5 4.0 3.5 3.0 2.5 2.0 1.5 1.0 0.5 0.
f1 (ppm)

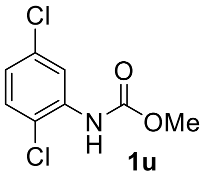

1u

^{13}C NMR (100 MHz, $CDCl_3$)

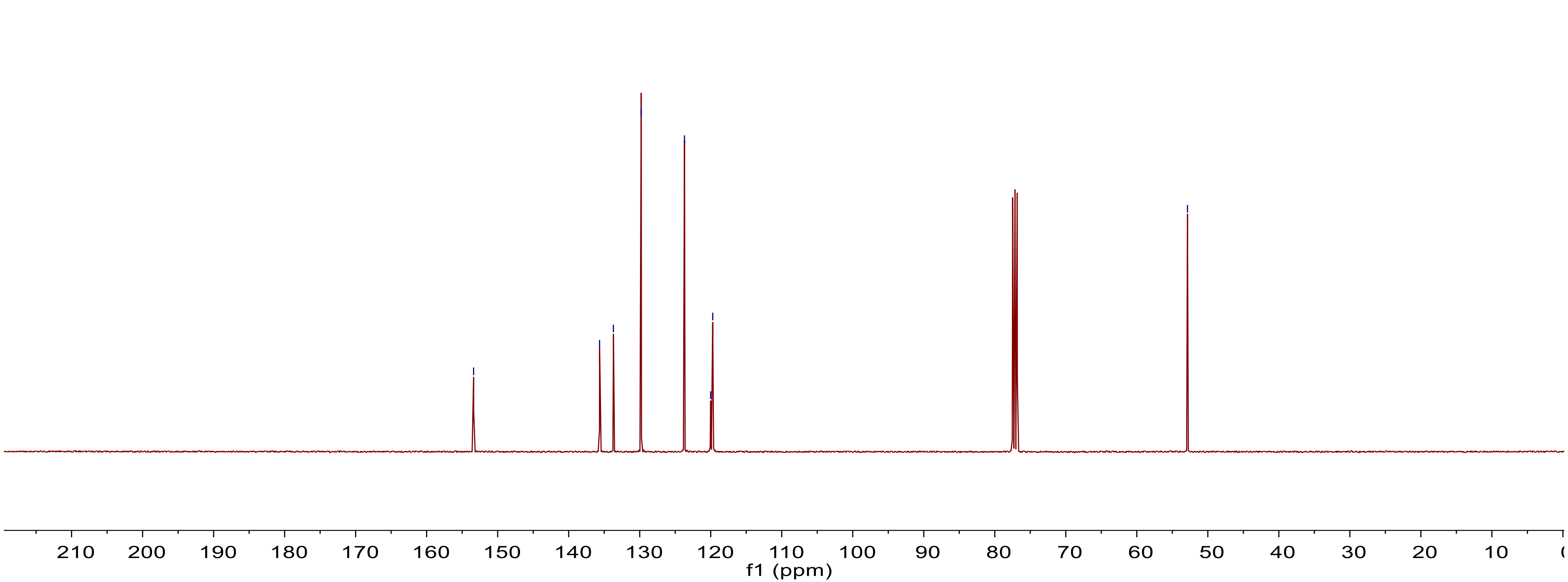

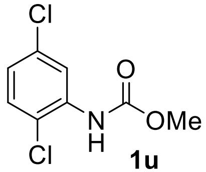

1u

HRMS (ESI)

SCIEX OS version: 3.0.0.3339
Workstation ID: DESKTOP-SI1BPI6

Printed by: DESKTOP-SI1BPI6/CZHG
Printed on: 7/13/2024 3:33:59 PM

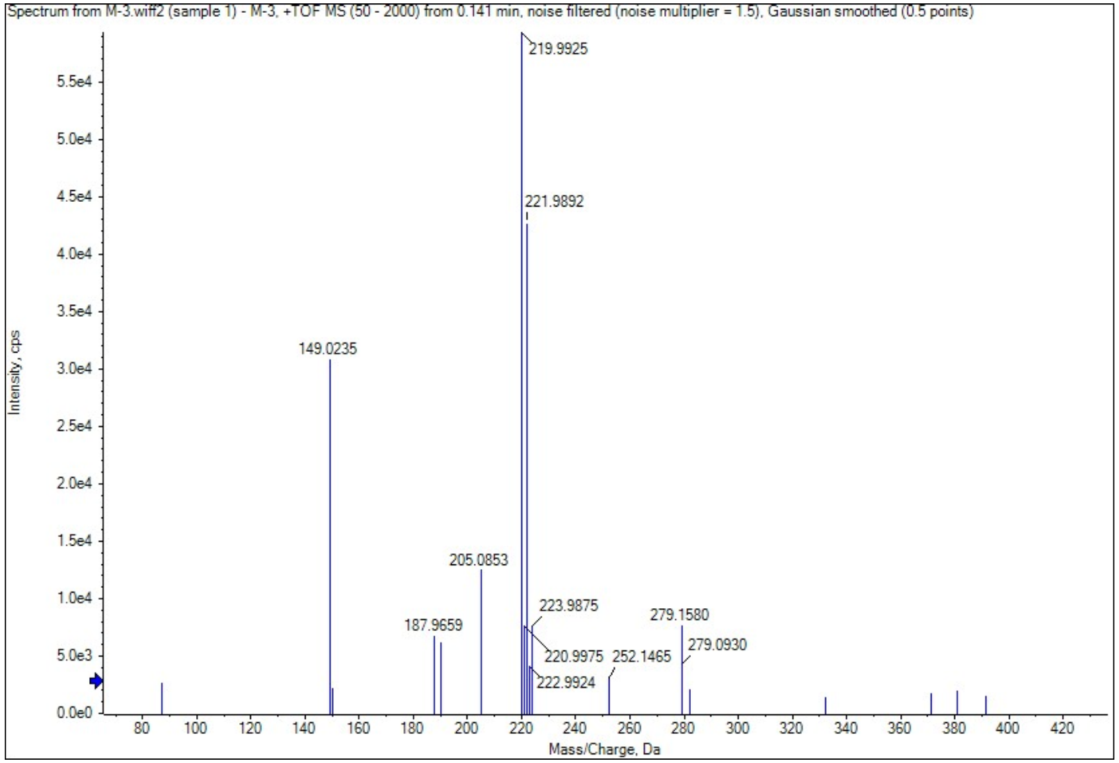

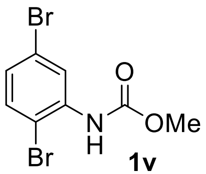

1v

^{1}H NMR (400 MHz, $CDCl_3$)

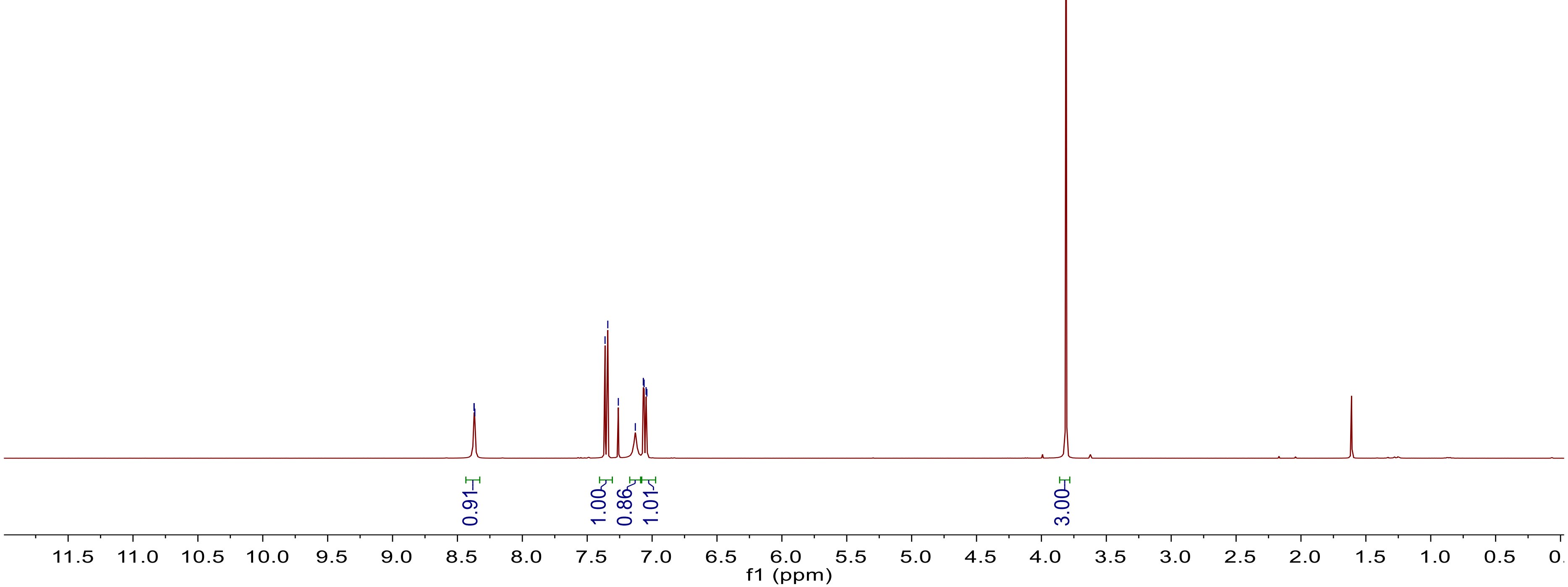

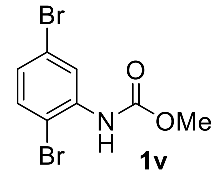

1v

^{13}C NMR (100 MHz, $CDCl_3$)

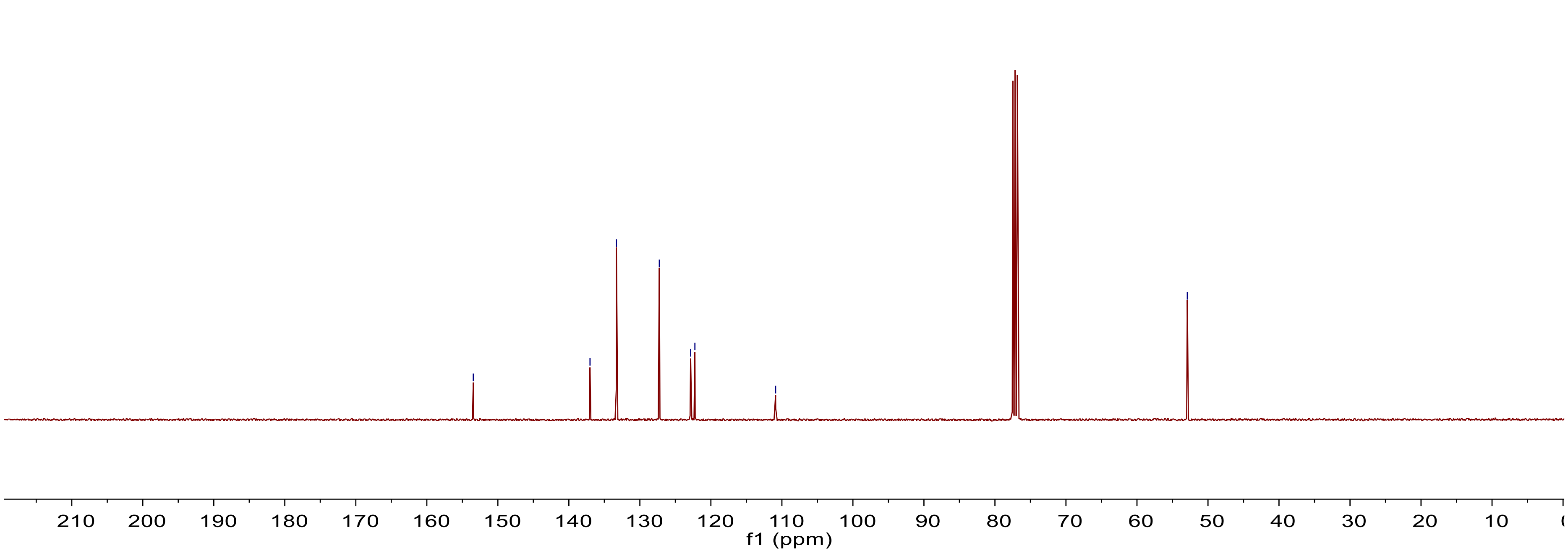

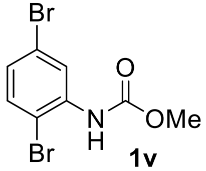

1v

HRMS (ESI)

SCIEX

SCIEX OS version: 3.0.0.3339
Workstation ID: DESKTOP-SI1BPI6

Printed by: DESKTOP-SI1BPI6/CZHG
Printed on: 7/13/2024 3:34:36 PM

Spectrum from M-4.wiff2 (sample 1) - M-4, +TOF MS (50 - 2000) from 0.124 min, noise filtered (noise multiplier = 1.5), Gaussian smoothed (0.5 points)

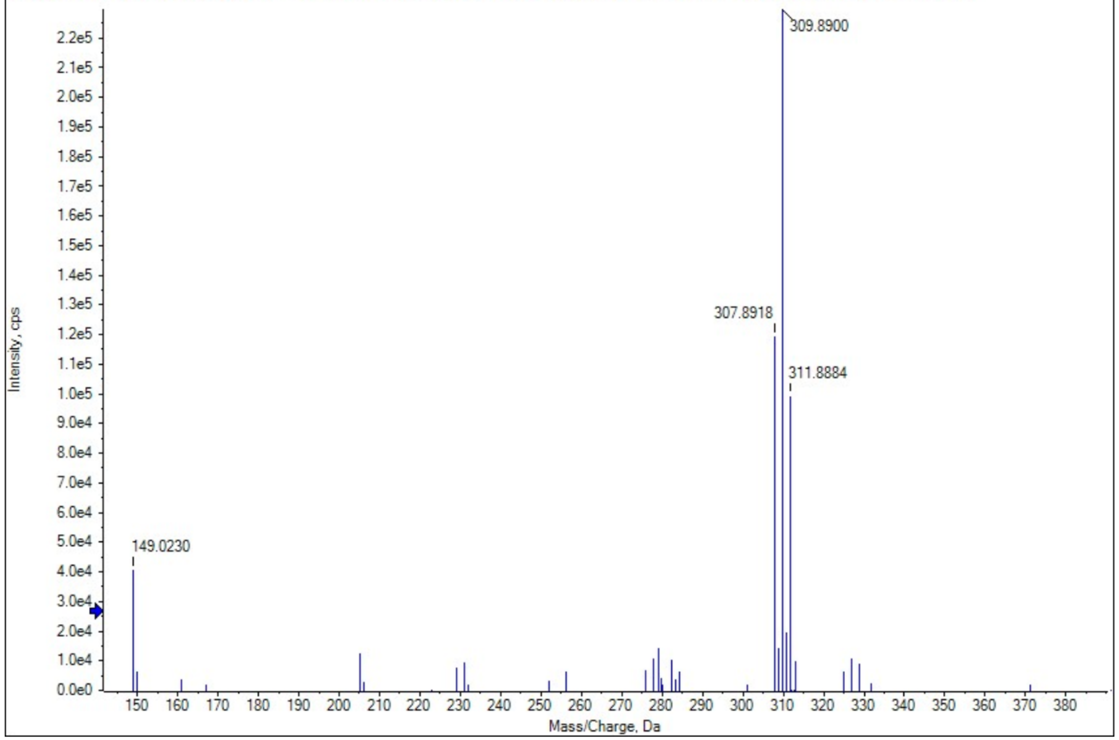

Page 1 of 1

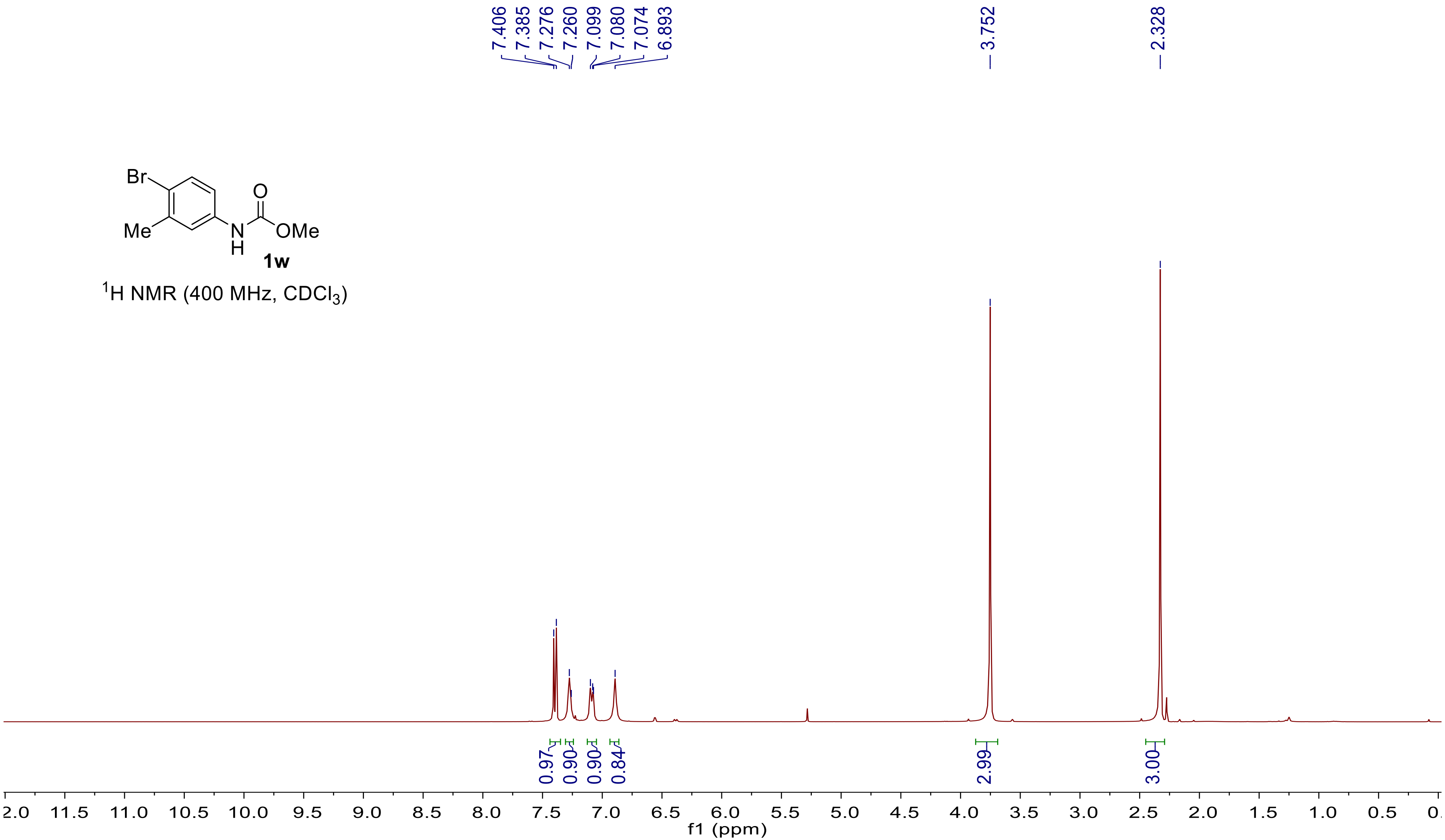

Br
Me
O
N
H
OMe
1w
^{1}H NMR (400 MHz, CDCl$_3$)
7.406
7.385
7.276
7.260
7.099
7.080
7.074
6.893
3.752
2.328
0.97
0.90
0.90
0.84
2.99
3.00
2.0
11.5
11.0
10.5
10.0
9.5
9.0
8.5
8.0
7.5
7.0
6.5
6.0
5.5
5.0
4.5
4.0
3.5
3.0
2.5
2.0
1.5
1.0
0.5
f1 (ppm)

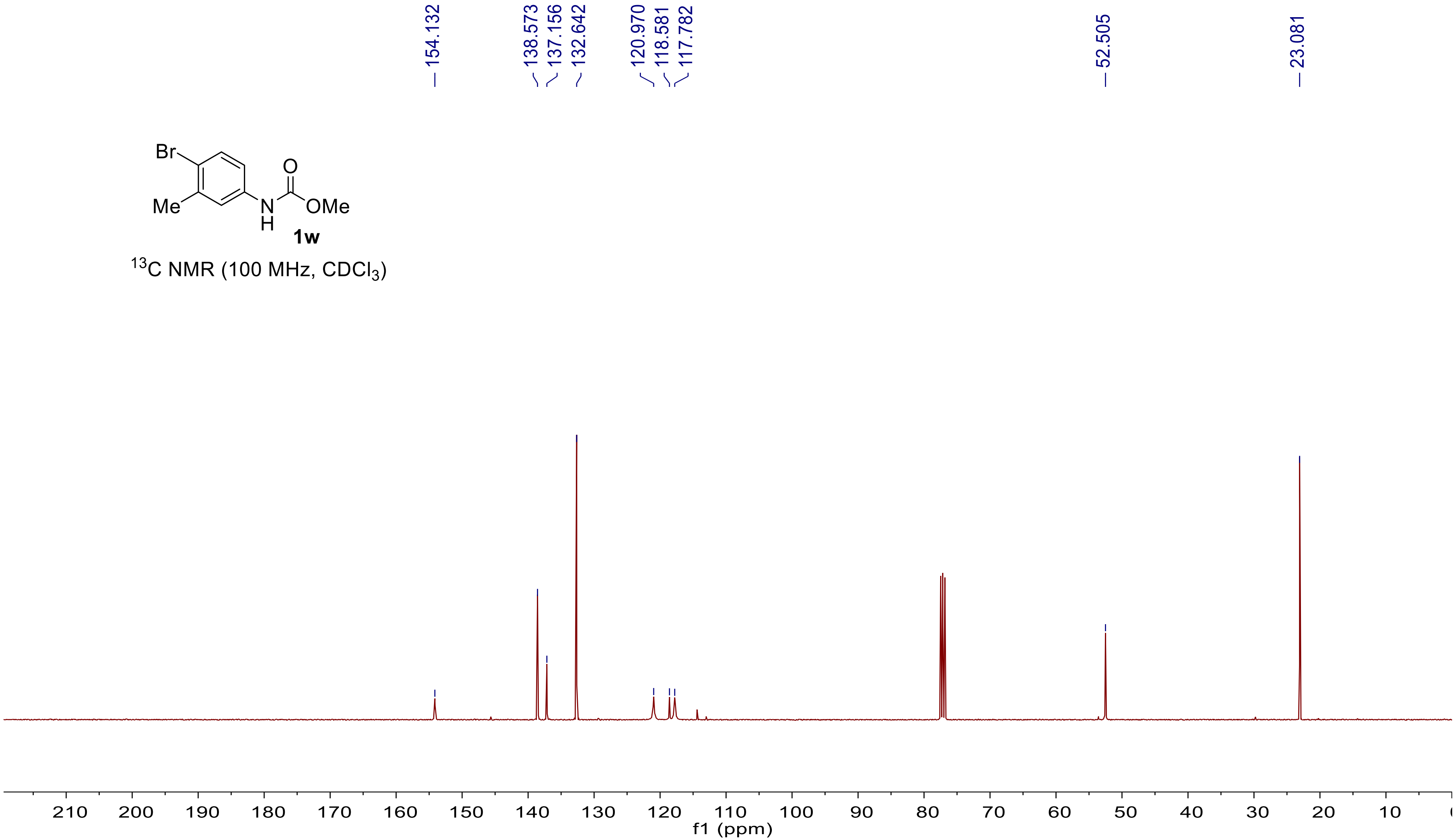
154.132
138.573
137.156
132.642
120.970
118.581
117.782
52.505
23.081
Br
Me
O
N
H
OMe
1w
^{13}C NMR (100 MHz, $CDCl_3$)
210 200 190 180 170 160 150 140 130 120 110 100 90 80 70 60 50 40 30 20 10
f1 (ppm)

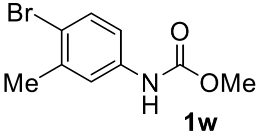

1w

HRMS (ESI)

SCIEX OS version: 3.0.0.3339
Workstation ID: DESKTOP-SI1BPI6

Printed by: DESKTOP-SI1BPI6/CZHG
Printed on: 7/13/2024 3:35:04 PM

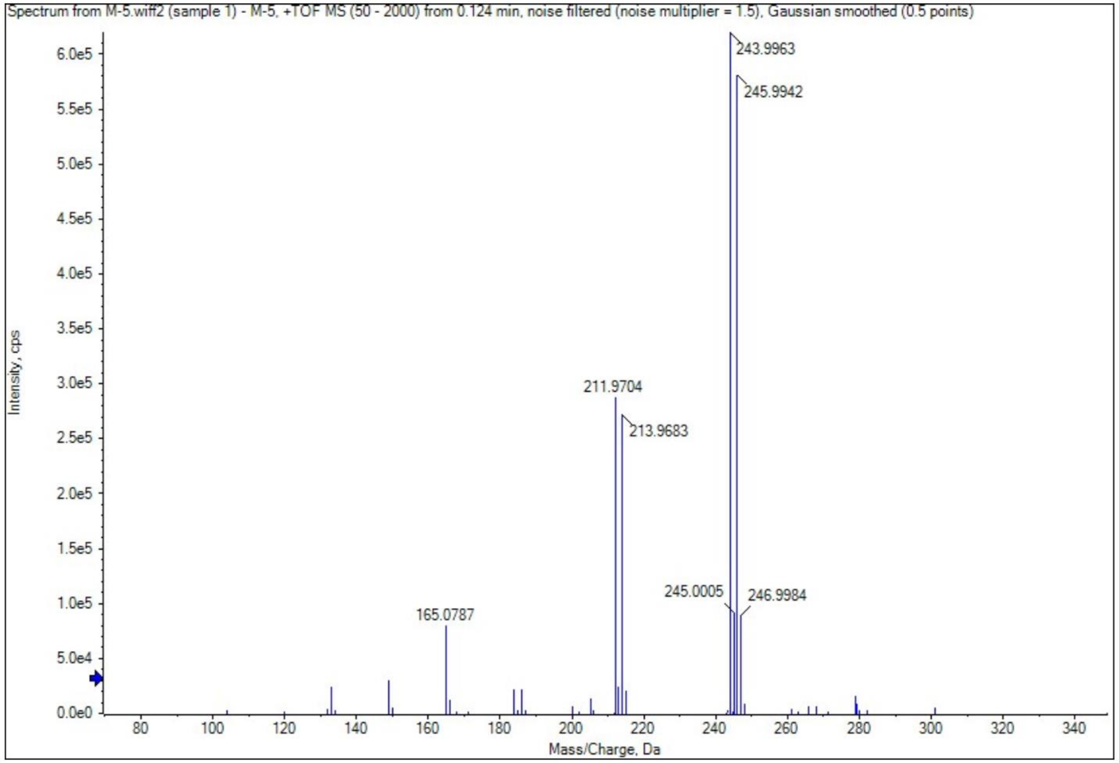

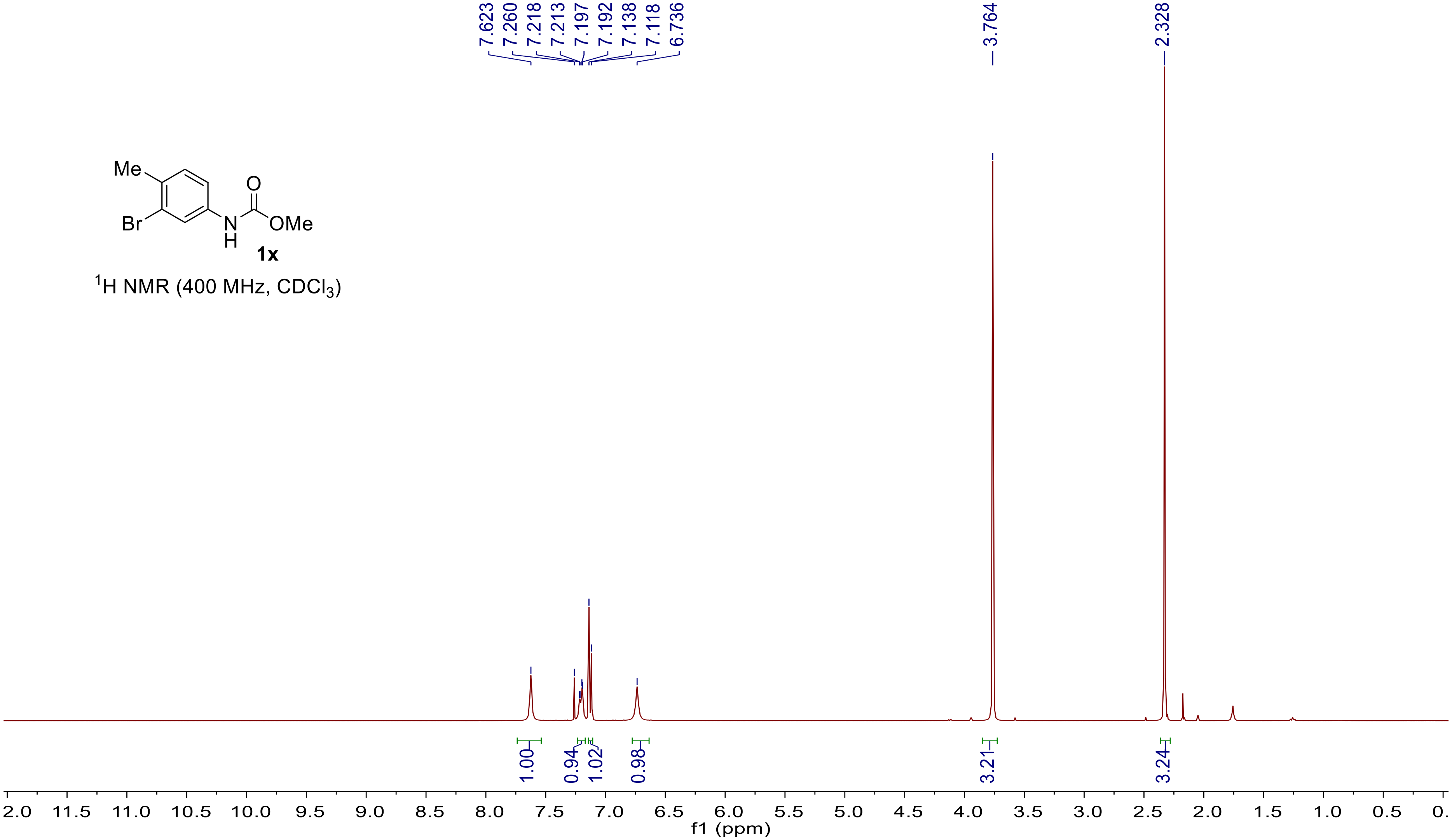

1x

^{1}H NMR (400 MHz, $CDCl_3$)

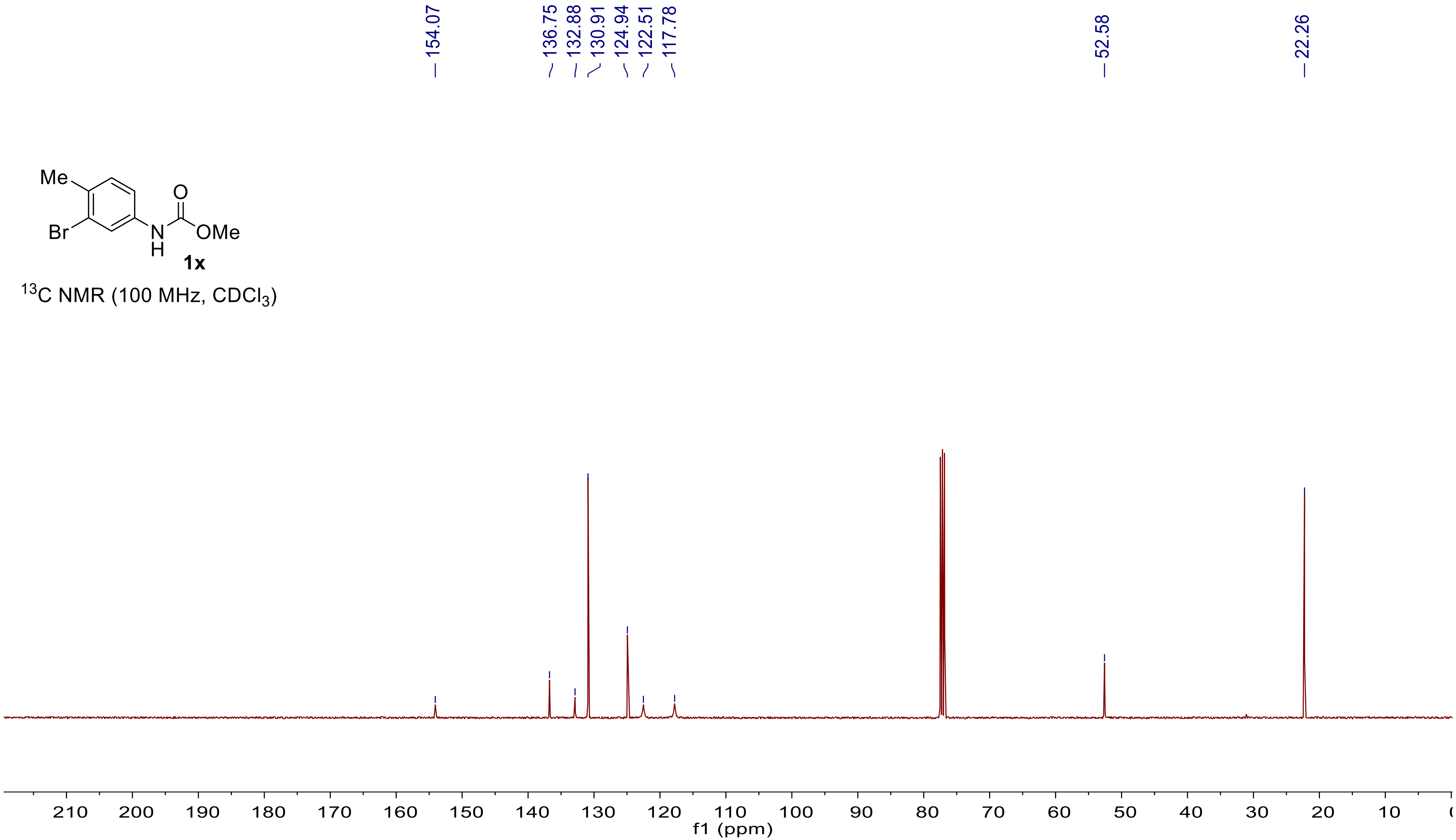

1x

^{13}C NMR (100 MHz, $CDCl_3$)

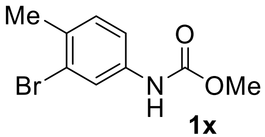

1x

HRMS (ESI)

SCIEX OS version: 3.0.0.3339 **Printed by:** DESKTOP-SI1BPI6/CZHG

Workstation ID: DESKTOP-SI1BPI6 **Printed on:** 7/13/2024 3:35:39 PM

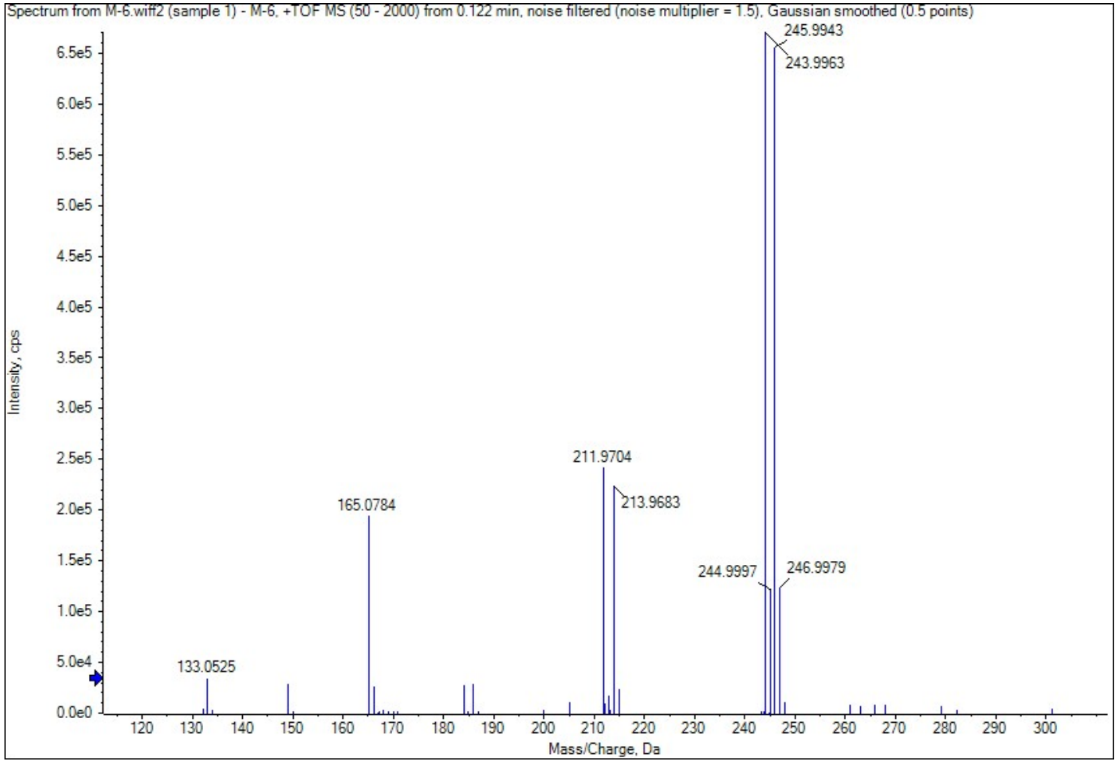

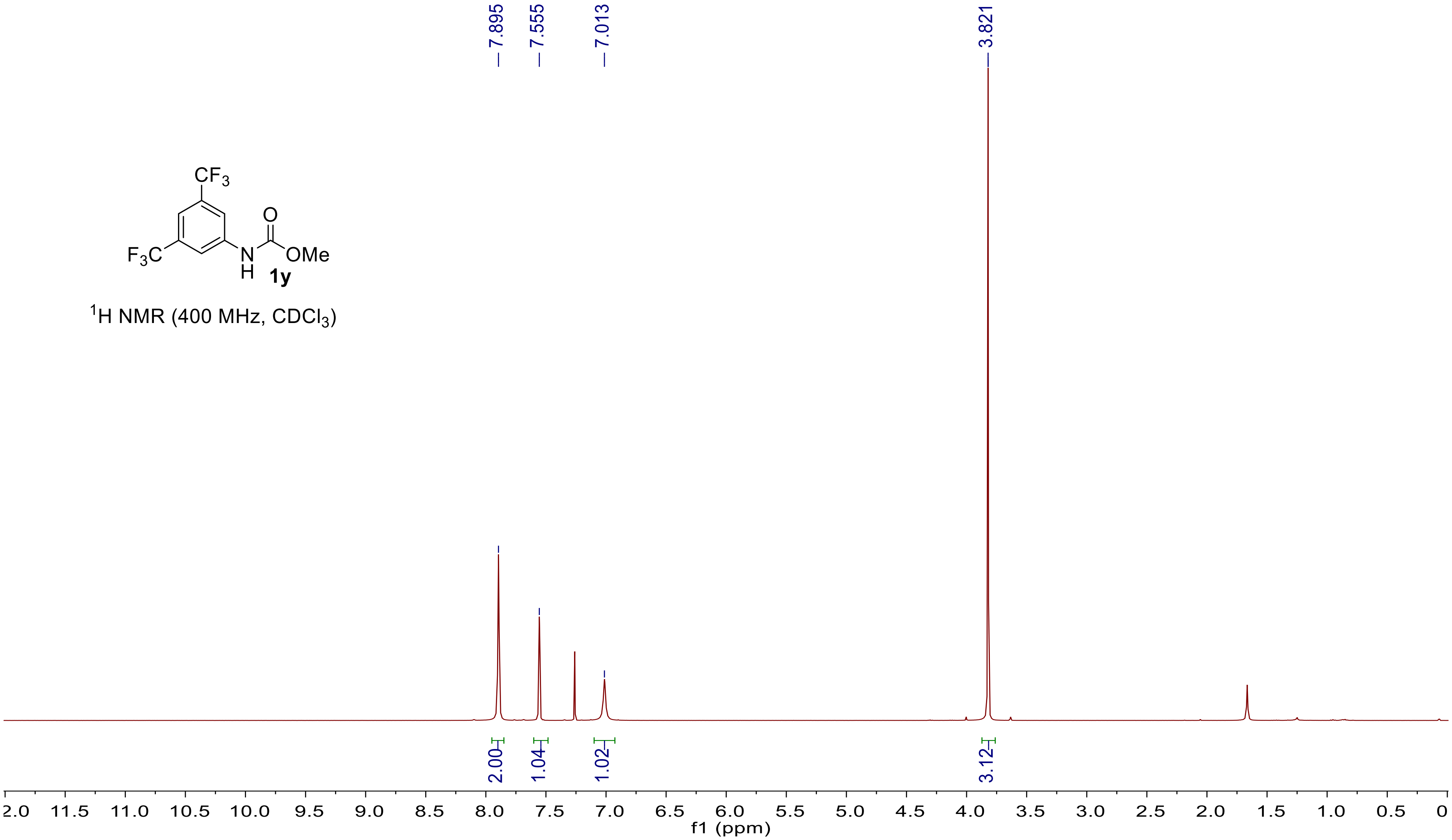

1y

^{1}H NMR (400 MHz, $CDCl_3$)

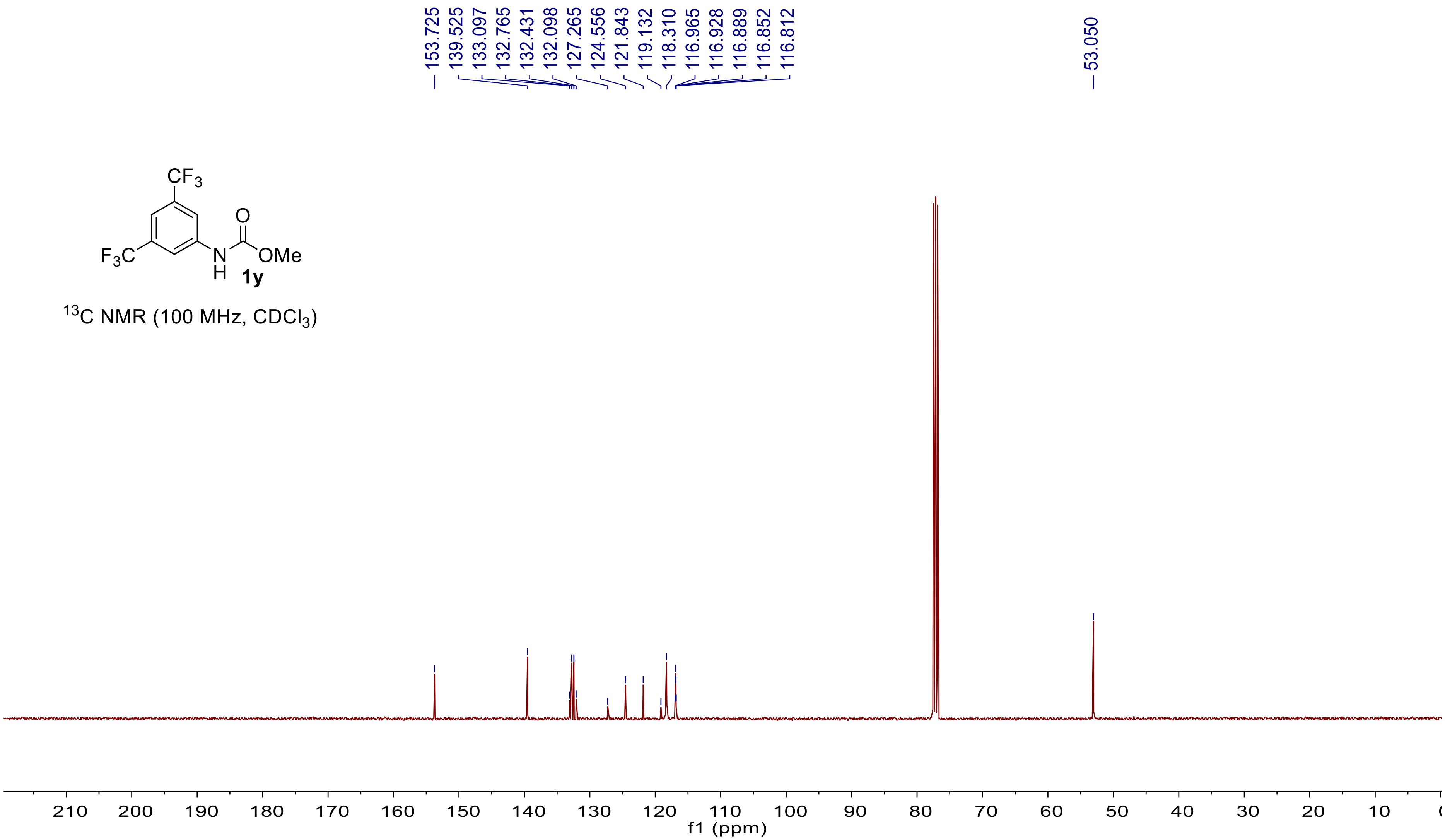

153.725
139.525
133.097
132.765
132.431
132.098
127.265
124.556
121.843
119.132
118.310
116.965
116.928
116.889
116.852
116.812
53.050
CF3
O
F3C
N
H
OMe
1y
13C NMR (100 MHz, CDCl3)
210
200
190
180
170
160
150
140
130
120
110
100
90
80
70
60
50
40
30
20
10
f1 (ppm)

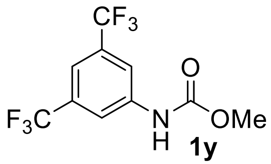

1y

^{19}F NMR (376 MHz, $CDCl_3$)

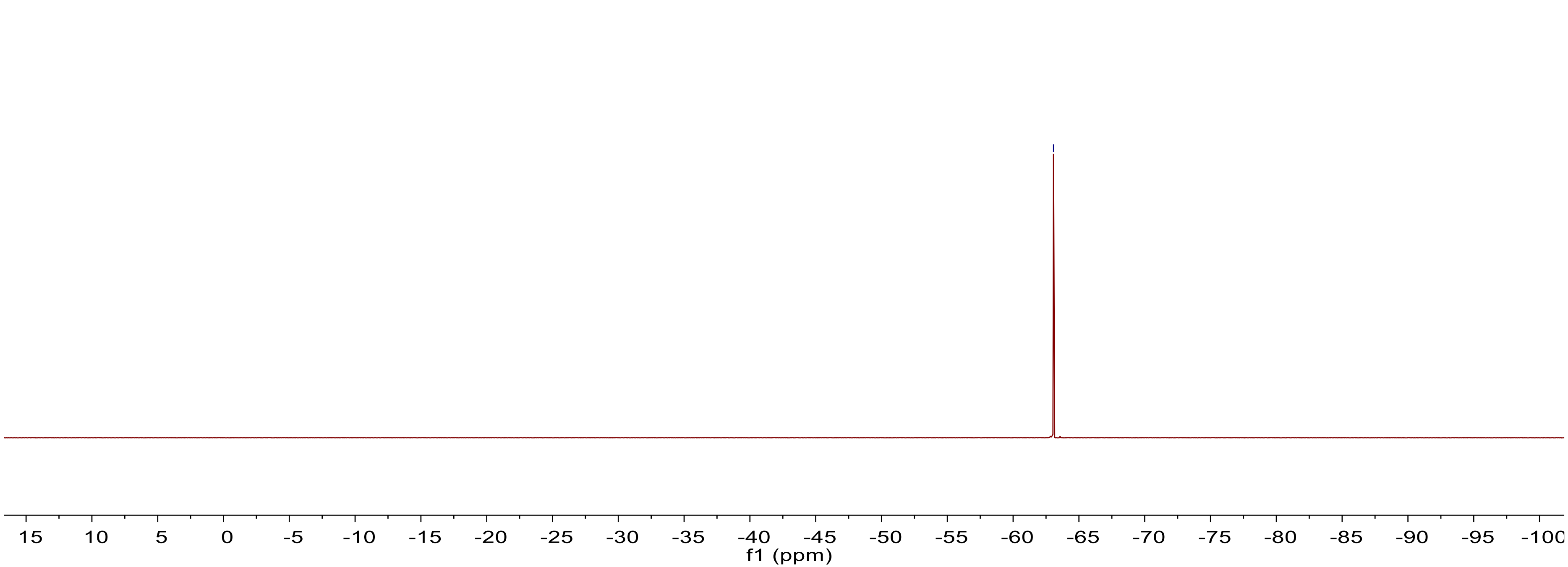

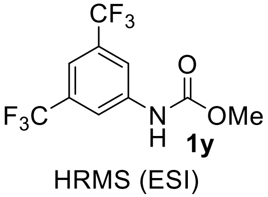

1y

HRMS (ESI)

SCIEX OS version: 3.0.0.3339 **Printed by:** DESKTOP-SI1BPI6/CZHG
Workstation ID: DESKTOP-SI1BPI6 **Printed on:** 7/13/2024 3:53:53 PM

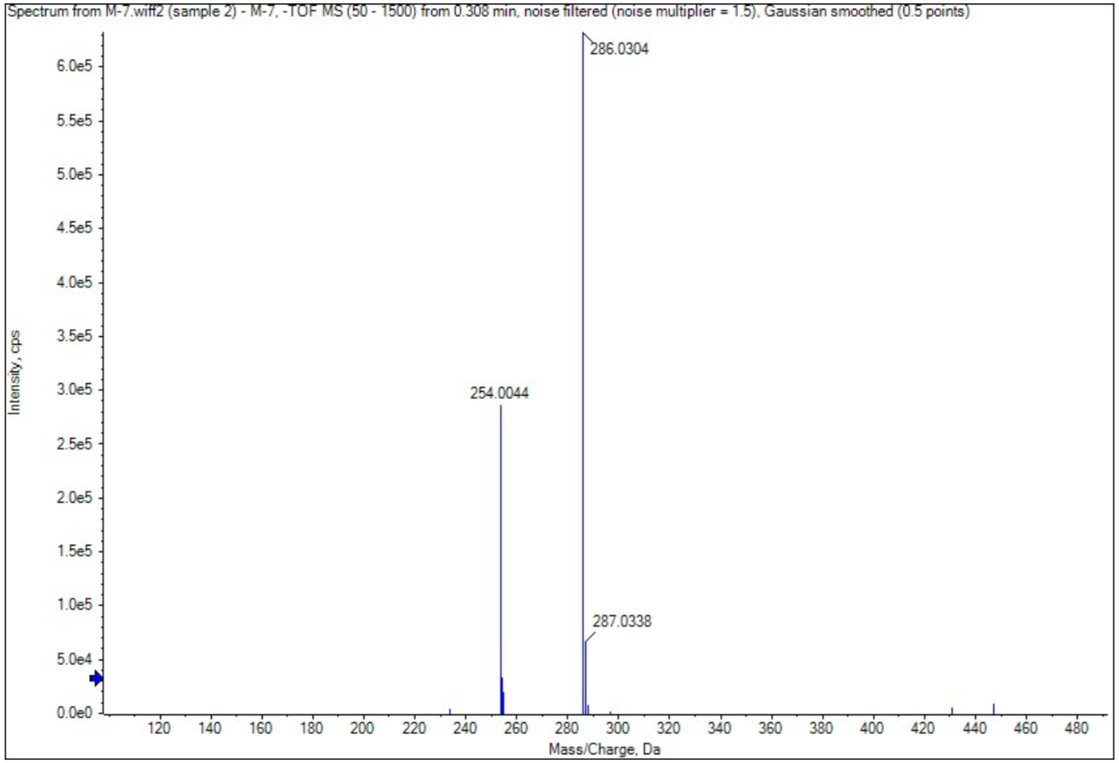

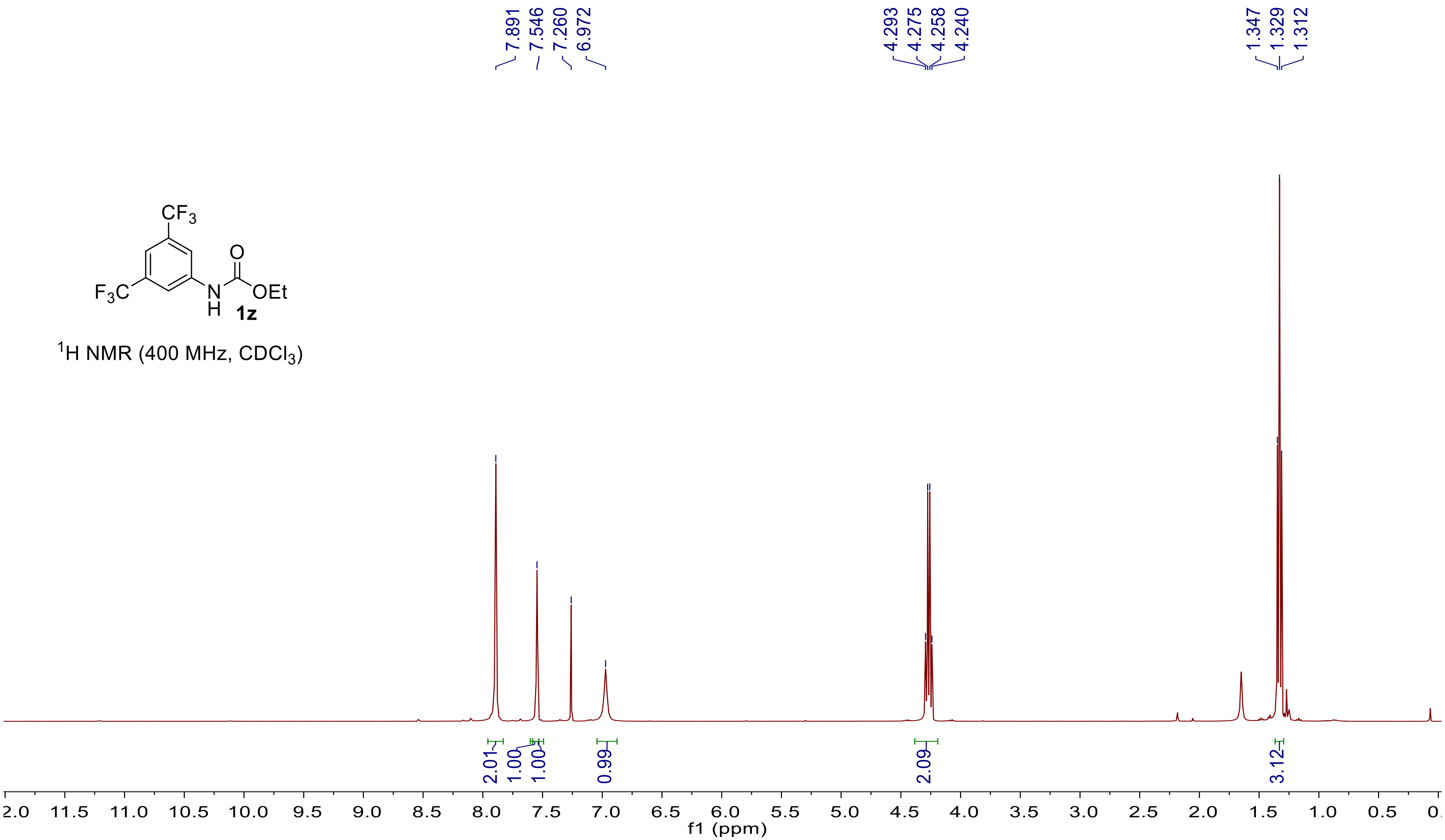
7.891
7.546
7.260
6.972
4.293
4.275
4.258
4.240
1.347
1.329
1.312
CF3
F3C
N
H
O
OEt
1z
1H NMR (400 MHz, CDCl3)
2.01
1.00
1.00
0.99
2.09
3.12
f1 (ppm)

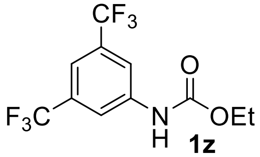

^{13}C NMR (100 MHz, $CDCl_3$)

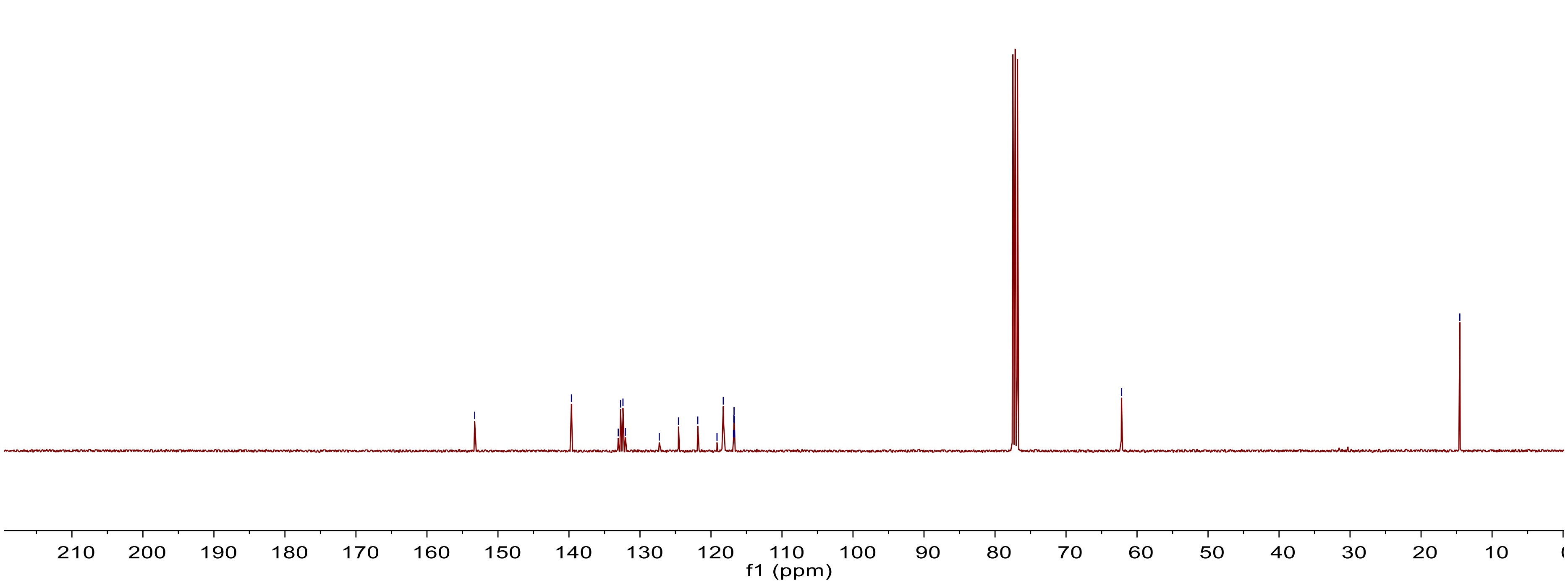

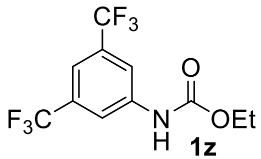

1z

^{19}F NMR (376 MHz, $CDCl_3$)

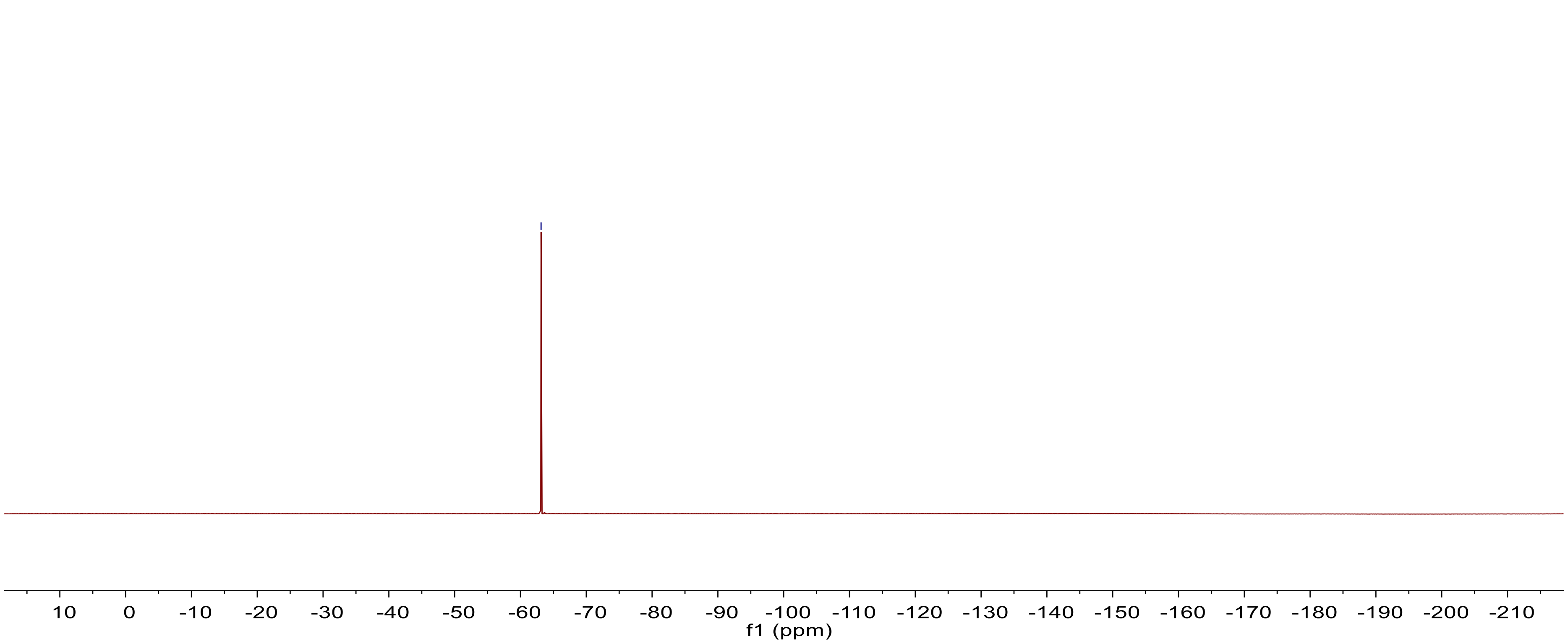

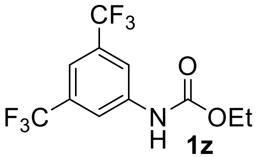

1z

HRMS (ESI)

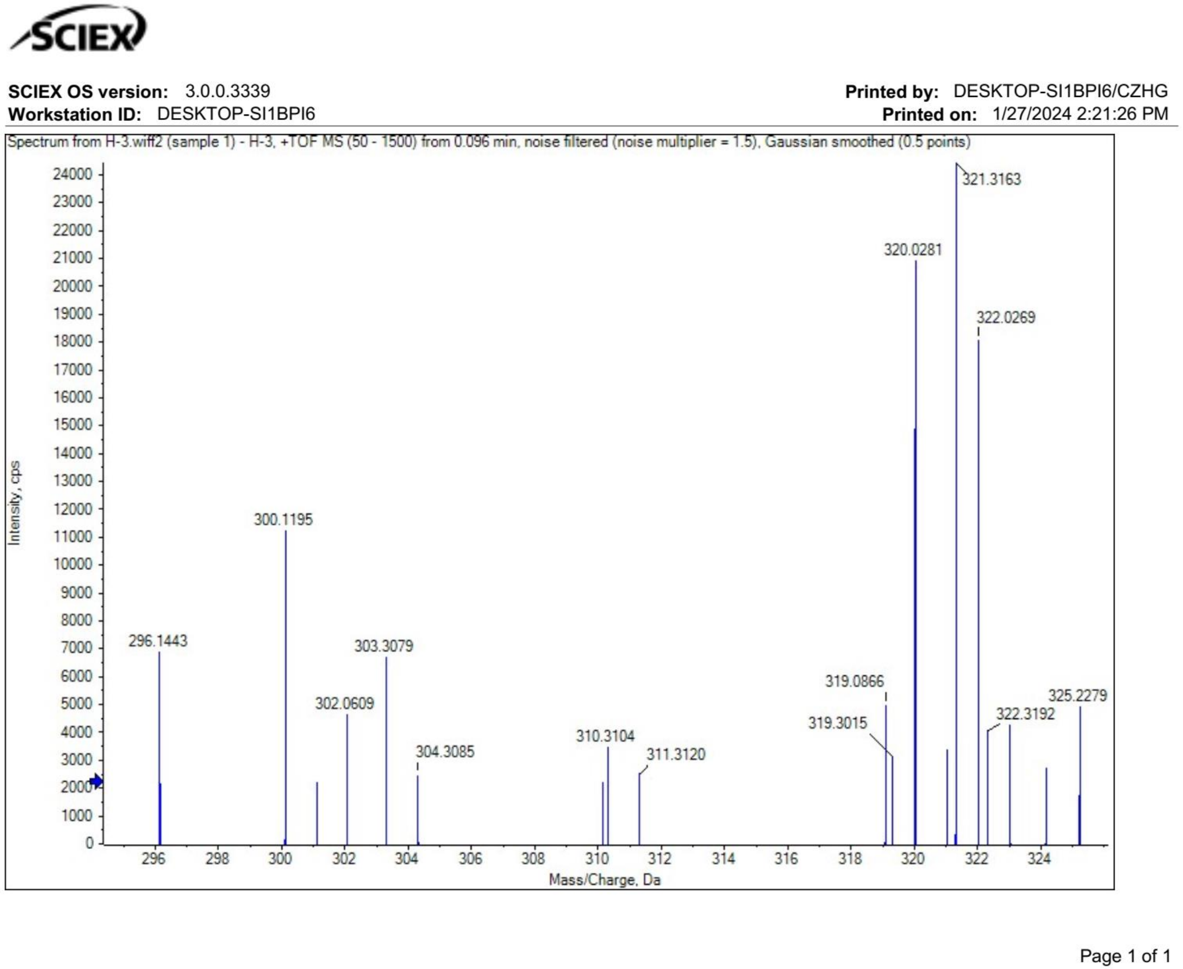
SCIEX
SCIEX OS version: 3.0.0.3339
Workstation ID: DESKTOP-SI1BPI6
Printed by: DESKTOP-SI1BPI6/CZHG
Printed on: 1/27/2024 2:21:26 PM
Spectrum from H-3.wiff2 (sample 1) - H-3, +TOF MS (50 - 1500) from 0.096 min, noise filtered (noise multiplier = 1.5), Gaussian smoothed (0.5 points)
Intensity, cps
Mass/Charge, Da
321.3163
320.0281
322.0269
300.1195
296.1443
303.3079
302.0609
304.3085
310.3104
311.3120
319.0866
319.3015
322.3192
325.2279
Page 1 of 1

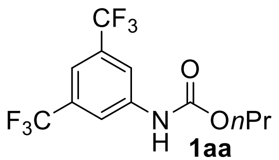

1aa

^{1}H NMR (400 MHz, $CDCl_3$)

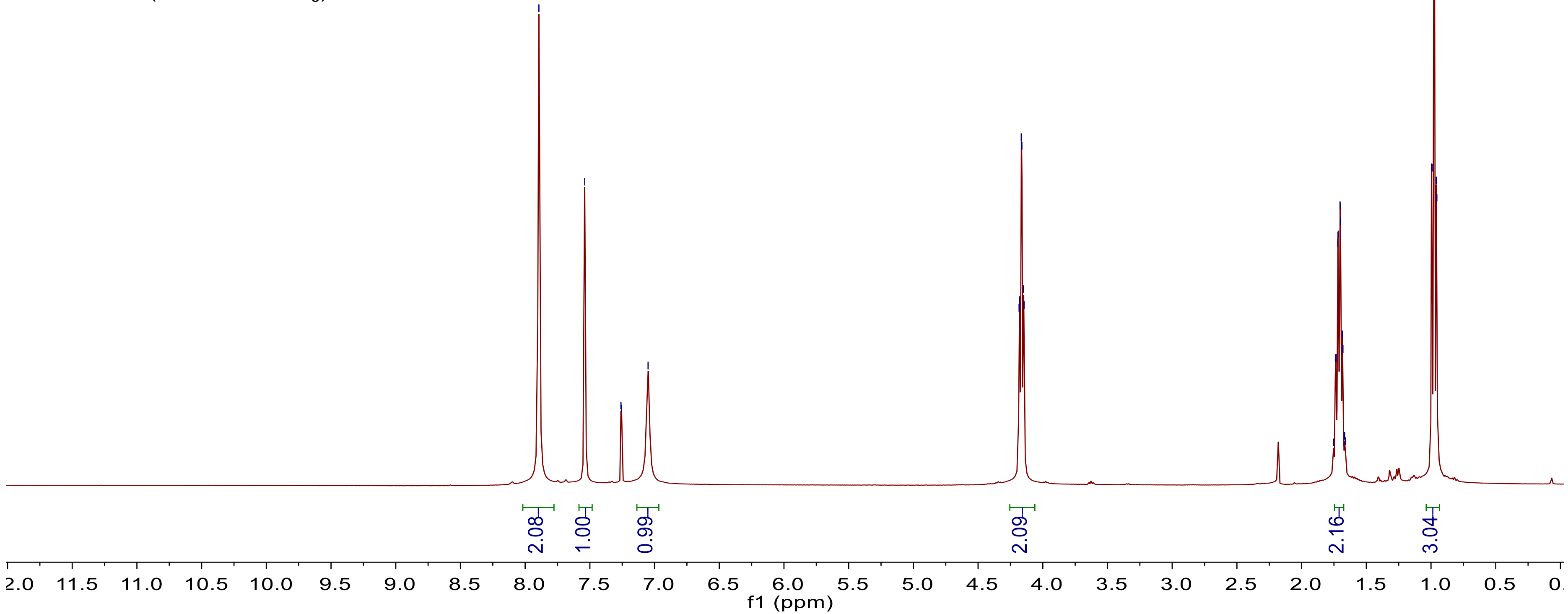

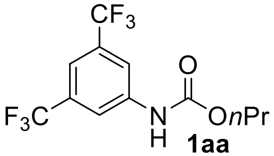

1aa

^{13}C NMR (100 MHz, $CDCl_3$)

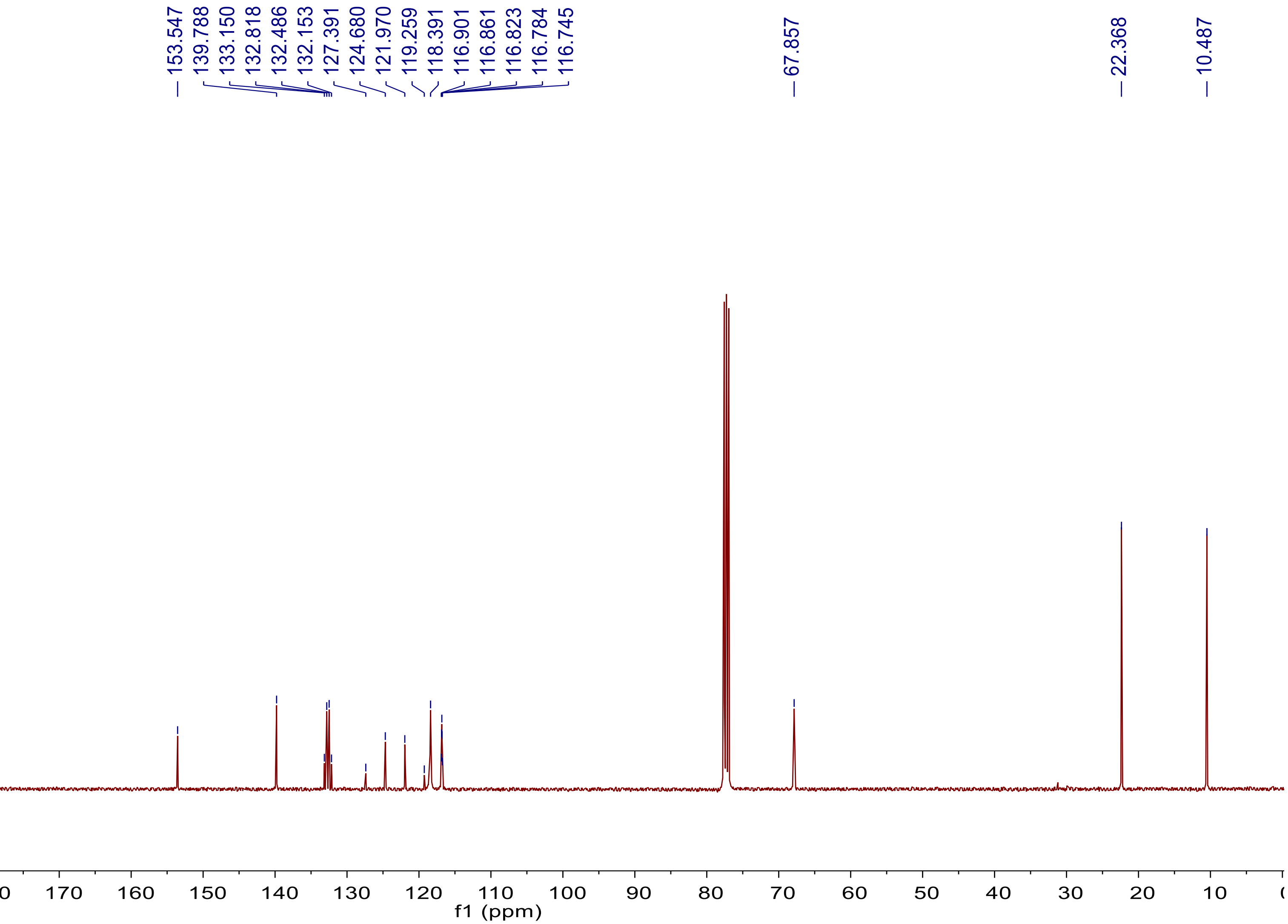

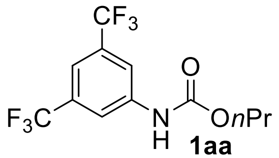

1aa

^{19}F NMR (376 MHz, $CDCl_3$)

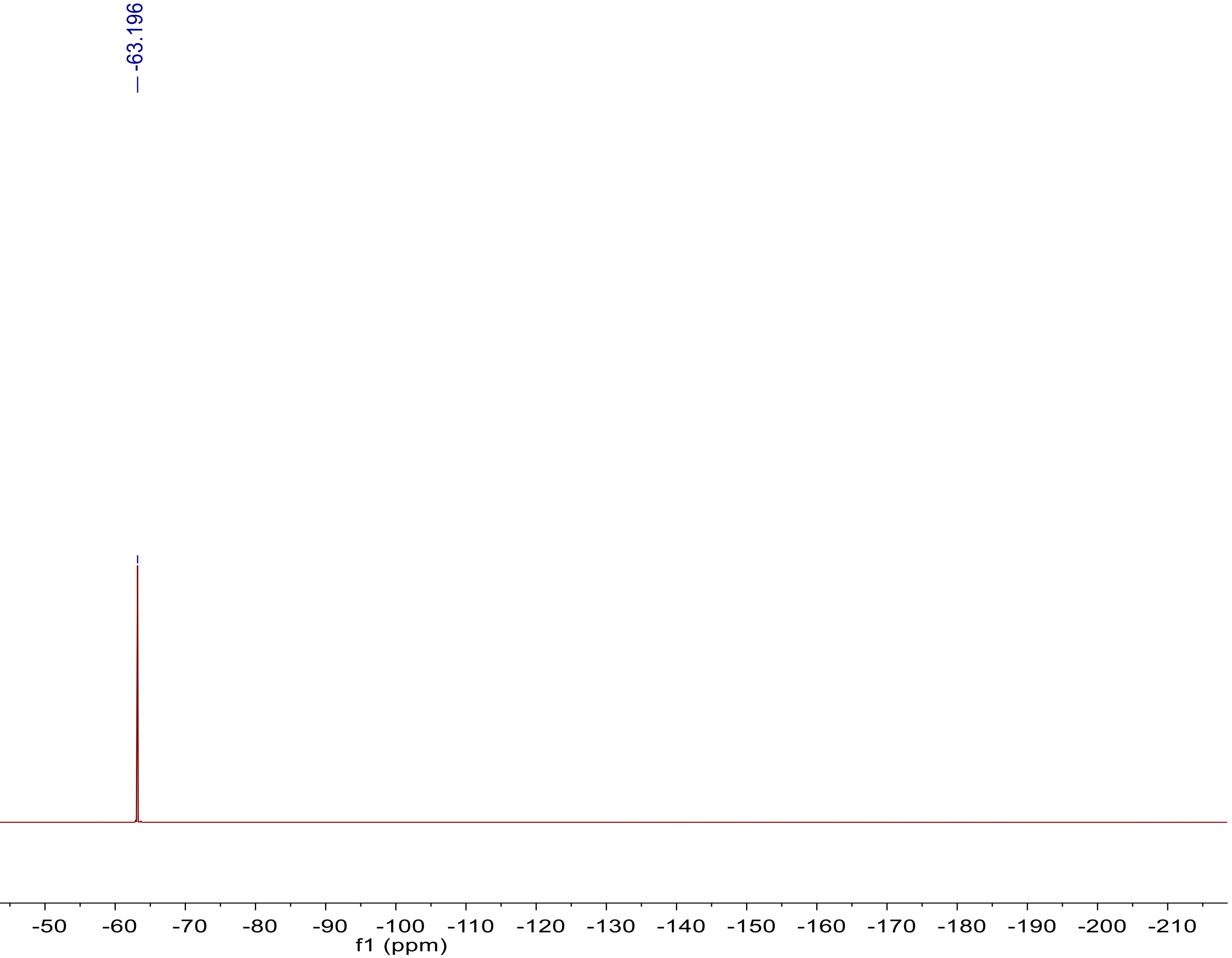

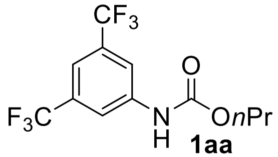

1aa

HRMS (ESI)

SCIEX OS version: 3.0.0.3339
Workstation ID: DESKTOP-SI1BPI6

Printed by: DESKTOP-SI1BPI6/CZHG
Printed on: 1/27/2024 2:22:06 PM

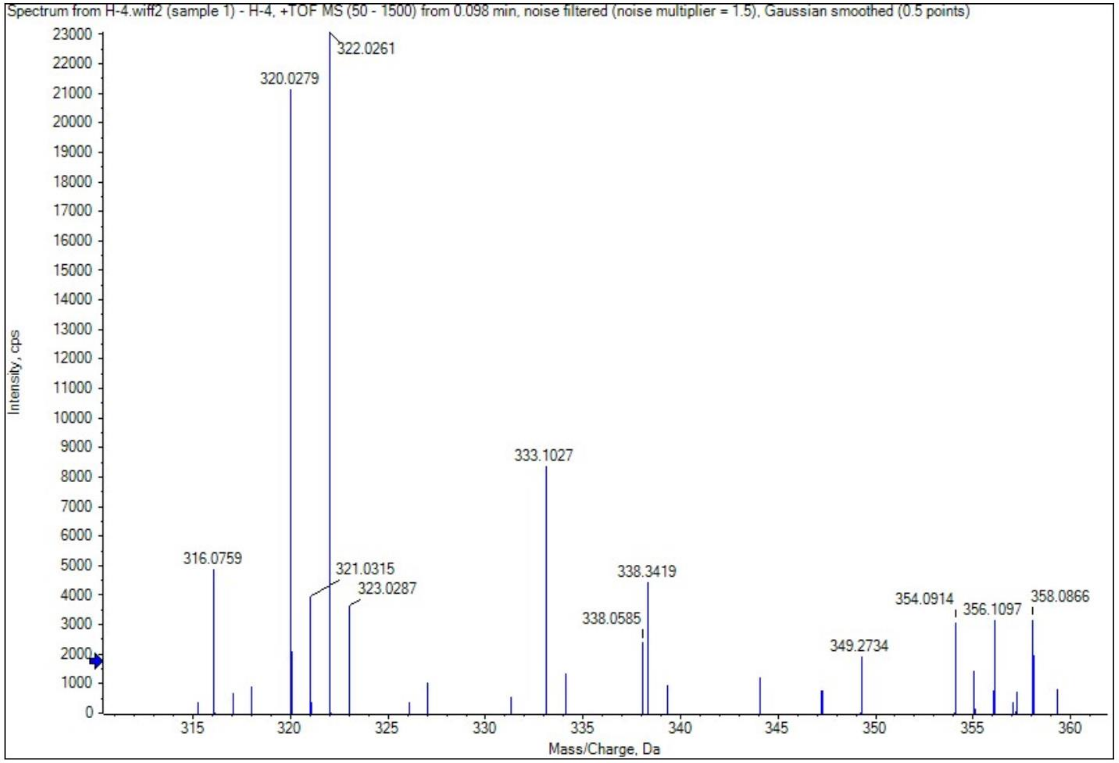

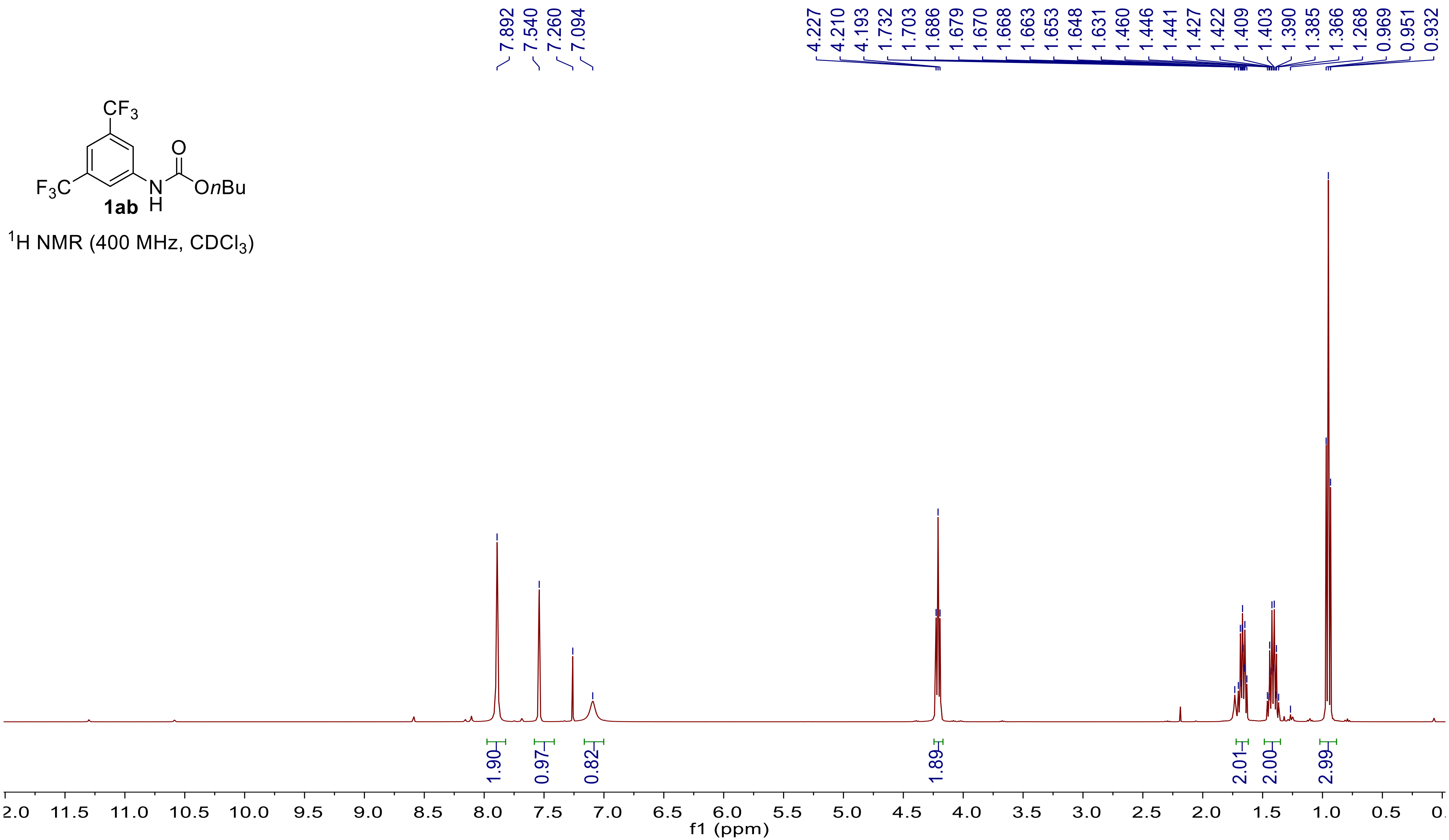

CF3
O
F3C
N
H
OnBu
1ab
1H NMR (400 MHz, CDCl3)
7.892
7.540
7.260
7.094
4.227
4.210
4.193
1.732
1.703
1.686
1.679
1.670
1.668
1.663
1.653
1.648
1.631
1.460
1.446
1.441
1.427
1.422
1.409
1.403
1.390
1.385
1.366
1.268
0.969
0.951
0.932
1.90
0.97
0.82
1.89
2.01
2.00
2.99
f1 (ppm)

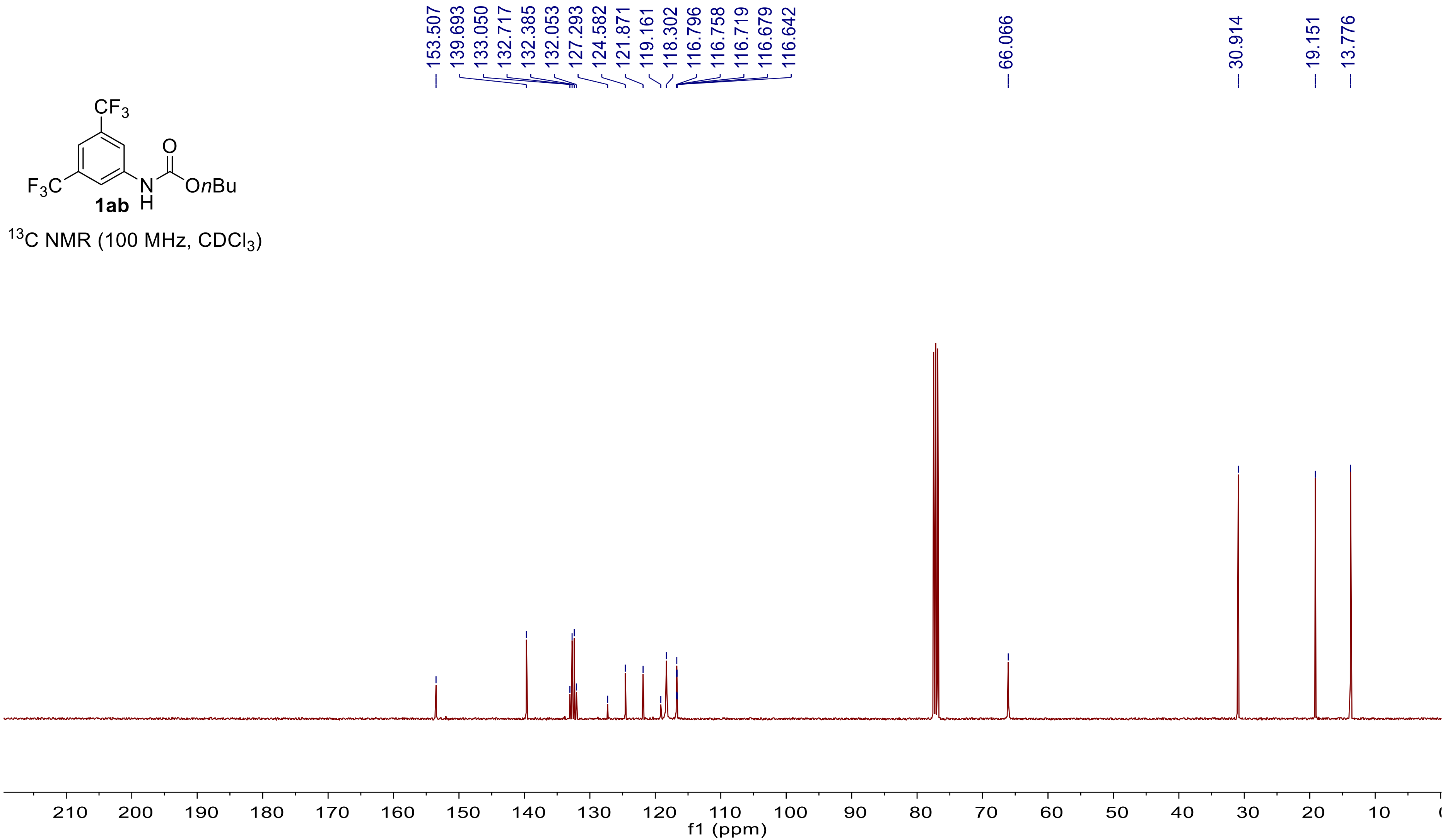

153.507
139.693
133.050
132.717
132.385
132.053
127.293
124.582
121.871
119.161
118.302
116.796
116.758
116.719
116.679
116.642
66.066
30.914
19.151
13.776
CF3
O
F3C
N
OnBu
1ab
H
13C NMR (100 MHz, CDCl3)
210
200
190
180
170
160
150
140
130
120
110
100
90
80
70
60
50
40
30
20
10
f1 (ppm)

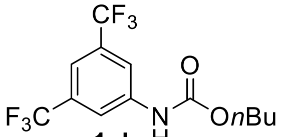

1ab

^{19}F NMR (376 MHz, $CDCl_3$)

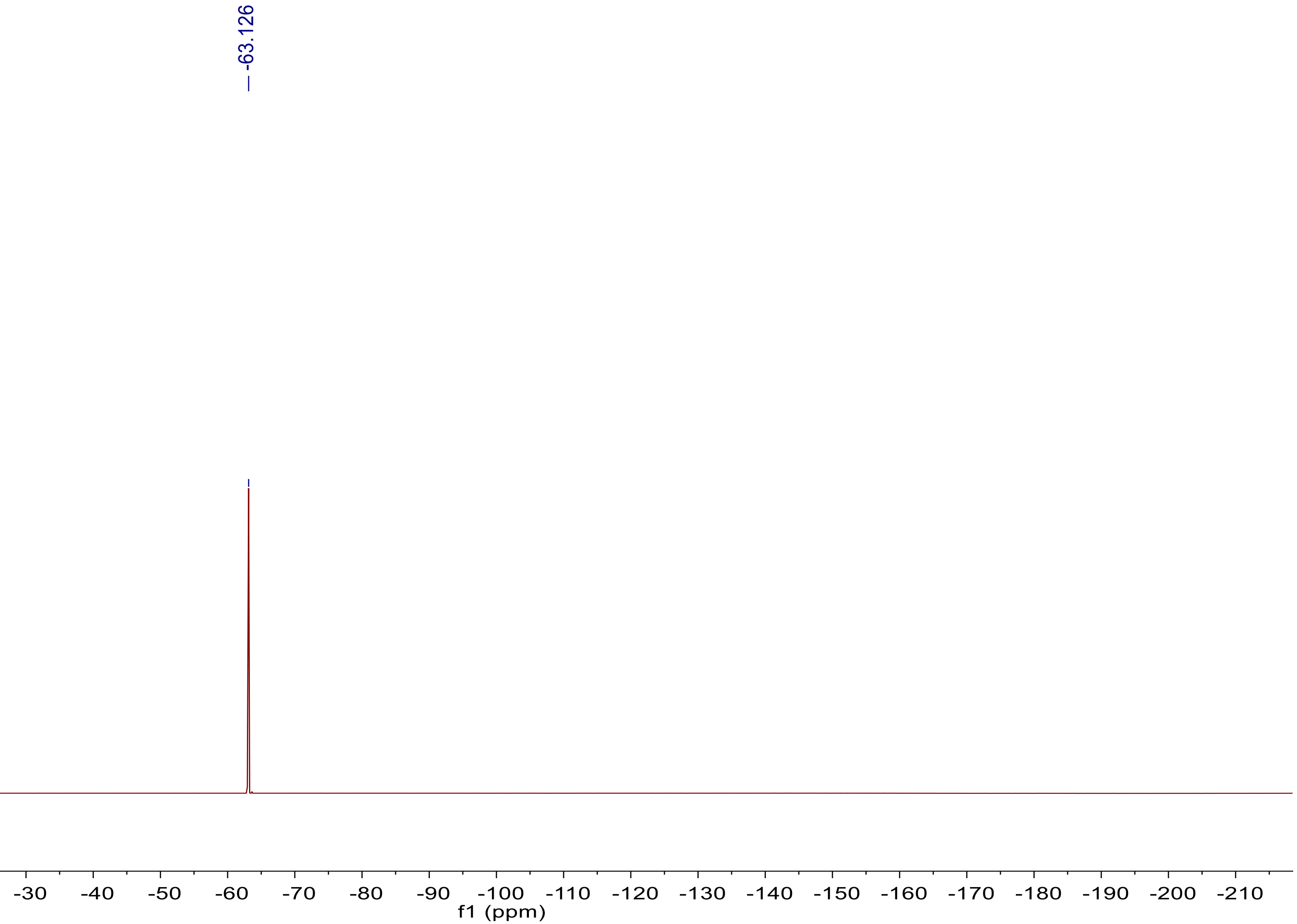

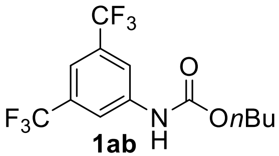

1ab

HRMS (ESI)

SCIEX OS version: 3.0.0.3339 **Printed by:** DESKTOP-SI1BPI6/CZHG

Workstation ID: DESKTOP-SI1BPI6 **Printed on:** 1/27/2024 2:22:31 PM

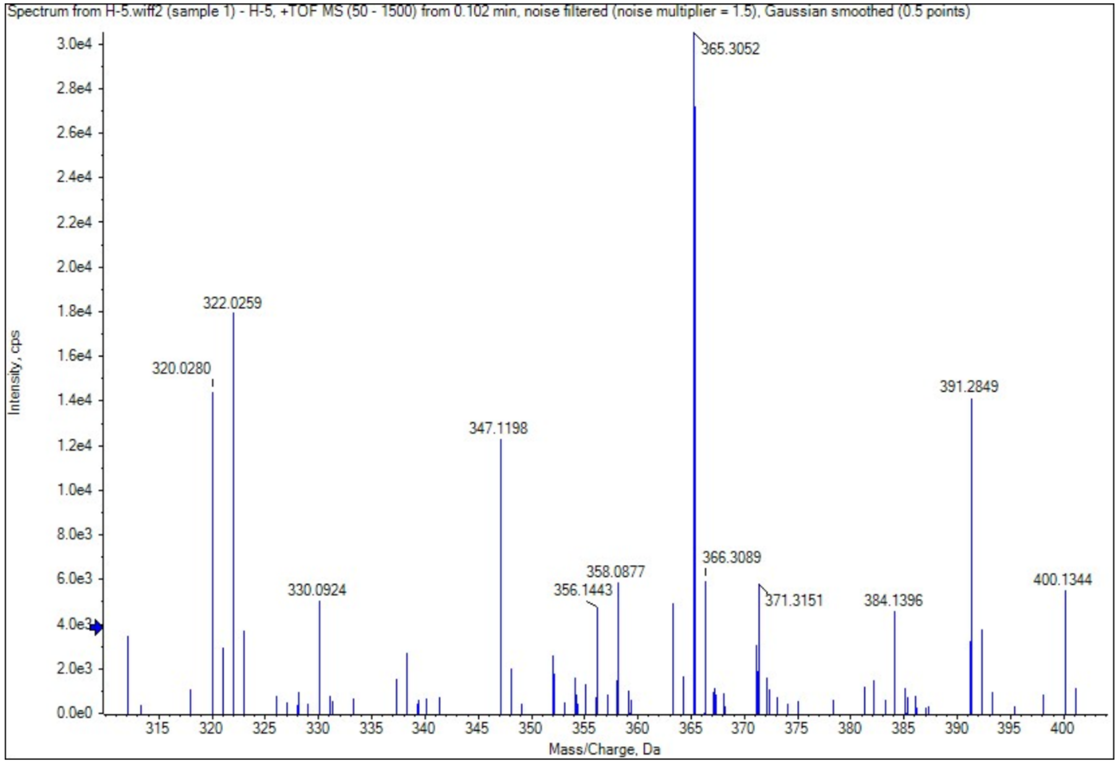

Page 1 of 1

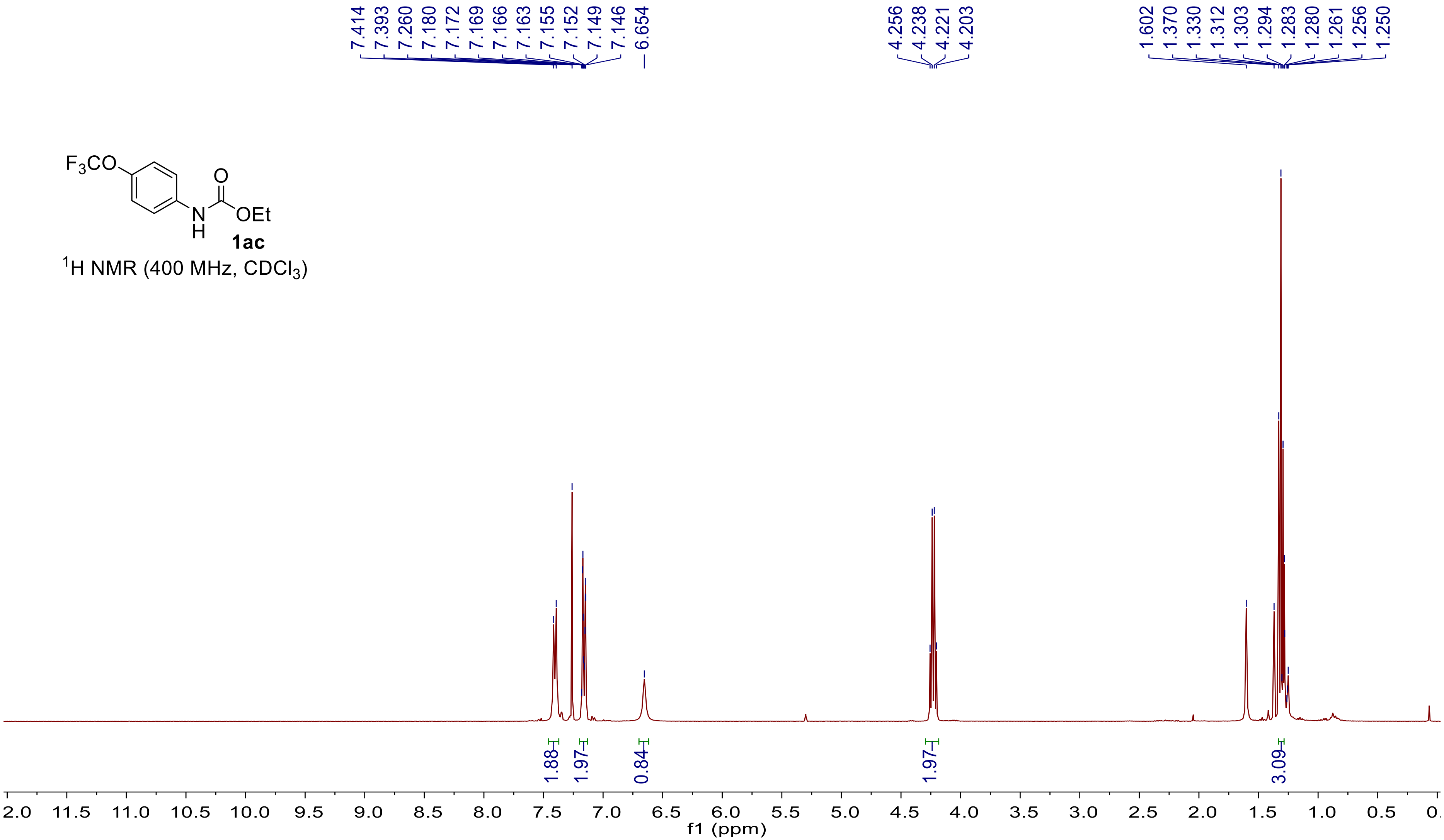

7.414
7.393
7.260
7.180
7.172
7.169
7.166
7.163
7.155
7.152
7.149
7.146
6.654
4.256
4.238
4.221
4.203
1.602
1.370
1.330
1.312
1.303
1.294
1.283
1.280
1.261
1.256
1.250
F_3CO
O
N
H
OEt
1ac
^{1}H NMR (400 MHz, $CDCl_3$)
1.88
1.97
0.84
1.97
3.09
f1 (ppm)

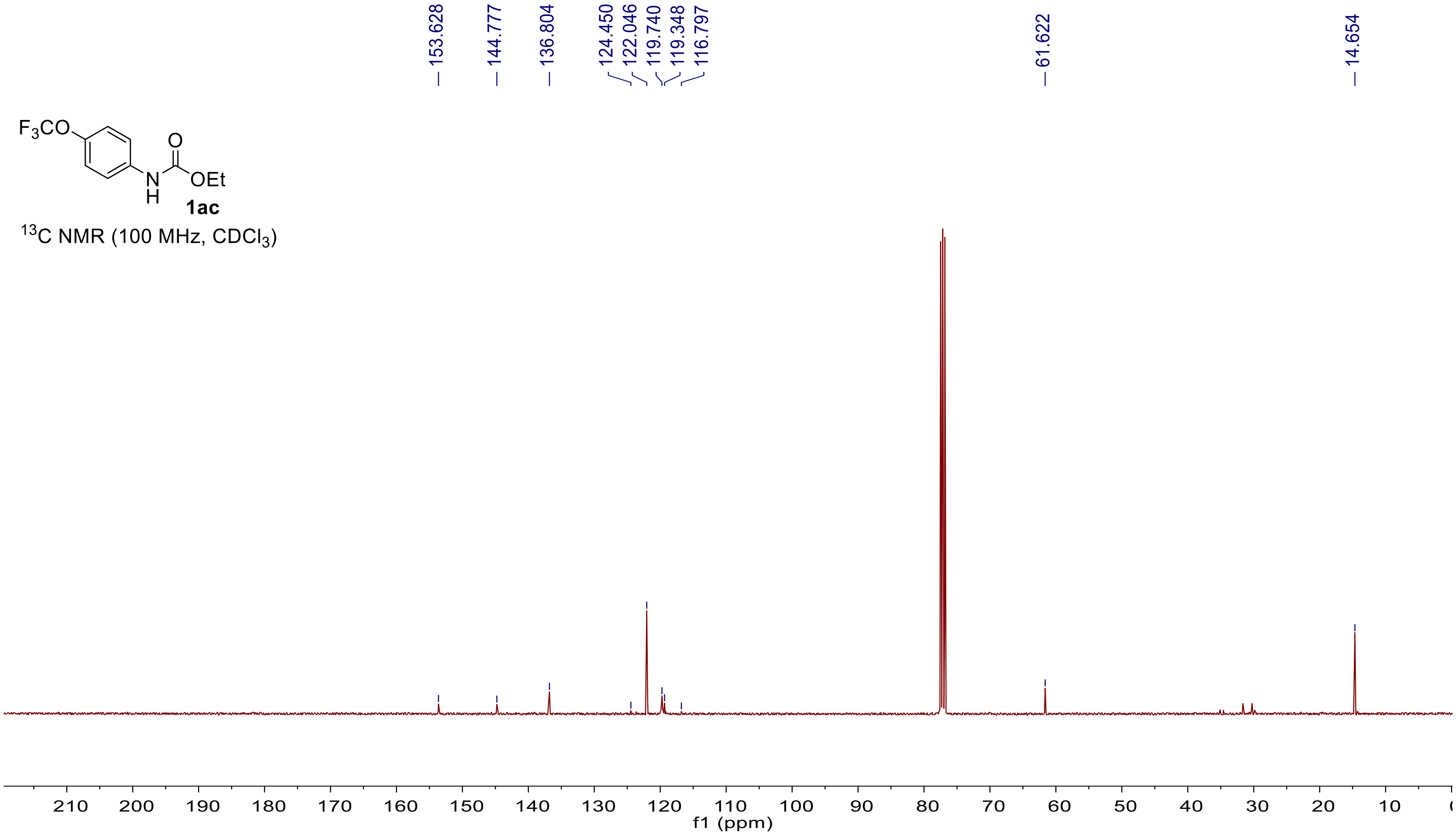
F3CO
O
N
H
OEt
1ac
13C NMR (100 MHz, CDCl3)
153.628
144.777
136.804
124.450
122.046
119.740
119.348
116.797
61.622
14.654
210
200
190
180
170
160
150
140
130
120
110
100
90
80
70
60
50
40
30
20
10
f1 (ppm)

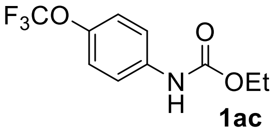

1ac

^{19}F NMR (376 MHz, $CDCl_3$)

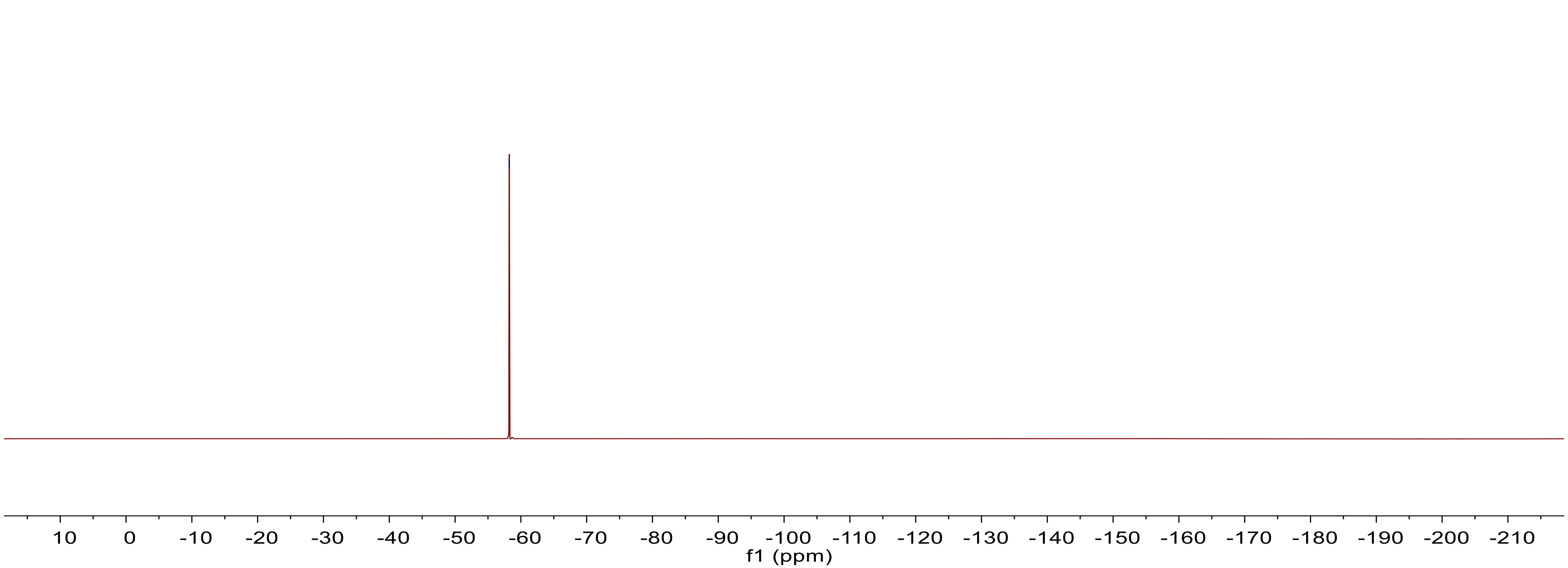

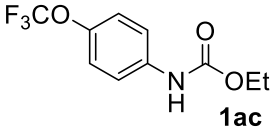

1ac

HRMS (ESI)

SCIEX OS version: 3.0.0.3339 **Printed by:** DESKTOP-SI1BPI6/CZHG
Workstation ID: DESKTOP-SI1BPI6 **Printed on:** 7/13/2024 3:37:46 PM

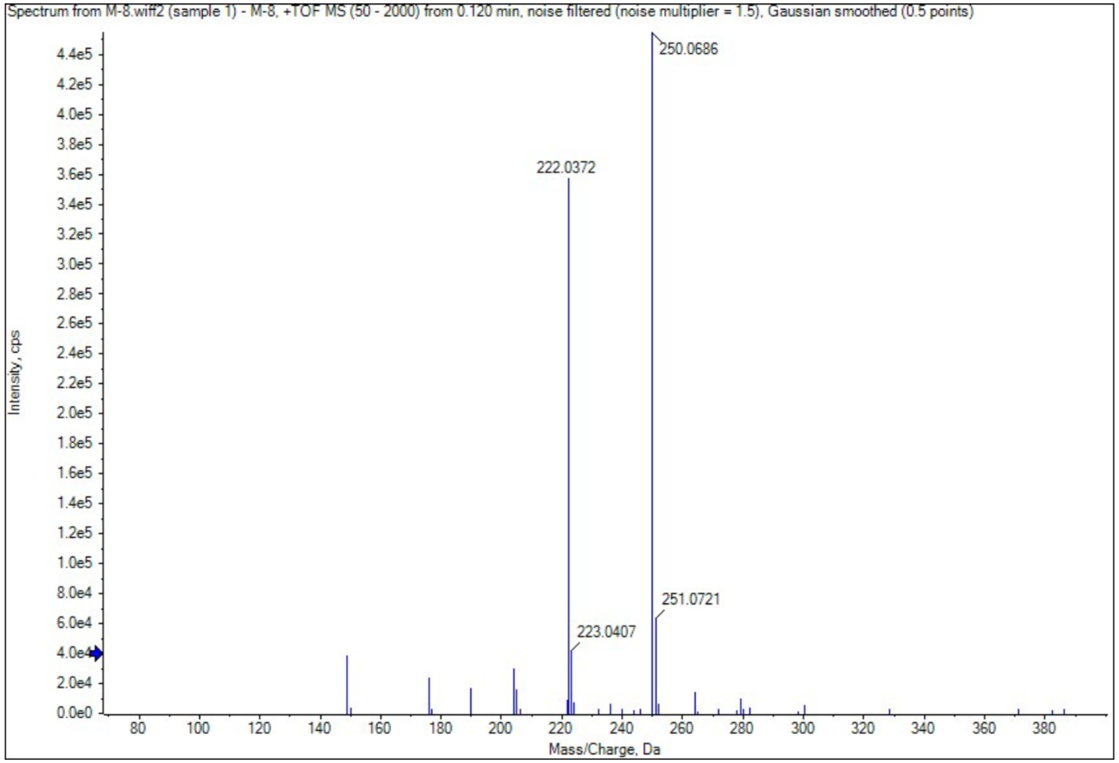

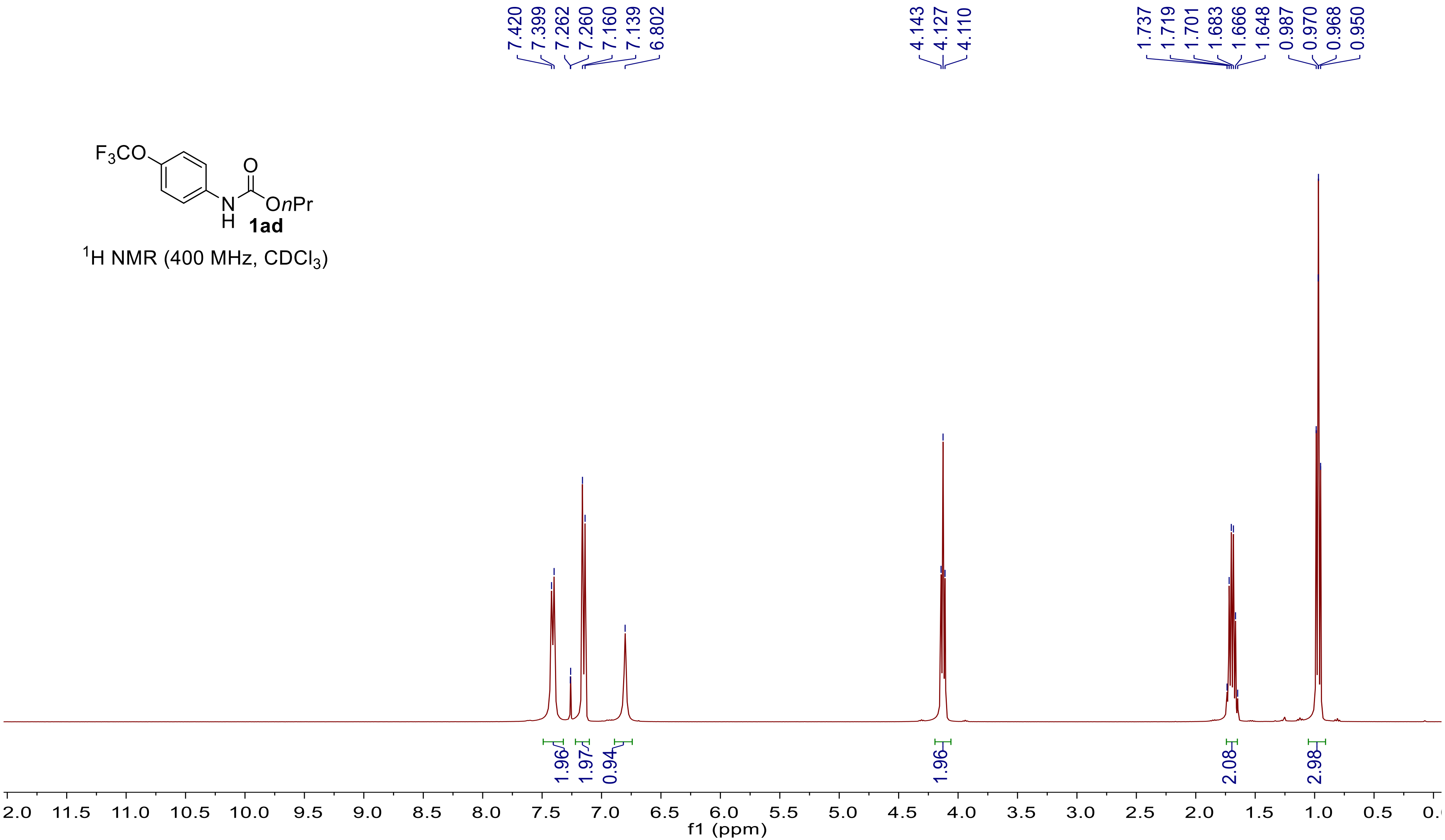

^{1}H NMR (400 MHz, $CDCl_3$)

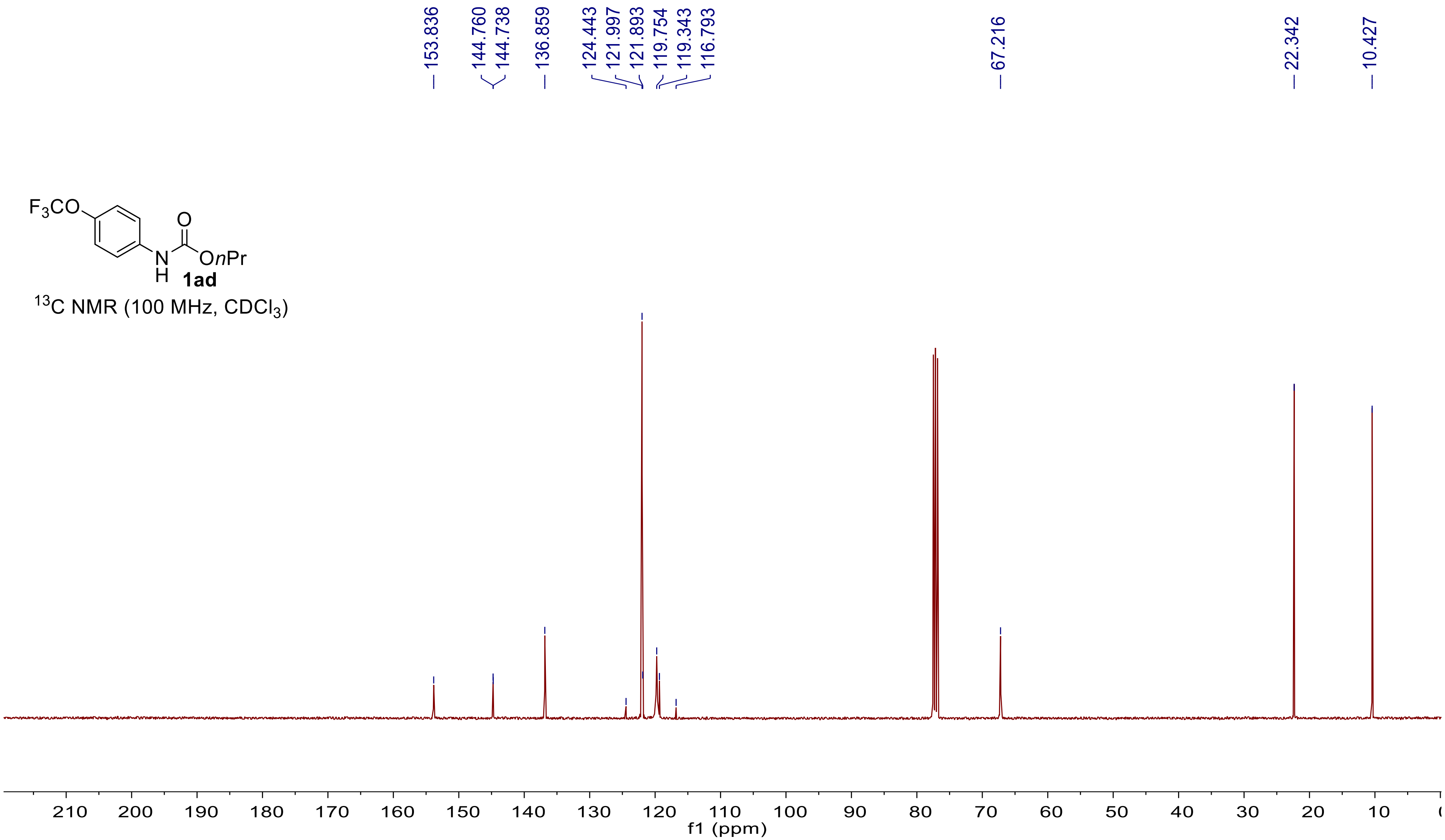

153.836
144.760
144.738
136.859
124.443
121.997
121.893
119.754
119.343
116.793
67.216
22.342
10.427
F3CO
O
N
H
OnPr
1ad
13C NMR (100 MHz, CDCl3)
210 200 190 180 170 160 150 140 130 120 110 100 90 80 70 60 50 40 30 20 10
f1 (ppm)

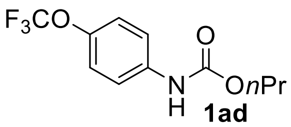

^{19}F NMR (376 MHz, $CDCl_3$)

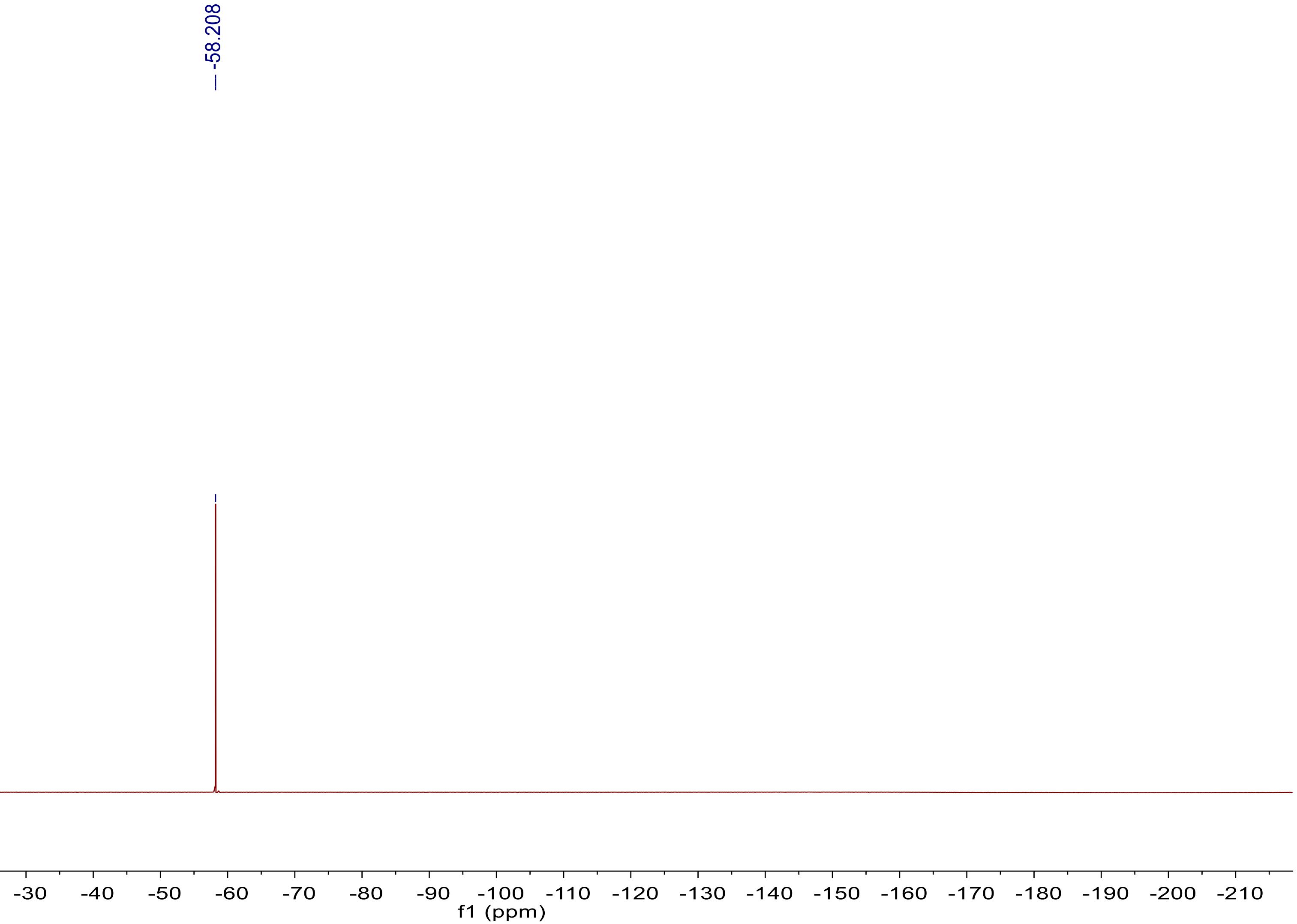

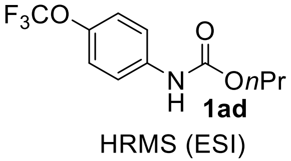

1ad

HRMS (ESI)

SCIEX OS version: 3.0.0.3339 **Printed by:** DESKTOP-SI1BPI6/CZHG

Workstation ID: DESKTOP-SI1BPI6 **Printed on:** 7/13/2024 3:37:08 PM

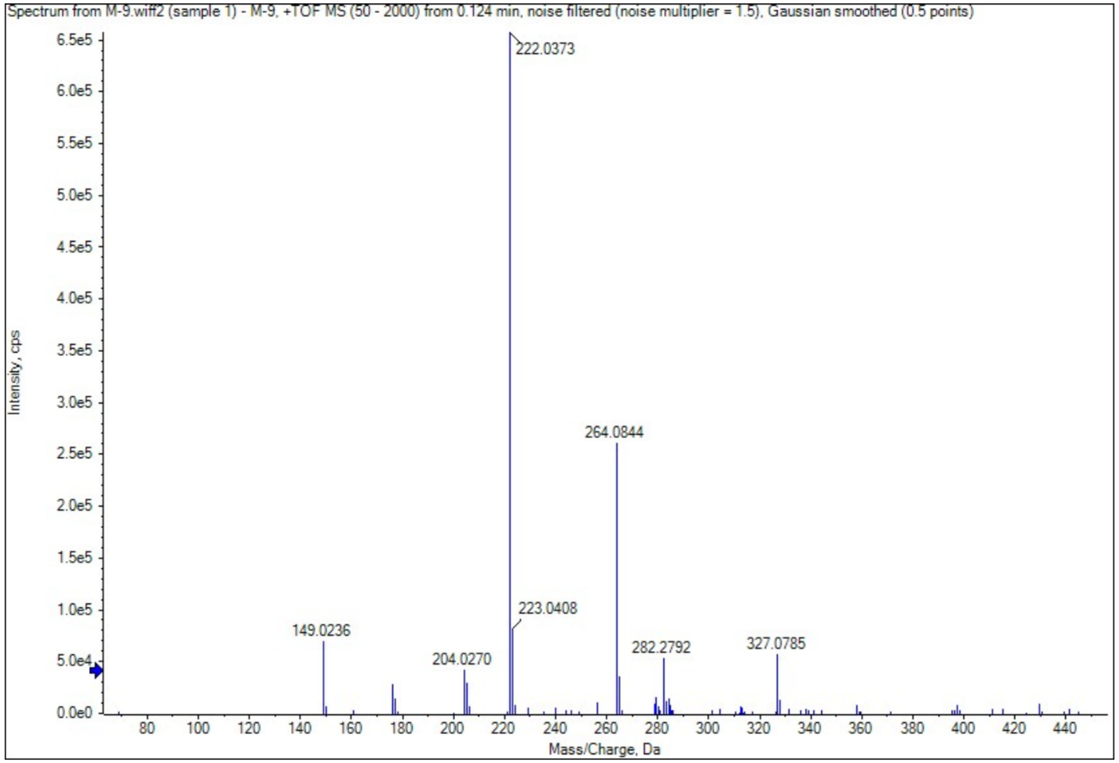

Page 1 of 1

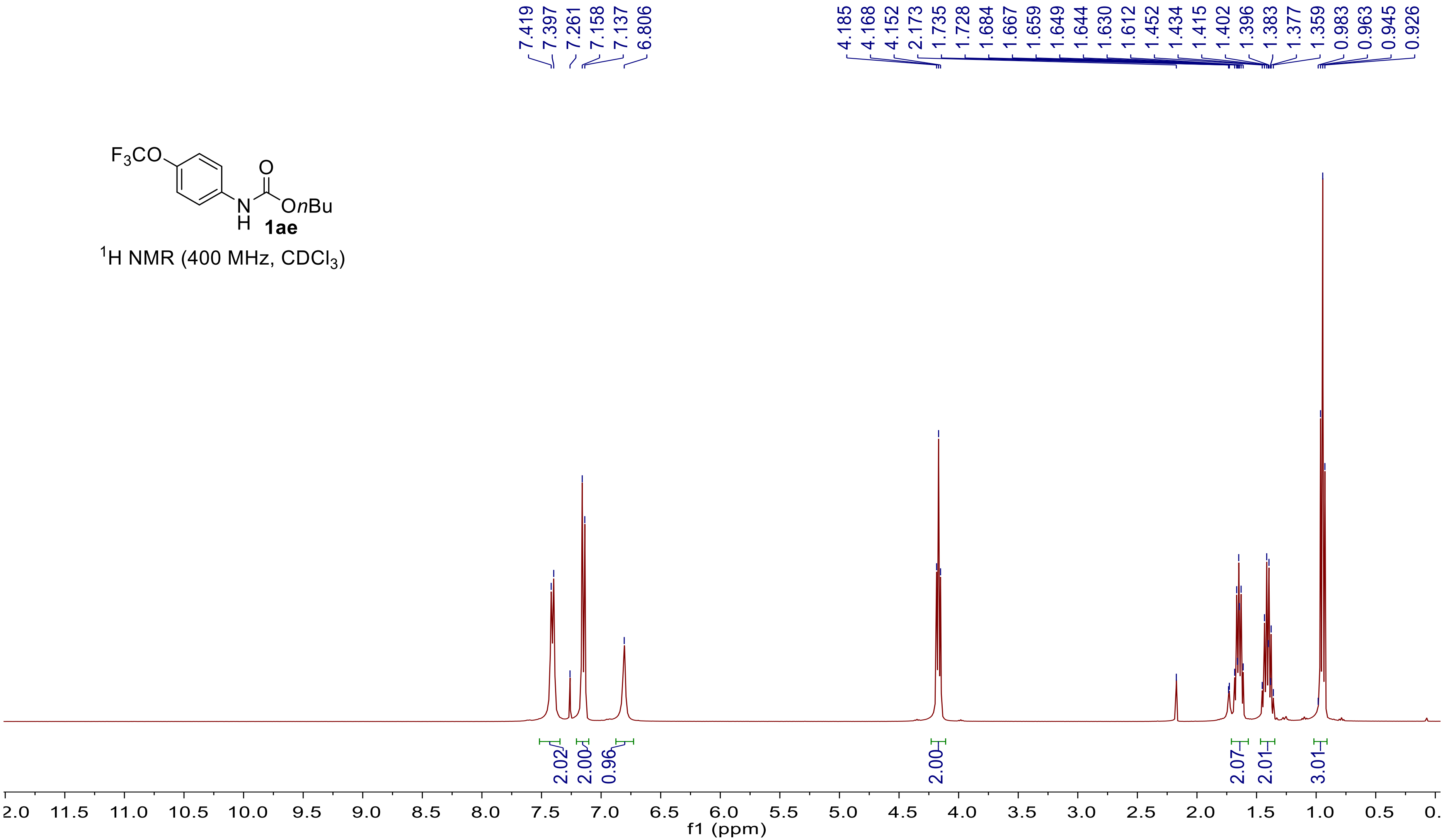
7.419
7.397
7.261
7.158
7.137
6.806
4.185
4.168
4.152
2.173
1.735
1.728
1.684
1.667
1.659
1.649
1.644
1.630
1.612
1.452
1.434
1.415
1.402
1.396
1.383
1.377
1.359
0.983
0.963
0.945
0.926
F3CO
O
N
H
OnBu
1ae
1H NMR (400 MHz, CDCl3)
2.02
2.00
0.96
2.00
2.07
2.01
3.01
f1 (ppm)

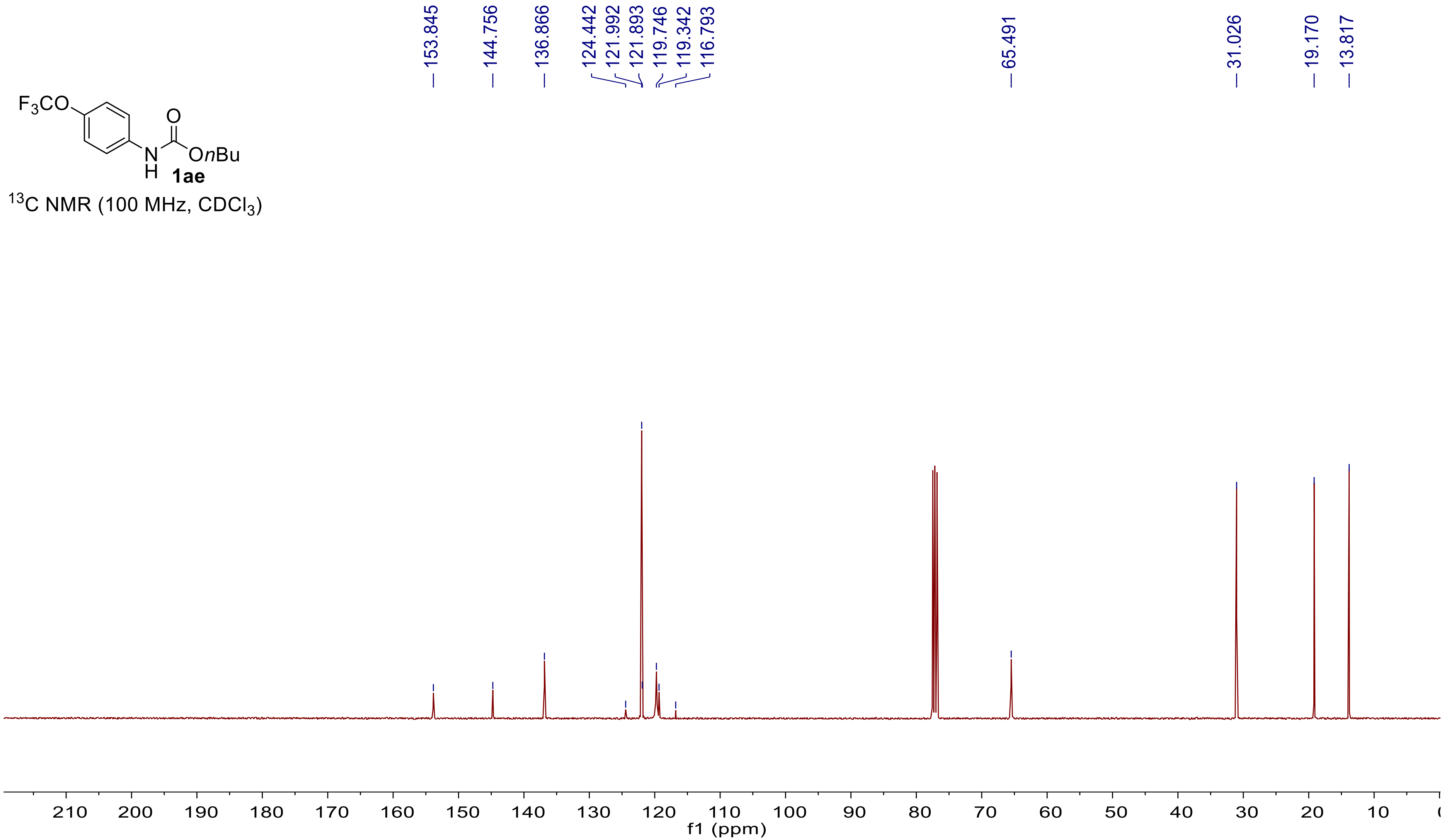
F3CO
O
N
H
OnBu
1ae
13C NMR (100 MHz, CDCl3)
153.845
144.756
136.866
124.442
121.992
121.893
119.746
119.342
116.793
65.491
31.026
19.170
13.817
210
200
190
180
170
160
150
140
130
120
110
100
90
80
70
60
50
40
30
20
10
f1 (ppm)

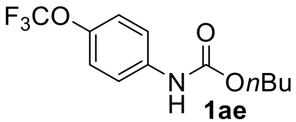

1ae

^{19}F NMR (376 MHz, $CDCl_3$)

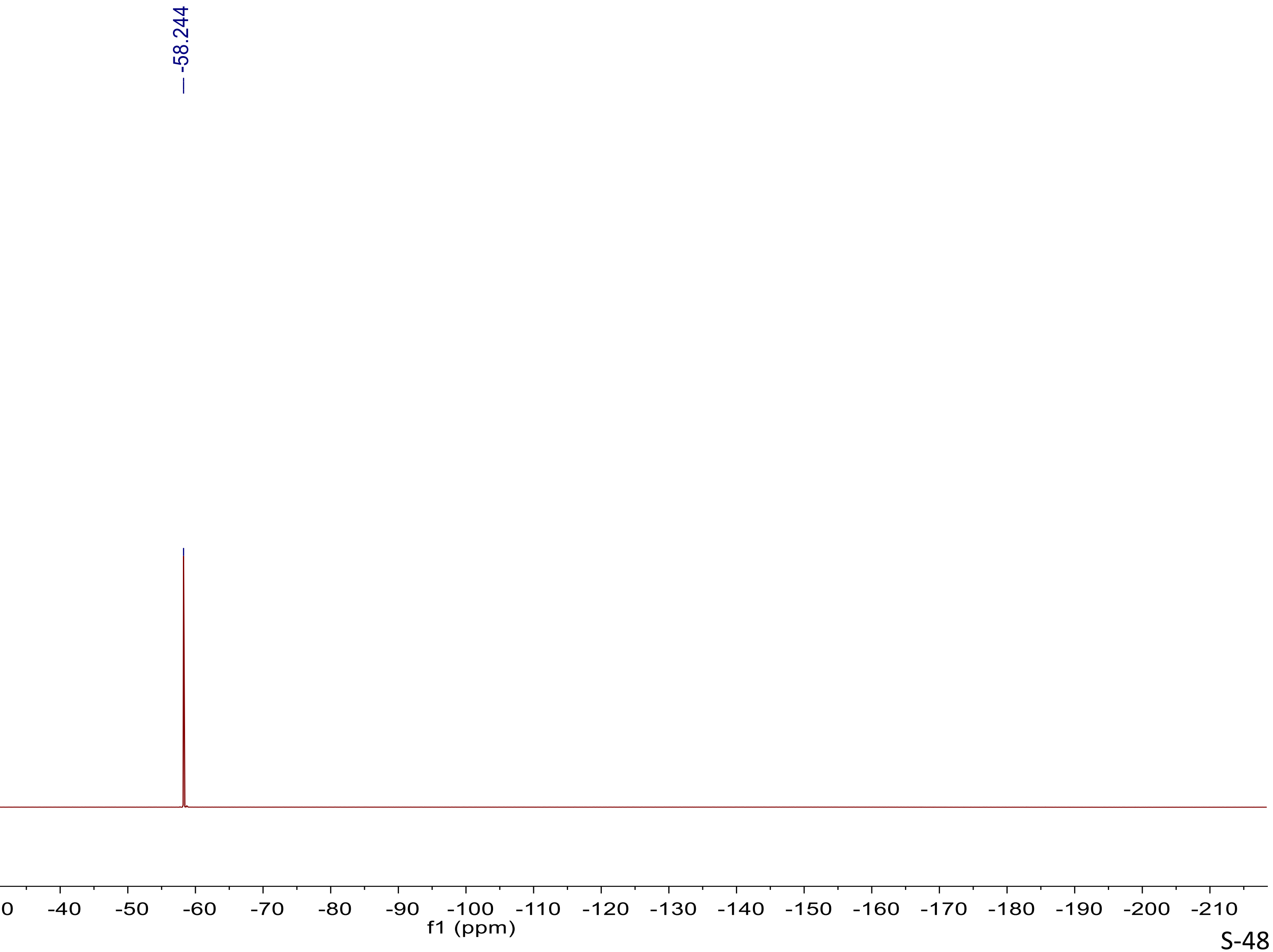

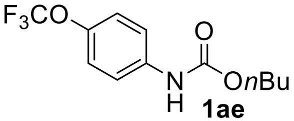

1ae

HRMS (ESI)

SCIEX OS version: 3.0.0.3339 **Printed by:** DESKTOP-SI1BPI6/CZHG
Workstation ID: DESKTOP-SI1BPI6 **Printed on:** 1/27/2024 2:18:31 PM

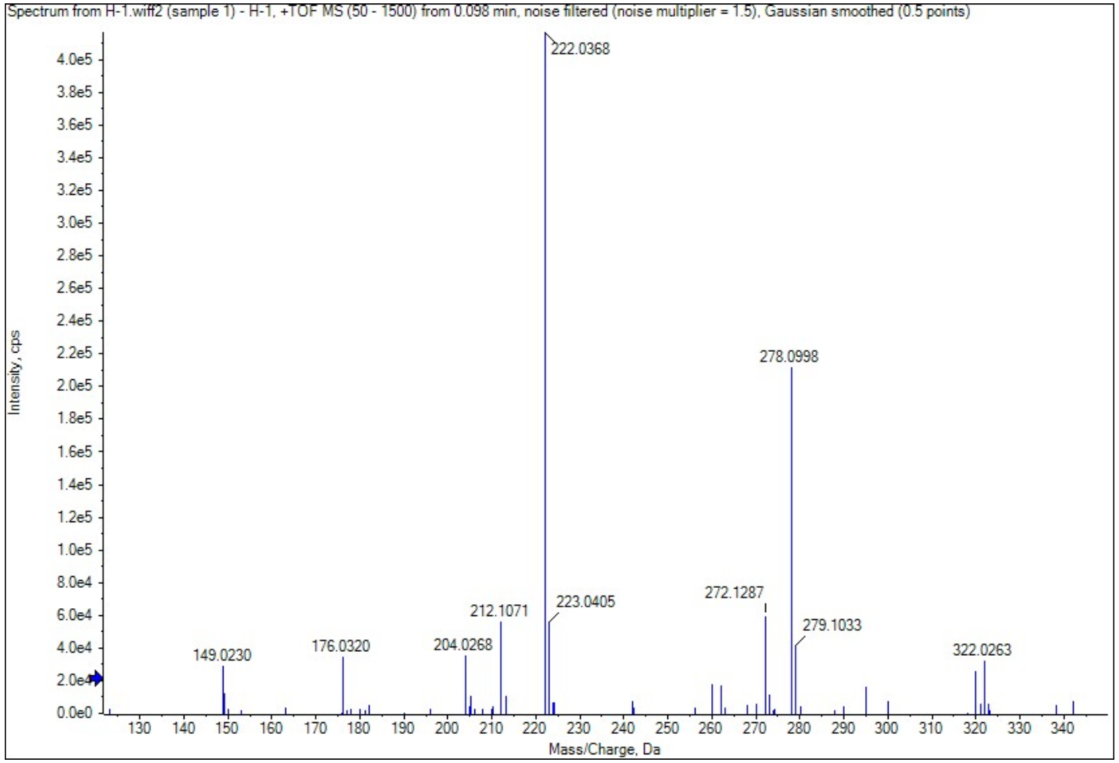

Page 1 of 1

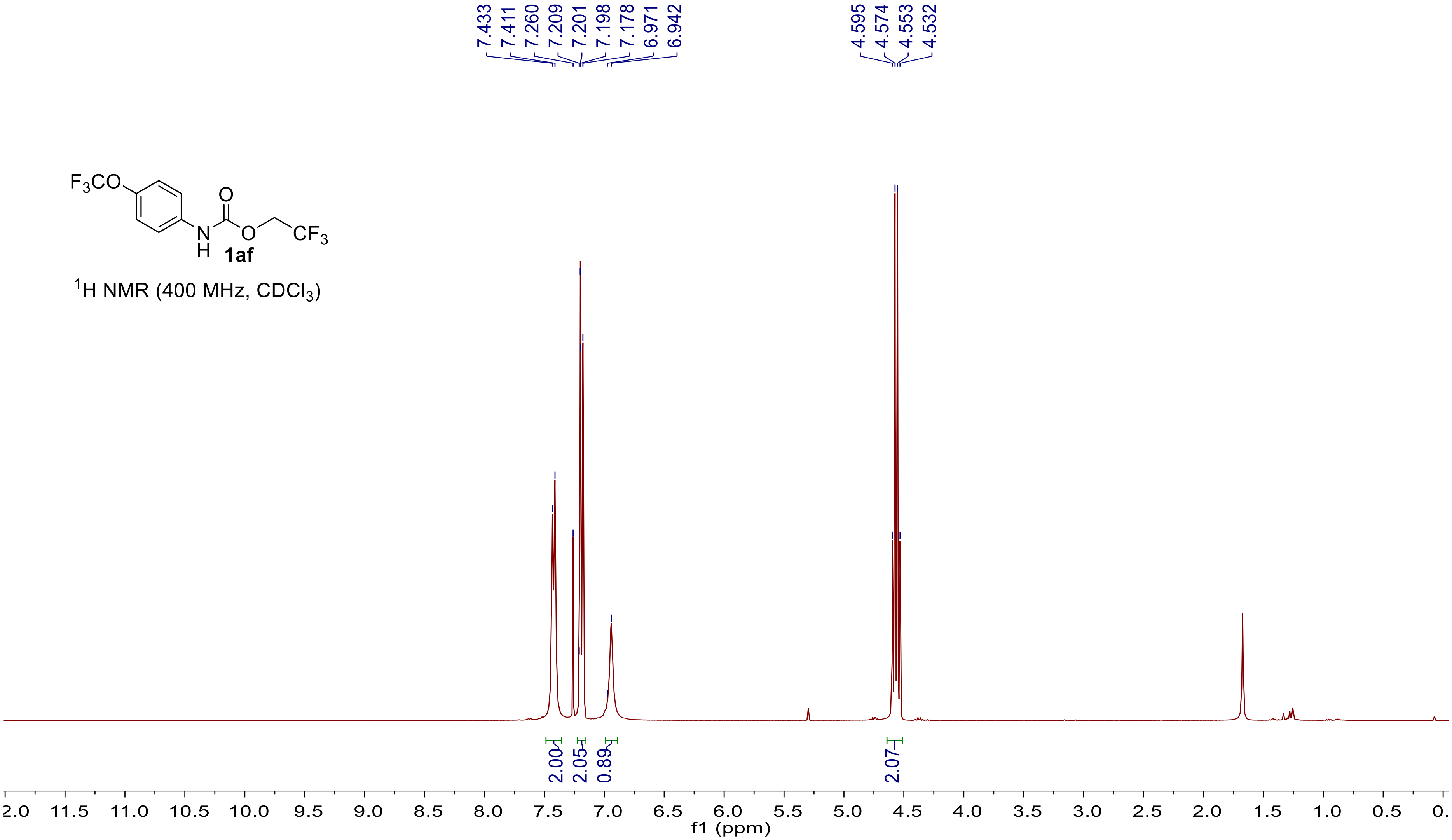
7.433
7.411
7.260
7.209
7.201
7.198
7.178
6.971
6.942
4.595
4.574
4.553
4.532
F3CO
O
N
H
O
CF3
1af
1H NMR (400 MHz, CDCl3)
2.00
2.05
0.89
2.07
2.0
11.5
11.0
10.5
10.0
9.5
9.0
8.5
8.0
7.5
7.0
6.5
6.0
5.5
5.0
4.5
4.0
3.5
3.0
2.5
2.0
1.5
1.0
0.5
0.
f1 (ppm)

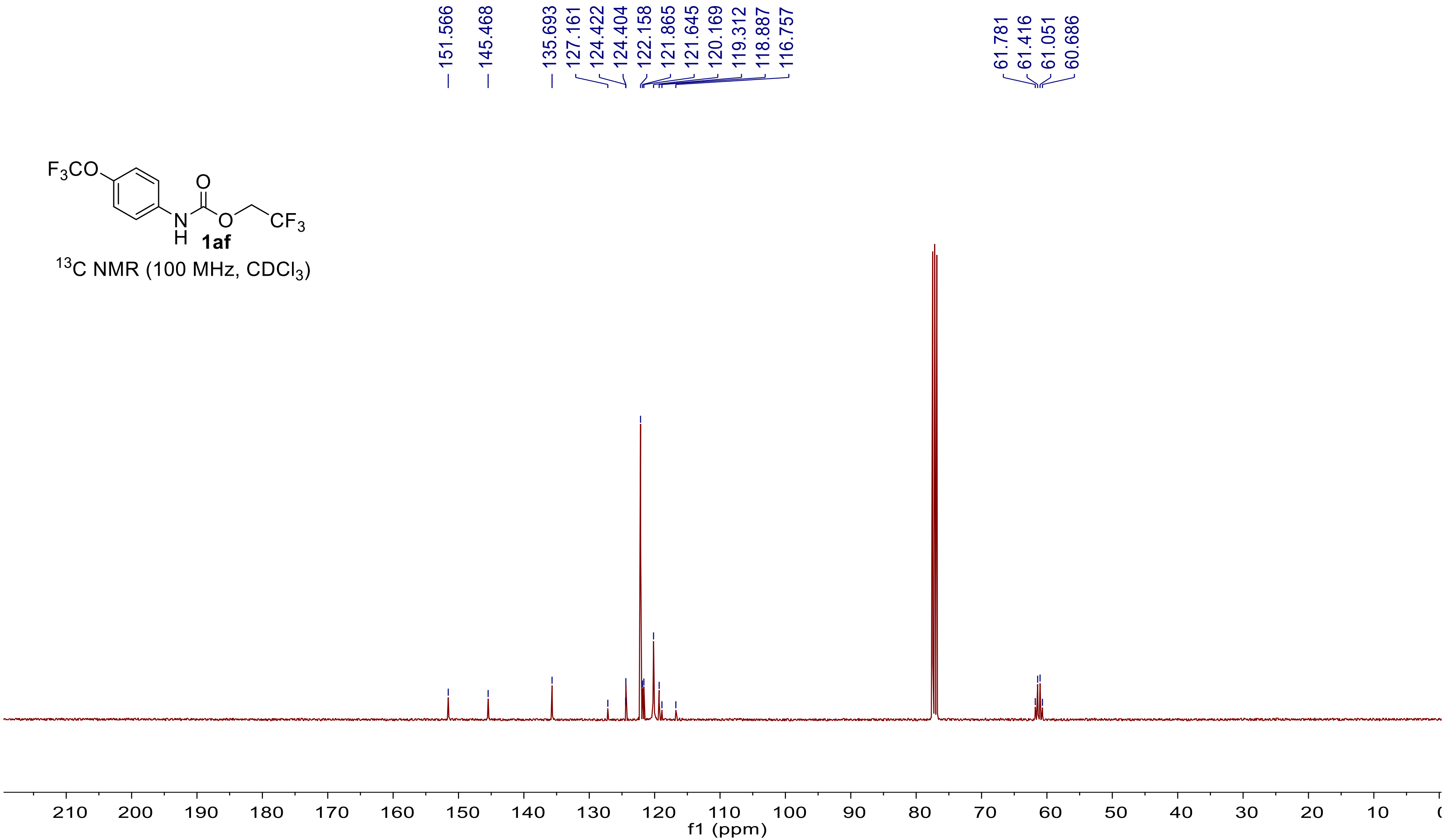

F3CO
O
N
H
O
CF3
1af
13C NMR (100 MHz, CDCl3)
151.566
145.468
135.693
127.161
124.422
124.404
122.158
121.865
121.645
120.169
119.312
118.887
116.757
61.781
61.416
61.051
60.686
210 200 190 180 170 160 150 140 130 120 110 100 90 80 70 60 50 40 30 20 10 0
f1 (ppm)

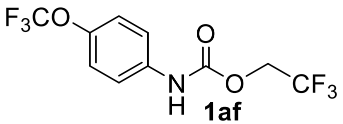

1af

^{19}F NMR (376 MHz, $CDCl_3$)

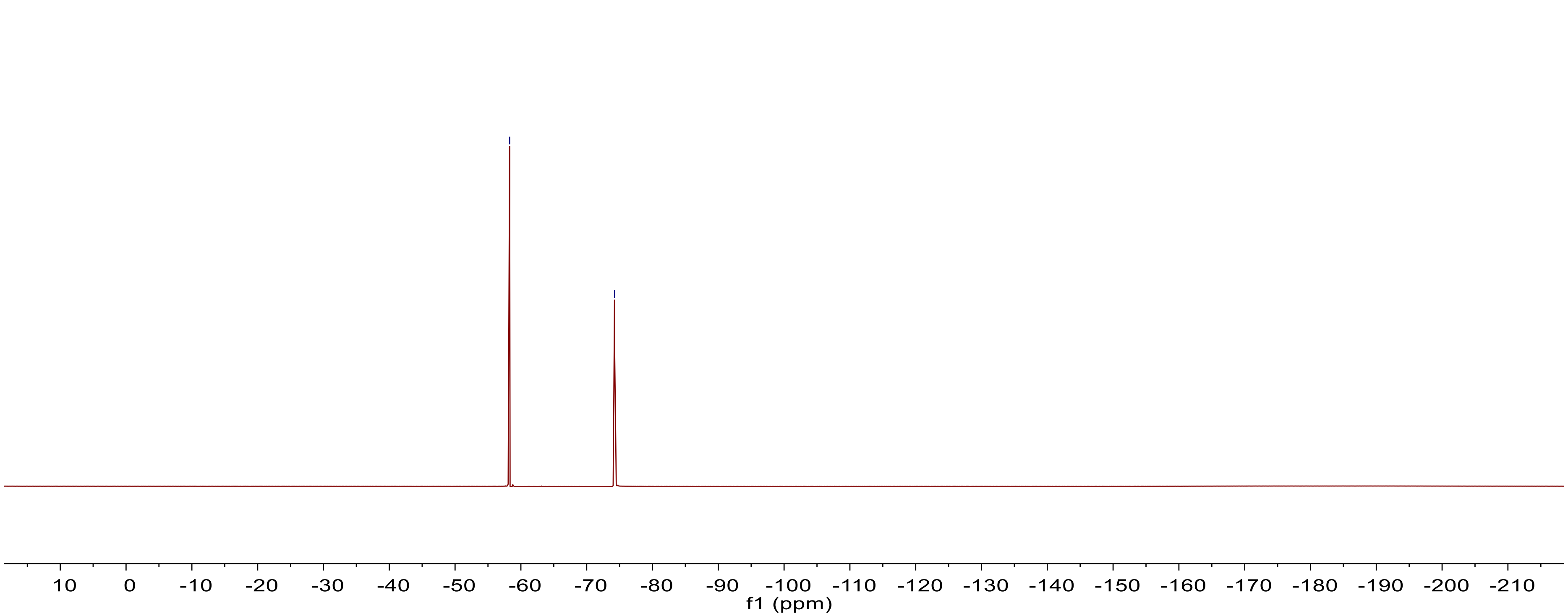

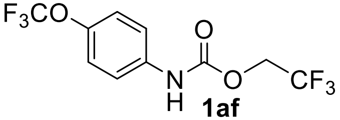

1af

HRMS (ESI)

SCIEX

SCIEX OS version: 3.0.0.3339 **Printed by:** DESKTOP-SI1BPI6/CZHG

Workstation ID: DESKTOP-SI1BPI6 **Printed on:** 1/27/2024 2:20:19 PM

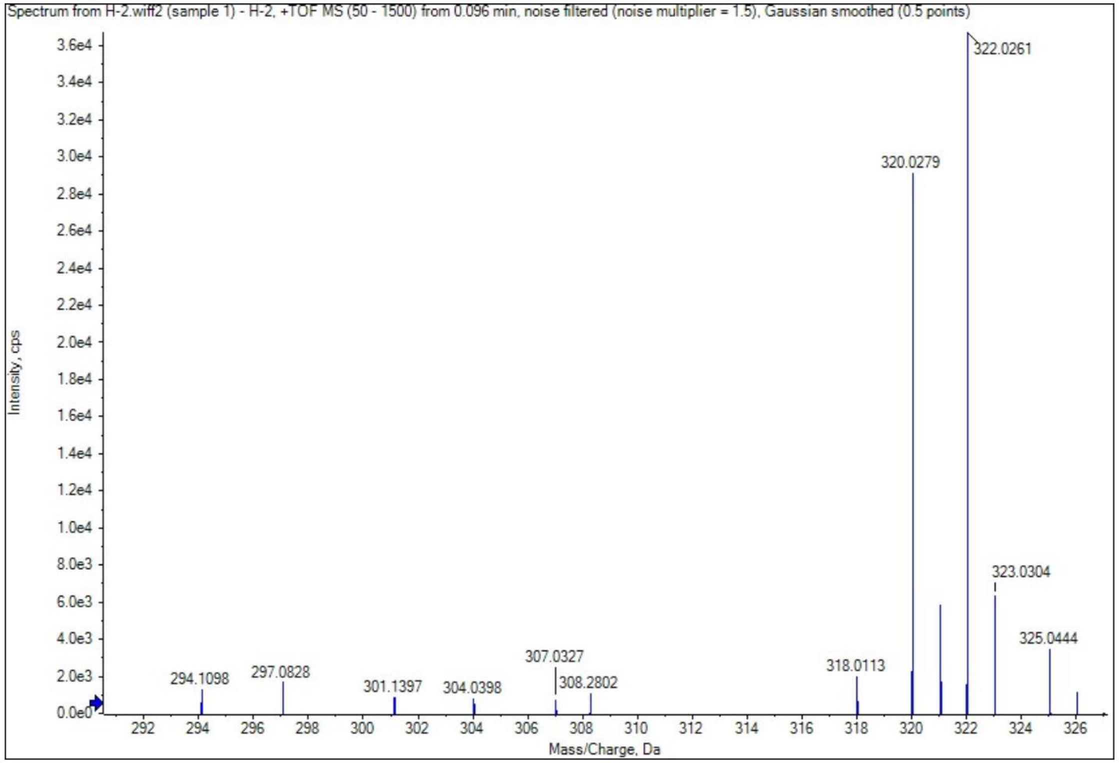

Page 1 of 1

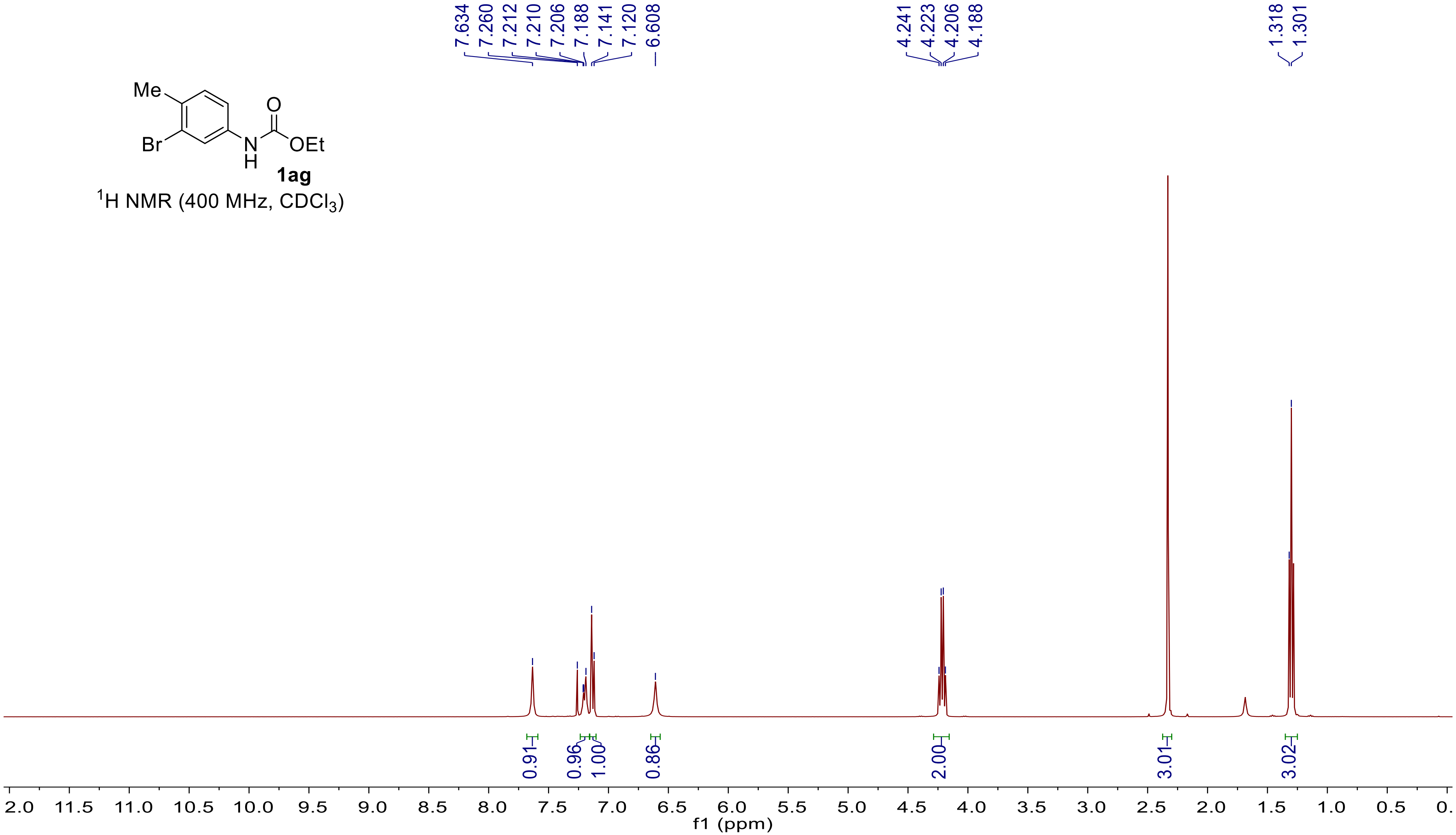

7.634
7.260
7.212
7.210
7.206
7.188
7.141
7.120
6.608
4.241
4.223
4.206
4.188
1.318
1.301
Me
O
Br
N
H
OEt
1ag
^{1}H NMR (400 MHz, CDCl$_3$)
0.91
0.96
1.00
0.86
2.00
3.01
3.02
2.0
11.5
11.0
10.5
10.0
9.5
9.0
8.5
8.0
7.5
7.0
6.5
6.0
5.5
5.0
4.5
4.0
3.5
3.0
2.5
2.0
1.5
1.0
0.5
0.
f1 (ppm)

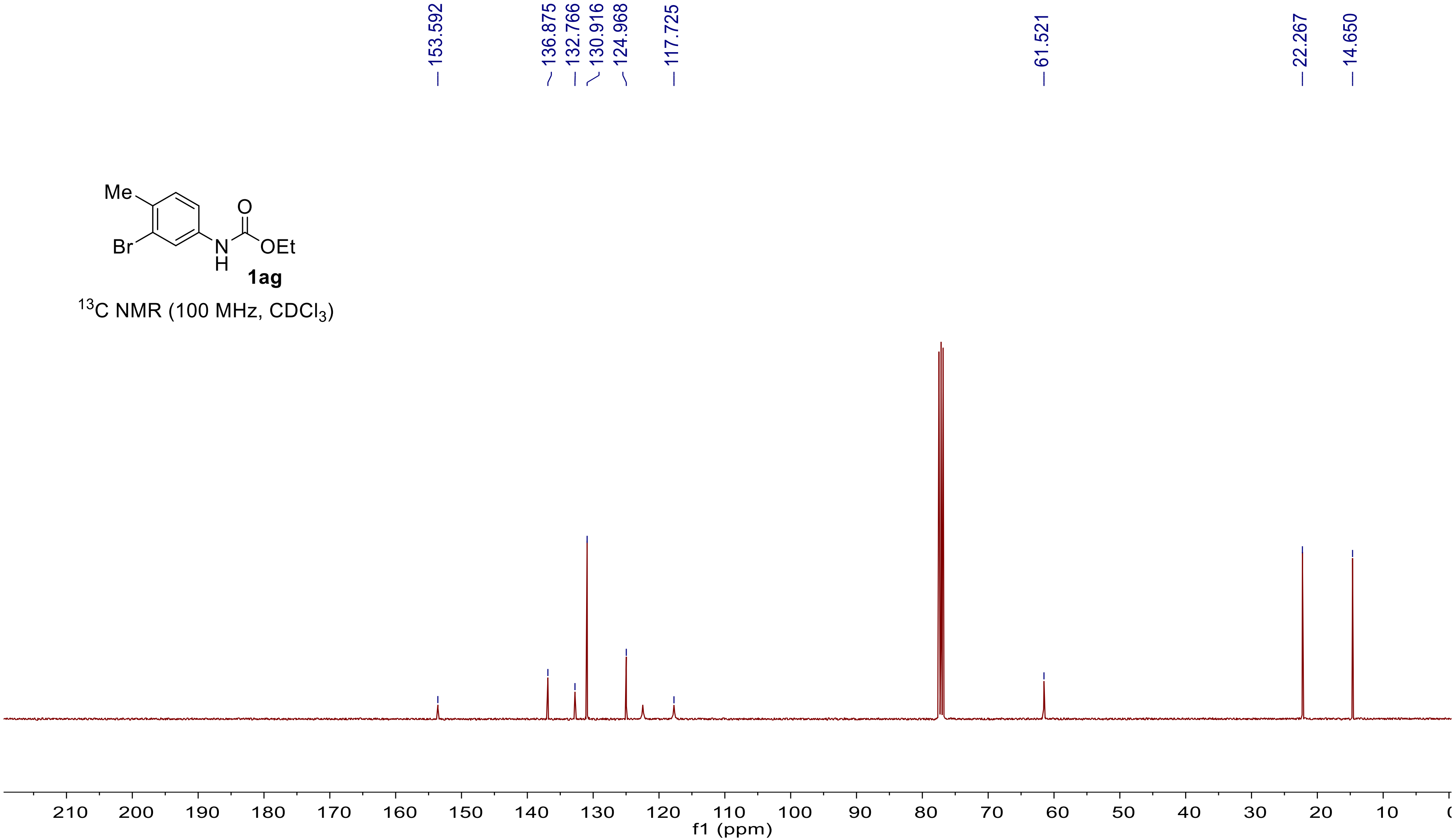

1ag

^{13}C NMR (100 MHz, $CDCl_3$)

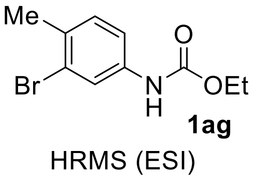
Me
O
Br
N
H
OEt

1ag

HRMS (ESI)

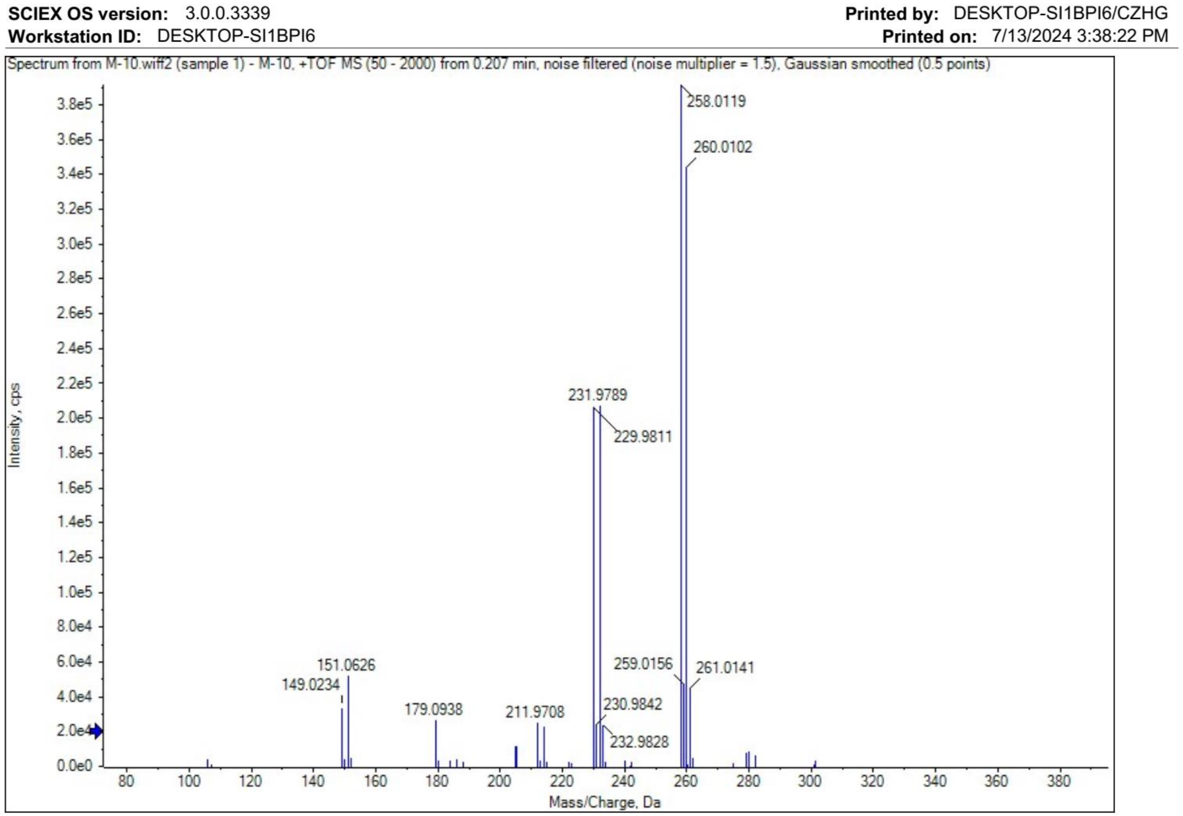
SCIEX OS version: 3.0.0.3339
Workstation ID: DESKTOP-SI1BPI6
Printed by: DESKTOP-SI1BPI6/CZHG
Printed on: 7/13/2024 3:38:22 PM
Spectrum from M-10.wiff2 (sample 1) - M-10, +TOF MS (50 - 2000) from 0.207 min, noise filtered (noise multiplier = 1.5), Gaussian smoothed (0.5 points)
258.0119
260.0102
231.9789
229.9811
259.0156
261.0141
151.0626
149.0234
179.0938
211.9708
230.9842
232.9828
Intensity, cps
Mass/Charge, Da

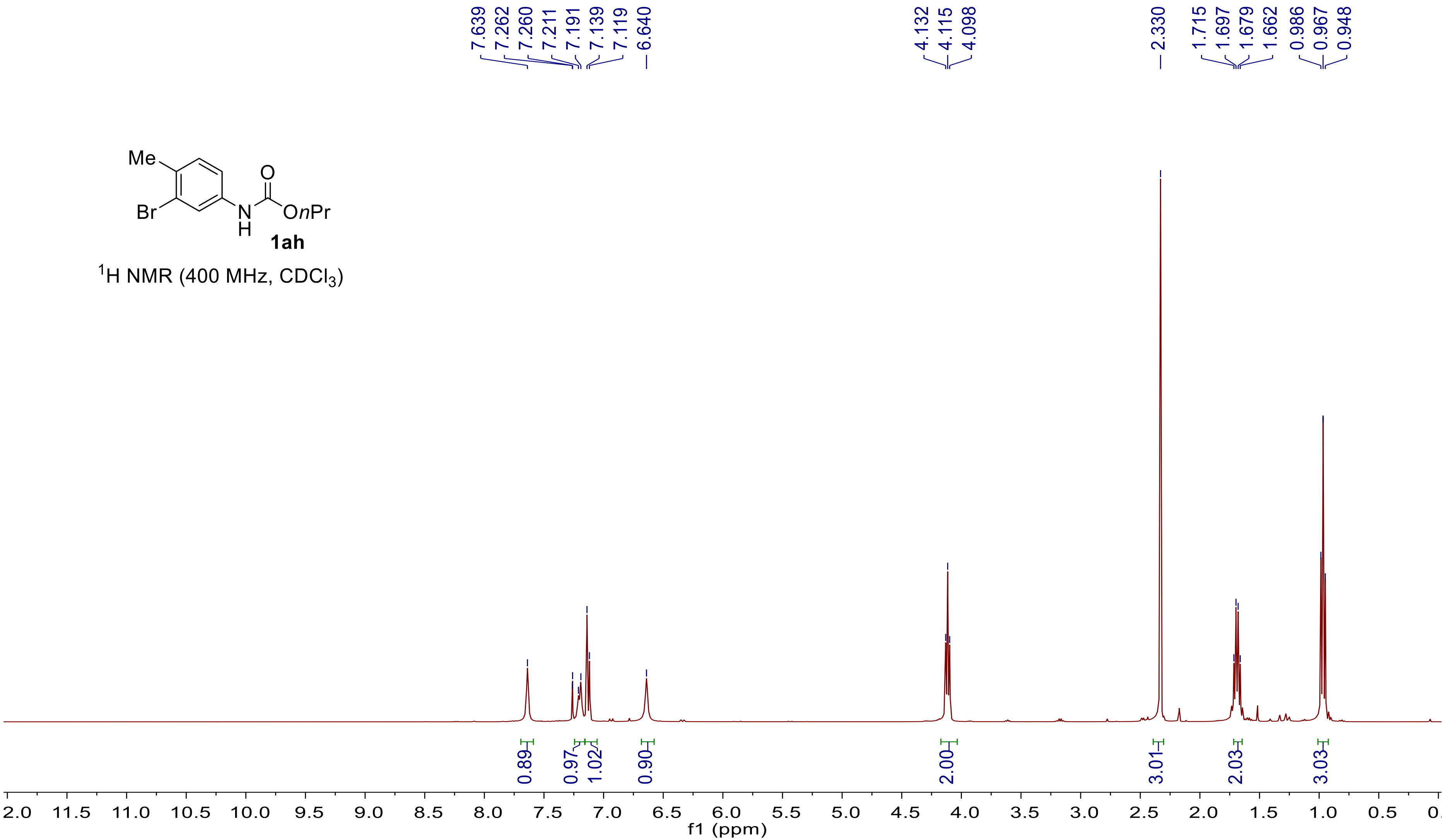

7.639
7.262
7.260
7.211
7.191
7.139
7.119
6.640
4.132
4.115
4.098
2.330
1.715
1.697
1.679
1.662
0.986
0.967
0.948
Me
O
Br
N
H
OnPr
1ah
¹H NMR (400 MHz, CDCl₃)
0.89
0.97
1.02
0.90
2.00
3.01
2.03
3.03
f1 (ppm)

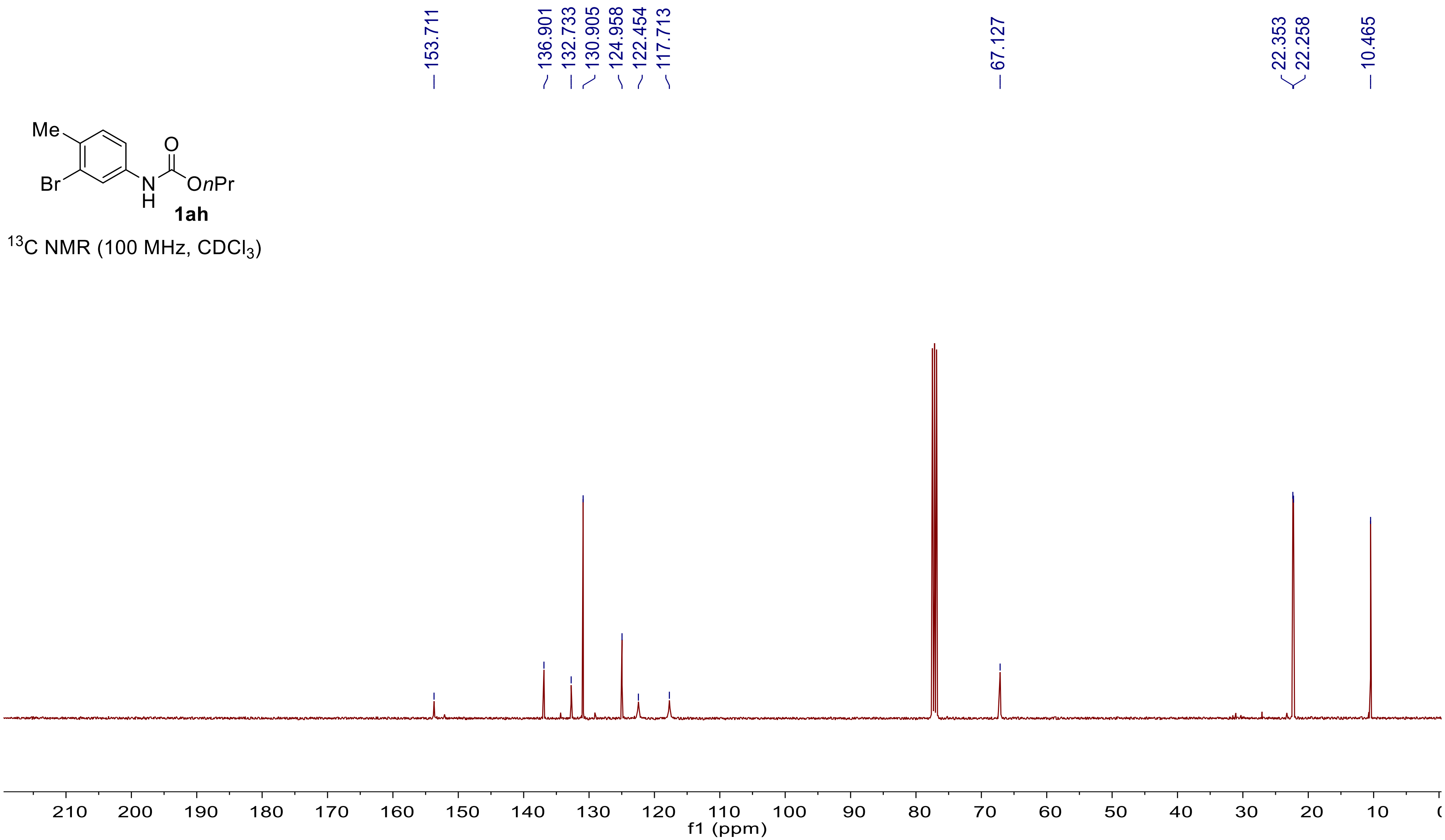

^{13}C NMR (100 MHz, $CDCl_3$)

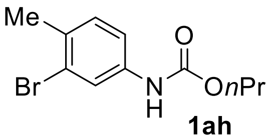
Me
O
Br
N
H
OnPr

1ah

HRMS (ESI)

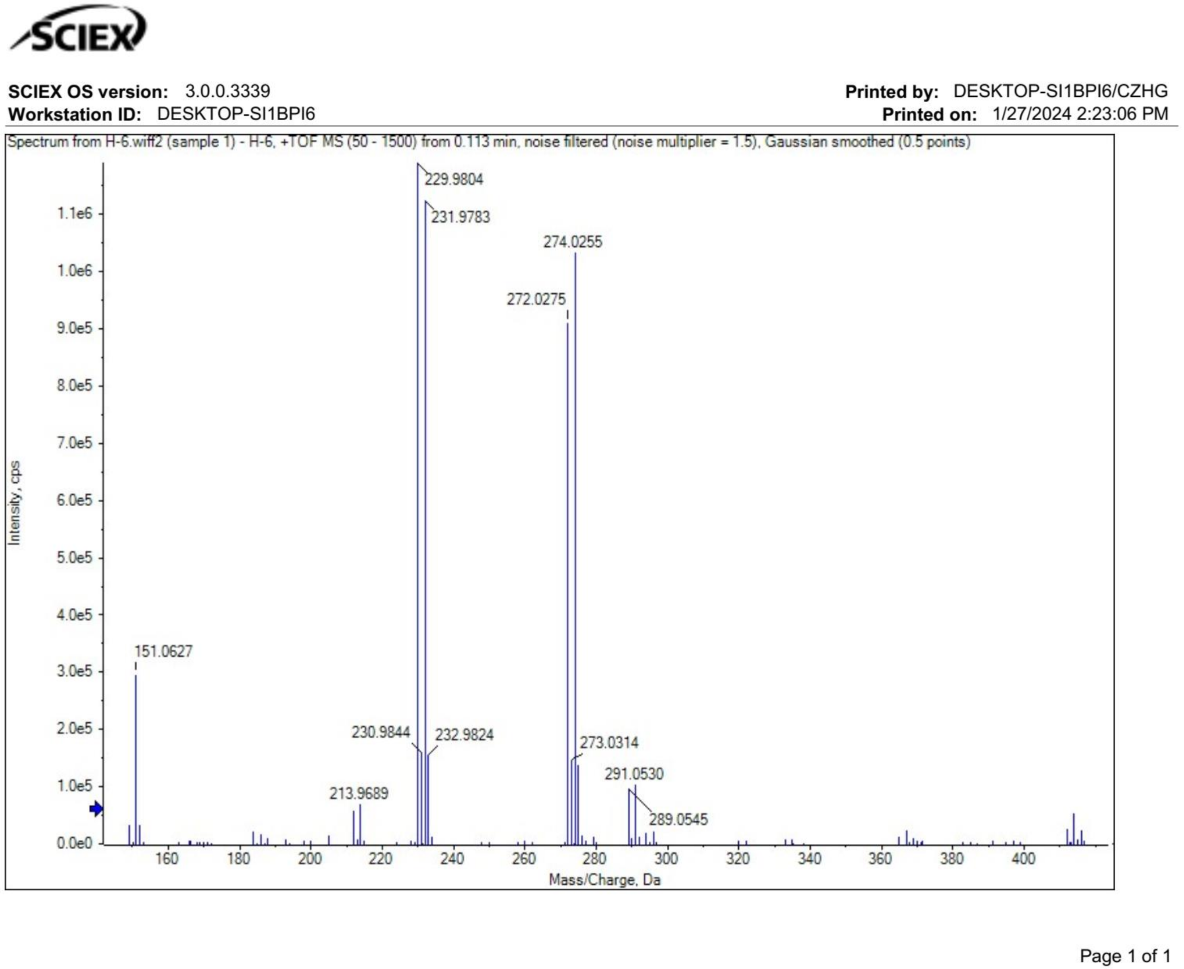
SCIEX OS version: 3.0.0.3339
Workstation ID: DESKTOP-SI1BPI6
Printed by: DESKTOP-SI1BPI6/CZHG
Printed on: 1/27/2024 2:23:06 PM
Spectrum from H-6.wiff2 (sample 1) - H-6, +TOF MS (50 - 1500) from 0.113 min, noise filtered (noise multiplier = 1.5), Gaussian smoothed (0.5 points)
Intensity, cps
Mass/Charge, Da
229.9804
231.9783
274.0255
272.0275
151.0627
230.9844
232.9824
273.0314
291.0530
213.9689
289.0545
Page 1 of 1
